# NUPR1 inhibition prevents mitotic catastrophe: a novel therapeutic strategy for podocyte injury in diabetic kidney disease

**DOI:** 10.1038/s41419-026-09152-8

**Published:** 2026-08-12

**Authors:** Yating Cai, Li Zhang, Lu’an Li, Weiteng Wang, Yuanhan Chen, Xingchen Zhao, Jiaying Li, Jia Wen, Dantong Li, Manlu Xiao, Ruizhao Li, Xinling Liang

**Affiliations:** 1 Department of Nephrology, Guangdong Provincial People’s Hospital, Guangdong Academy of Medical Sciences, Southern Medical University, Guangzhou, China; 2 Ganzhou Hospital of Guangdong Provincial People’s Hospital, Ganzhou Municipal Hospital, Ganzhou, China; 3 Guangdong Provincial People’s Hospital, Zhuhai Hospital (Jinwan Central Hospital of Zhuhai), Zhuhai, China

**Keywords:** Cell death, Mechanisms of disease, Glomerular diseases, Diabetes complications

## Abstract

Mitotic catastrophe (MC) is a frequent and decisive event that drives podocyte loss in diabetic kidney disease (DKD), yet its molecular regulation remains poorly understood. Here, we demonstrate that nuclear protein 1 (NUPR1) is both necessary and sufficient to induce MC in podocytes. NUPR1 was significantly upregulated in podocytes from human DKD biopsies, and its expression level positively correlated with albuminuria. Overexpression of NUPR1 in DKD mice exacerbated podocyte injury and increased albuminuria. Functional analyses revealed that NUPR1 promotes aberrant podocyte entry into the G2/M phase, thereby triggering MC and aggravating renal injury. Mechanistically, NUPR1 recruits BRD2 to the Cyclin B2 promoter, forming a transcriptional complex that enhances Cyclin B2 expression. Genetic or pharmacological disruption of this NUPR1-BRD2 interaction suppressed Cyclin B2 expression, prevented MC, and preserved podocyte numbers. Therapeutically, pharmacological inhibition of NUPR1 using ZZW-115 significantly alleviated albuminuria, reduced mesangial matrix expansion, and mitigated podocyte injury in DKD mice, without impacting systemic glucose levels. Together, these findings identify the NUPR1-BRD2/Cyclin B2 axis as a key molecular driver of podocyte MC and establish NUPR1 as a promising therapeutic target for slowing DKD progression.

**Figure:**
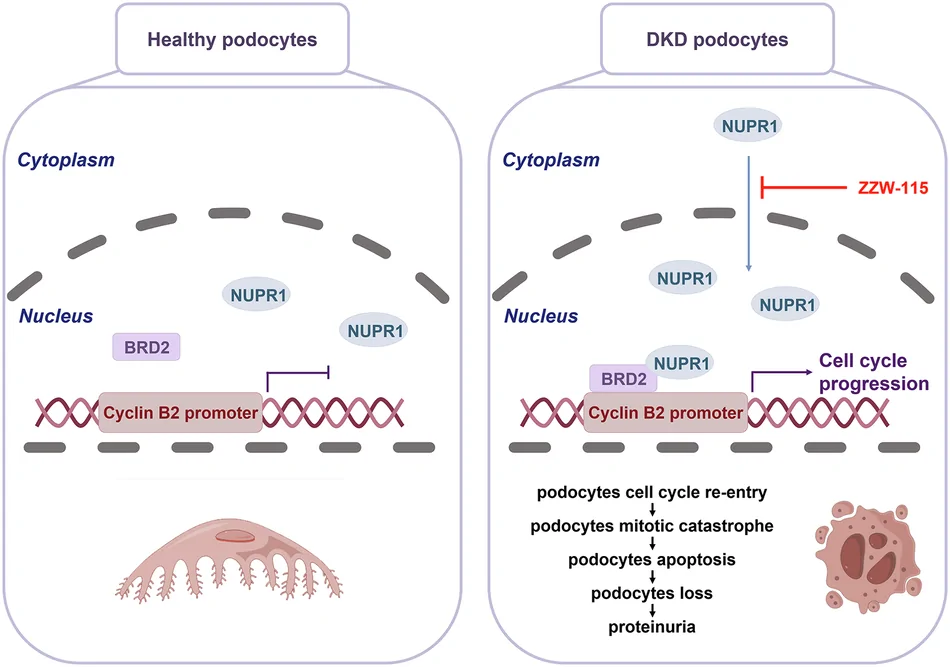

## Introduction

Diabetic kidney disease (DKD) is one of the primary causes of end-stage renal disease globally [1]. Podocytes, which are highly specialized and terminally differentiated cells, are essential components of the glomerular filtration barrier and play a crucial role in the progression of DKD [2]. Damage to and loss of these cells compromise the integrity of the filtration barrier, resulting in proteinuria and accelerating the progression of the disease [3]. A variety of pathological processes, such as oxidative stress, autophagy dysfunction, endoplasmic reticulum stress, apoptosis, pyroptosis, and ferroptosis, have been implicated in podocyte injury and death [4, 5]. More recently, research on human podocytes has identified mitotic catastrophe (MC), a form of aberrant cell death characterized by defective chromosome segregation following DNA damage and histologically identified by multinucleation, as another significant contributor to podocyte loss under harmful conditions [6]. Under physiological conditions, mature podocytes remain quiescent. However, under certain pathological stimuli, podocytes can be prompted to re-enter the cell cycle [7]. Due to their unique structural features, podocytes are unable to properly assemble actin-based mitotic spindles while preserving the cytoskeletal integrity of their foot processes [6], consequently, they cannot complete cytoplasmic division, whereas nuclear division can proceed normally. These aneuploid podocytes show heightened susceptibility to injury, increasing their likelihood of detaching from the basement membrane or undergoing apoptosis [8, 9]. This process, termed podocyte MC, ultimately contributes to the development of proteinuria [10, 11].

Nuclear protein 1 (NUPR1) was discovered in 1997 as a transcriptional regulator strongly induced in rat pancreatic acinar cells during acute pancreatitis [12]. Since then, it has emerged as a pleiotropic stress-response factor that governs apoptosis, endoplasmic-reticulum stress, senescence and cell-cycle progression, positioning it at the hub of multiple disease pathways [7, 13–15]. In the kidney, the ATF4-NUPR1 axis amplifies ER stress and drives cyclosporine-A-induced tubular injury [16]. whereas in mesangial cells NUPR1 accelerates proliferation through ERK, JNK/SAPK and PI3K signaling [17]. Pharmacologically, trifluoperazine, an FDA-approved phenothiazine, binds NUPR1 directly, blunts Smad3 activation, suppresses TGF-β-mediated α-SMA expression and collagen synthesis, and ultimately attenuates renal fibrosis by blocking fibroblast activation and tubular EMT [18]. Despite these insights, the downstream network controlled by NUPR1 remains fragmented, and it is unclear whether its transcriptional program is cell-type-specific or conserved across pathologies. Most importantly, its role in podocyte injury and MC during DKD has not been explored.

In this study, we delineate the spatio-temporal expression pattern of NUPR1 in DKD and mechanistically dissect its contribution to podocyte injury and MC. Most importantly, we provide the first evidence that pharmacological blockade of NUPR1 with the small-molecule inhibitor ZZW-115 not only prevents podocyte MC but also markedly halts the progression of DKD, unveiling a promising therapeutic avenue.

## Results

### NUPR1 was upregulated in podocytes in diabetic kidney disease mice and human patients

**Fig. 1:**
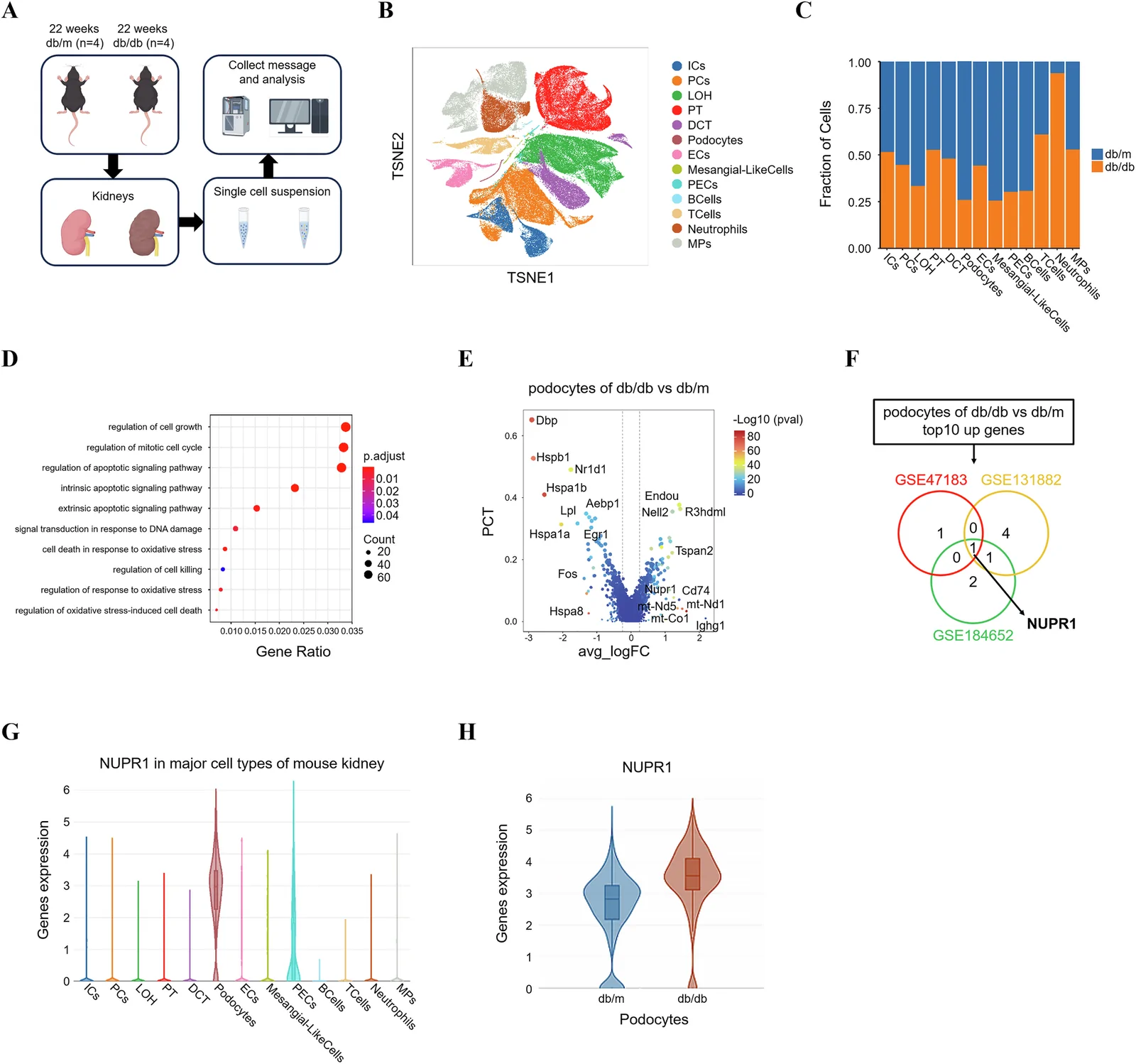
Single-cell RNA sequencing reveals NUPR1 involvement in podocyte injury in diabetic kidney disease. **A** Experimental workflow for single-cell RNA sequencing of kidneys from spontaneous type 2 diabetes db/db and db/m mice (*n* = 4 per group; created with Figdraw). **B** t-distributed stochastic neighbor embedding plot showing clustering of all kidney cells from db/db mice into 13 major cell subpopulations on the basis of characteristic markers. **C** Proportional map of each cell subpopulation in kidney tissue of db/m and db/db mice. **D** Gene ontology pathway enrichment analysis in podocytes. **E** Volcano plot of differentially expressed genes in podocytes. **F** Analytical workflow identifying the involvement of NUPR1 in podocyte injury in DKD. **G** Violin plot showing the distribution of NUPR1 expression in single-cell RNA sequencing data (*n* = 4 per group). **H** NUPR1 expression in podocytes from db/db mice kidney tissue compared to db/m controls, as shown by single-cell RNA sequencing (*n* = 4 per group). The data are presented as the means ± SDs from three independent experiments. *p < 0.05, **p < 0.01, ***p < 0.001; NS, not significant (Student’s t test).

To investigate the pathogenesis of DKD and identify potential therapeutic targets, we performed single-cell RNA sequencing on renal tissues obtained from 22-week-old db/m and db/db mice (*n* = 4 per group) (Fig. 1A). After standard data preprocessing and stringent quality control, all major renal cell types were successfully identified and annotated (Fig. 1B). Analysis of proportional changes in cell populations revealed significant alterations in podocytes and immune cells in DKD (Fig. 1C). Given the pivotal role of podocytes in DKD progression, we aimed to develop targeted therapeutic strategies focused on podocyte injury. We first investigated biological processes associated with podocytes. Gene Ontology analysis revealed significant enrichment in pathways related to cell injury, cell death, and the cell cycle (Fig. 1D). We then identified the top 10 upregulated genes specifically in podocytes from the db/db group (Fig. 1E). Cross-platform integration of overlapping transcriptional profiles from publicly available datasets (GSE47183, GSE131882, and GSE184652) consistently demonstrated significant upregulation of NUPR1 across multiple independent databases (Fig. 1F), suggesting its potential importance in the pathogenesis of DKD.

**Fig. 2:**
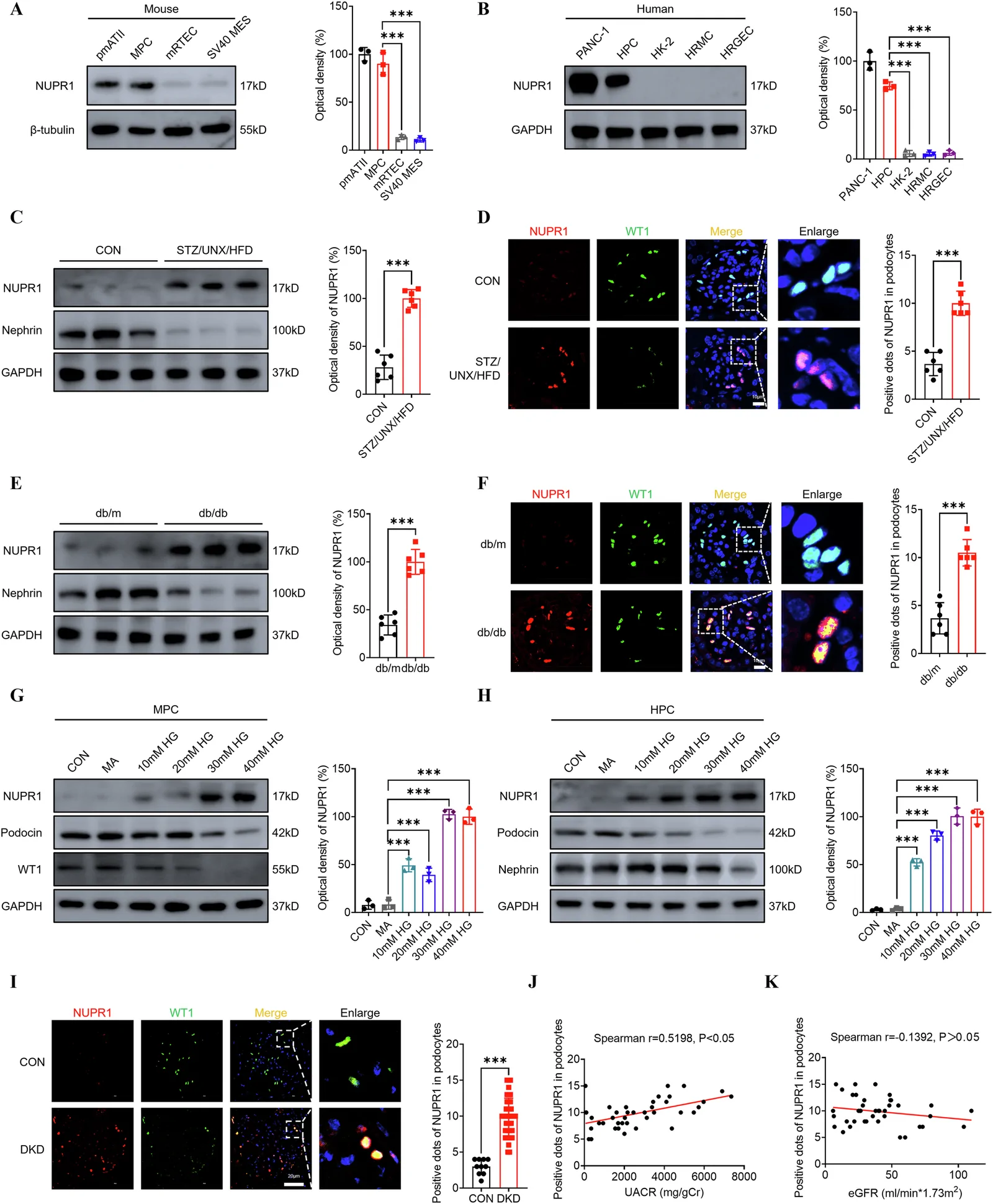
NUPR1 was upregulated in podocytes of diabetic kidney disease mice and patients. **A** Representative western blot images and quantitative analysis of NUPR1 protein levels in mouse renal cells (podocytes [MPC], renal tubular epithelial cells [mRTEC], and mesangial cells [SV40 MES]), primary mouse type II alveolar epithelial cells (pmATII) served as positive controls (*n* = 3 per group). **B** Representative western blot images and quantitative analysis of NUPR1 protein levels in human renal cells (podocytes [HPC], renal tubular epithelial cells [HK-2], mesangial cells [HRMC], and glomerular endothelial cells [HRGEC]), PANC-1 cells served as positive controls (*n* = 3 per group). **C** Representative western blot images and quantitative analysis of NUPR1 protein levels in the renal cortex of control mice and STZ/UNX/HFD-induced DKD mice (*n* = 6 per group). **D** Representative immunofluorescence staining of NUPR1 and WT1 in kidney sections from control mice and STZ/UNX/HFD DKD mice (*n* = 3 per group). Scale bars: 10 μm. **E** Representative western blot images and quantitative analysis of NUPR1 protein levels in the renal cortex of db/m and db/db mice (*n* = 3 per group). **F** Representative immunofluorescence images of NUPR1 and WT1 in kidney sections from db/m and db/db mice (*n* = 3 per group). Scale bars: 10 μm. **G** Representative western blot images and quantitative analysis of NUPR1 protein levels in mouse podocytes treated with high-glucose HG (10, 20, 30, and 40 mM) for 72 h, mannitol (MA) was used as an osmotic control (*n* = 3 per group). **H** Representative western blot images and quantitative analysis of NUPR1 protein levels in human podocytes treated with high-glucose HG (10, 20, 30, and 40 mM) for 72 h, MA was used as an osmotic control (*n* = 3 per group). **I** Representative immunofluorescence staining of NUPR1 and WT1 in kidney sections from the control group (*n* = 10) and a patient with DKD (*n* = 40). Scale bars: 20 μm. **J** Correlation between podocyte NUPR1 expression and the urinary albumin‒creatinine ratio (UACR) in all participants (*n* = 40). **K** Correlation between podocyte NUPR1 expression and the estimated glomerular filtration rate (eGFR) in all participants (*n* = 40). The data are presented as the means ± SDs from three independent experiments. *p < 0.05, **p < 0.01, ***p < 0.001; NS, not significant (Student’s *t* test).

To determine the role of NUPR1 in the kidney, we first assessed its cellular localization. Analysis of single-cell sequencing data revealed that NUPR1 is predominantly and selectively expressed in podocytes among diverse renal cell subtypes (Fig. 1G). We further evaluated NUPR1 expression under pathological conditions based on sequencing data to explore its potential involvement in podocyte injury in DKD (Fig. 1H). We extended this analysis to human and mouse renal parenchymal cells, including podocytes, tubular epithelial cells, mesangial cells, and endothelial cells. Notably, even under basal conditions, podocytes exhibited substantially higher NUPR1 expression compared with other renal cell types and positive control cells (Fig. 2A, B), underscoring their cell type-specific expression profile.

In the STZ/UNX/HFD-induced experimental DKD mice, renal cortical NUPR1 expression was significantly elevated relative to controls, as confirmed by western blot analysis (Fig. 2C). Immunofluorescence staining also further showed intensified NUPR1-specific signals localized specifically within podocytes of DKD mice (Fig. 2D), with minimal immunoreactivity in tubular epithelial cells (Fig. S1A), mesangial cells (Fig. S1B), and endothelial cells (Fig. S1C). Consistent results were observed in a spontaneous model of type 2 diabetes (Figs. 2E, F and S1D–F). In parallel in vitro experiments, exposure of mouse podocytes to (HG; 10, 20, 30, and 40 mmol/L) significantly induced NUPR1 expression (Fig. 2G). Importantly, analogous upregulation was observed in cultured human podocytes (Fig. 2H), supporting the translational relevance of our findings.

We further validated these results in clinical samples through immunofluorescence staining of renal biopsies from patients with DKD (*n* = 40). These analyses confirmed substantial upregulation of NUPR1 expression, which prominently colocalized with the podocyte nuclear marker WT1 (Fig. 2I). Correlation analysis revealed that NUPR1 immunostaining intensity in podocytes positively correlated with the urinary albumin to creatinine ratio (UACR) (Fig. 2J), a key clinical indicator of renal damage in DKD. However, no statistically significant correlation was observed with the estimated glomerular filtration rate (eGFR) (Fig. 2K). Additionally, we analyzed the correlation between NUPR1 expression and several baseline clinical characteristics, including age, height, body weight, disease duration (the clinical course following confirmed diabetes diagnosis), and glycated hemoglobin (Supplementary Table 1). None of these factors showed a significant correlation with NUPR1 expression, thereby ruling out potential confounding effects from these variables.

### NUPR1 aggravated podocyte injury and albuminuria in diabetic kidney disease mice

**Fig. 3:**
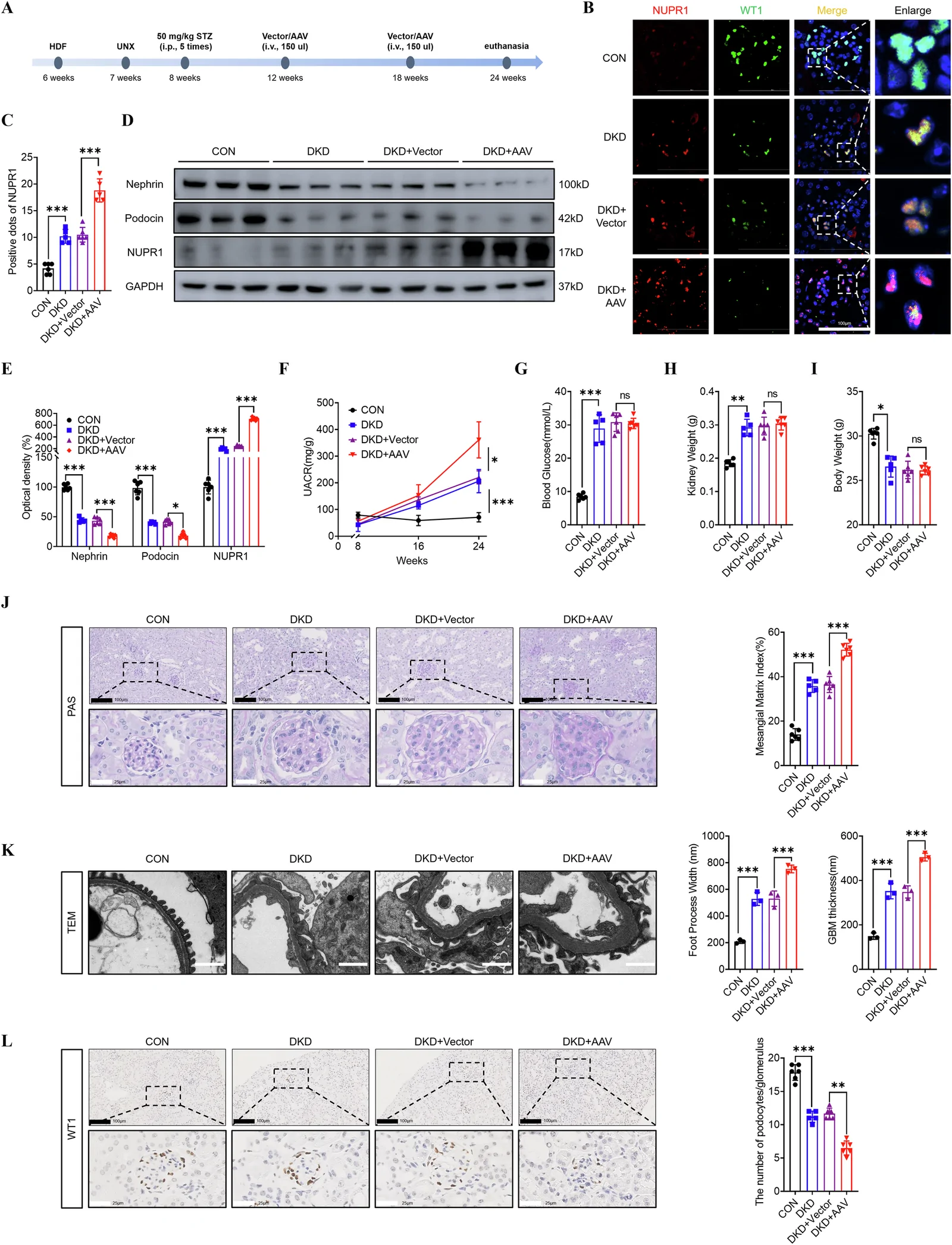
Overexpression of NUPR1 exacerbated podocyte injury and albuminuria in diabetic kidney disease mice. **A** Experimental design for generating NUPR1-overexpressing STZ/UNX/HFD DKD mice. **B** Representative images of NUPR1 and WT1 immunofluorescence staining in kidney sections from different groups of mice: CON, DKD, DKD+Vector, DKD + AAV. Scale bars: 100 μm. **C** Quantitative fluorescence analysis of NUPR1 in kidney sections from different groups of mice: CON (*n* = 6), DKD (*n* = 5, one mouse died due to an accidental event during the experiment), DKD+Vector (*n* = 6), DKD + AAV (*n* = 6). **D** Representative western blot images of NUPR1, Nephrin, and Podocin protein levels in renal cortex tissues from different groups of mice: CON, DKD, DKD+Vector, DKD + AAV. **E** Quantitative analysis of NUPR1, Nephrin, and Podocin protein levels in renal cortex tissues from different groups of mice: CON (*n* = 6), DKD (*n* = 5, one mouse died due to an accidental event during the experiment), DKD+Vector (*n* = 6), DKD + AAV (*n* = 6). **F** Urinary albumin‒creatinine ratio (UACR) in different groups of mice: CON (*n* = 6), DKD (*n* = 5, one mouse died due to an accidental event during the experiment), DKD+Vector (*n* = 6), DKD + AAV (*n* = 6). **G** Blood glucose levels in different groups of mice: CON (*n* = 6), DKD (*n* = 5, one mouse died due to an accidental event during the experiment), DKD+Vector (*n* = 6), DKD + AAV (*n* = 6). **H** Kidney weights in different groups of mice: CON (*n* = 6), DKD (*n* = 5, one mouse died due to an accidental event during the experiment), DKD+Vector (*n* = 6), DKD + AAV (*n* = 6). **I** Body weights of the different groups of mice: CON (*n* = 6), DKD (*n* = 5, one mouse died due to an accidental event during the experiment), DKD+Vector (*n* = 6), DKD + AAV (*n* = 6). **J** Representative periodic acid-Schiff (PAS)-stained images and quantitative analysis of glomerular morphological changes across the groups: CON (*n* = 6), DKD (*n* = 5, one mouse died due to an accidental event during the experiment), DKD+Vector (*n* = 6), DKD + AAV (*n* = 6). Black scale bars: 100 μm; white scale bars: 25 μm. **K** Representative transmission electron microscopy (TEM) images and quantitative analysis of the mean glomerular basement membrane (GBM) thickness and mean foot process width in different groups of mice (*n* = 3 per group): CON, DKD, DKD+Vector, DKD + AAV. Scale bars: 1 μm. **L** Representative immunohistochemical staining of WT1 in kidney sections from different groups of mice: CON (*n* = 6), DKD (*n* = 5, one mouse died due to an accidental event during the experiment), DKD+Vector (*n* = 6), DKD + AAV (*n* = 6). Black scale bars: 100 μm; white scale bars: 25 μm. The data are presented as the means ± SDs from three independent experiments. *p < 0.05, **p < 0.01, ***p < 0.001; NS, not significant (Student’s *t* test).

To investigate the functional role of NUPR1 in DKD, we employed a hydrodynamic-based gene delivery approach involving tail vein injection of an adeno-associated virus (AAV) vector encoding NUPR1 for overexpression in mice (Fig. 3A). Successful in vivo transfection and NUPR1 overexpression were confirmed by immunofluorescence staining (Fig. 3B, C) and western blot analysis of renal tissues (Fig. 3D, E), both of which revealed significantly elevated NUPR1 protein levels in the AAV-NUPR1-treated animals.

Under DKD conditions, mice overexpressing NUPR1 exhibited a significant increase in albuminuria compared with DKD control mice (Fig. 3F). Notably, NUPR1 overexpression did not affect blood glucose levels (Fig. 3G), kidney weight, or body weight (Fig. 3H, I), suggesting that its detrimental effects are not due to systemic metabolic alterations.

Histopathological assessment by periodic acid-Schiff staining showed increased glomerular mesangial matrix expansion and pathological abnormalities in NUPR1-overexpressing DKD mice (Fig. 3J). Ultrastructural analysis via transmission electron microscopy revealed severe pathological alterations, including thickening of the glomerular basement membrane and extensive effacement of podocyte foot processes (Fig. 3K). Immunohistochemical staining for Wilms’ tumor 1 (WT1), a podocyte nuclear marker, indicated a marked reduction in podocyte density in NUPR1-overexpressing mice, suggesting accelerated podocyte loss (Fig. 3L). Western blot analysis confirmed these findings, revealing downregulation of podocyte marker proteins, including nephrin and podocin, thereby confirming that NUPR1 induced podocyte injury (Fig. 3D, E).

### NUPR1 aggravated HG-induced podocyte injury

Given that HG is a well-established inducer of podocyte damage in vitro [5], we treated conditionally immortalized mouse podocyte cells (MPC) with 30 mmol/L glucose for 72 h to mimic diabetic conditions. This exposure to HG resulted in a significant downregulation of podocyte differentiation markers, such as synaptopodin and nephrin, accompanied by a concurrent upregulation of NUPR1 expression. These changes were confirmed by both quantitative reverse transcription polymerase chain reaction (RT-qPCR) (Fig. S2A) and western blot analyses (Fig. S2B).

To directly assess the functional involvement of NUPR1 in this process, we established an in vitro NUPR1 overexpression model using lentivirus-mediated gene delivery into podocytes (Fig. S2C, D). Under HG conditions, NUPR1 overexpression resulted in a further reduction in the expression of podocyte-specific proteins synaptopodin and podocin, indicating a synergistic detrimental effect (Fig. S2E, F). Conversely, we knocked down NUPR1 expression in vitro using small interfering RNA (siRNA). Knockdown efficiency was confirmed at both the mRNA and protein levels via RT-qPCR (Fig. S2G) and western blot (Fig. S2H). Among the three designed siRNAs, the knockdown efficiencies of siNUPR1-1, siNUPR1-2, and siNUPR1-3 were approximately 87%, 67%, and 74%, respectively. Of these, siNUPR1-1 exhibited the highest knockdown efficiency and was therefore selected for all subsequent experiments (Fig. S2I). Notably, NUPR1 silencing effectively restored the expression of synaptopodin and podocin that had been downregulated by HG treatment (Fig. S2J. K). Together, these genetic manipulations establish that NUPR1 plays a causal role in exacerbating HG-induced podocyte injury.

### NUPR1 regulated the cell cycle in podocyte injury of diabetic kidney disease

**Fig. 4:**
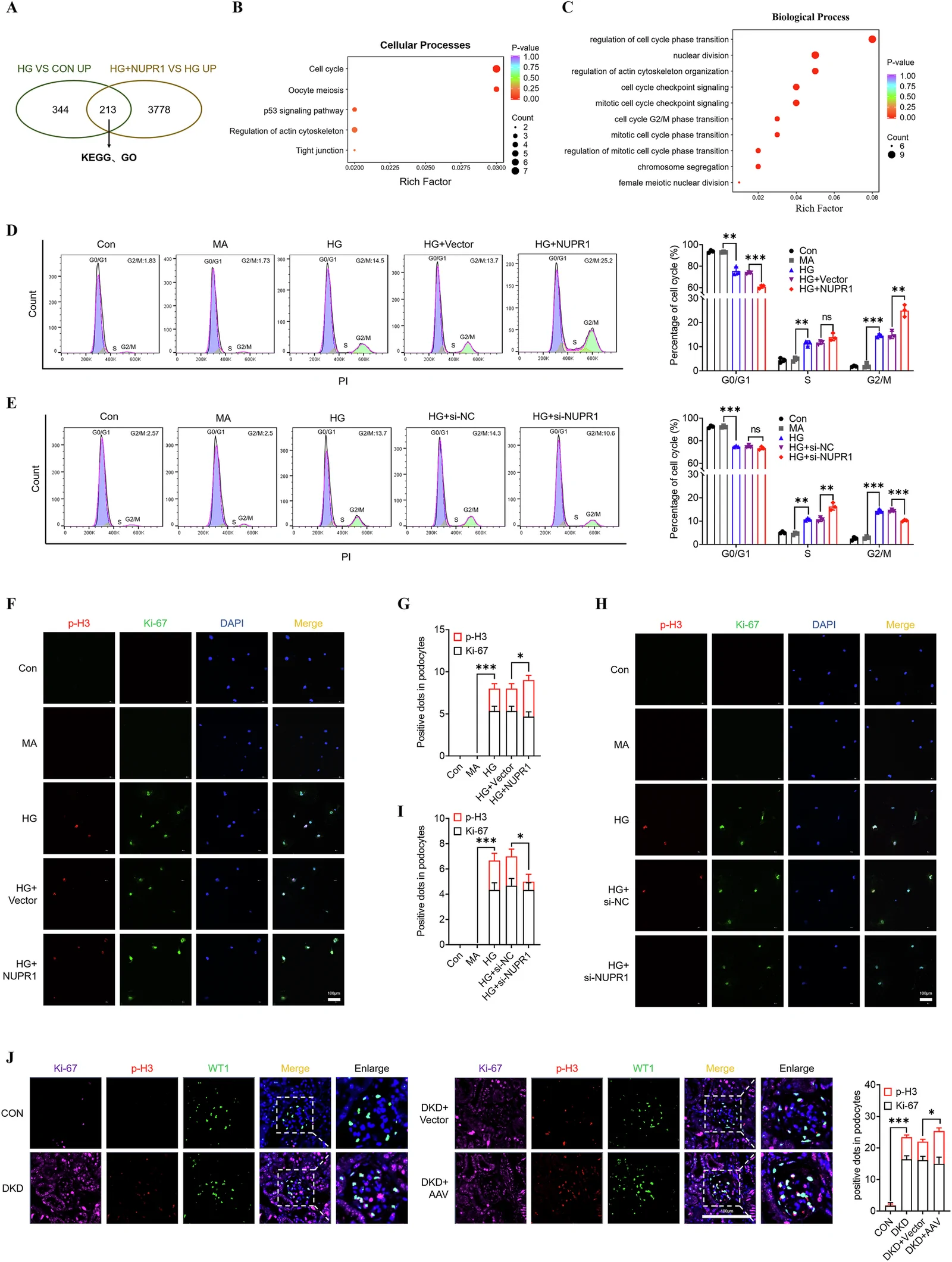
NUPR1 regulated the cell cycle in podocyte injury of diabetic kidney disease. **A** Venn diagram showing overlapping genes. **B** Gene ontology pathway enrichment analysis. **C** Kyoto encyclopedia of genes and genomes enrichment analysis of biological pathways. **D** Flow cytometry analysis and quantitative assessment of the cell cycle distribution in different podocyte groups (*n* = 3 per group): Con, MA, HG, HG+Vector, HG + NUPR1. **E** Flow cytometry analysis and quantitative assessment of the cell cycle distribution in different groups of podocytes (*n* = 3 per group): Con, MA, HG, HG+si-NC, HG+si-NUPR1. **F** Representative immunofluorescence staining for p-H3 and Ki-67 in different podocyte groups: Con, MA, HG, HG+Vector, HG + NUPR1. Scale bars: 100 μm. **G** Quantitative analysis of representative immunofluorescence staining for p-H3 and Ki-67 in different podocyte groups (*n* = 3 per group): Con, MA, HG, HG+Vector, HG + NUPR1. **H** Representative immunofluorescence staining for p-H3 and Ki-67 in different podocyte groups: Con, MA, HG, HG+si-NC, HG+si-NUPR1. Scale bars: 100 μm. **I** Quantitative analysis of representative immunofluorescence staining for p-H3 and Ki-67 in different podocyte groups (*n* = 3 per group): Con, MA, HG, HG+si-NC, HG+si-NUPR1. **J** Representative images of p-H3, Ki-67, and WT1 immunofluorescence staining and quantitative analysis of kidney sections from different groups of mice: CON (*n* = 6), DKD (*n* = 5, one mouse died due to an accidental event during the experiment), DKD+Vector (*n* = 6), DKD + AAV (*n* = 6). Scale bars: 100 μm. The data are presented as the means ± SDs from three independent experiments. *p < 0.05, **p < 0.01, ***p < 0.001; NS, not significant (Student’s t test).

To elucidate the biological processes and pathways through which NUPR1 mediates podocyte injury and to consider its role as a transcriptional regulator, we conducted comprehensive transcriptome sequencing of podocytes under four experimental conditions: control (CON), high glucose (HG), HG with NUPR1 overexpression (HG + OE-NUPR1), and HG with NUPR1 knockdown (HG+siNUPR1). Employing a stringent analytical approach, we identified two overlapping gene sets: 213 genes commonly upregulated in both the HG versus CON and HG + OE-NUPR1 versus HG comparisons (Fig. 4A), and 41 genes upregulated in the HG versus CON comparisons but downregulated in the HG+siNUPR1 versus HG comparisons (Fig. S3A). For downstream enrichment analyses, we prioritized the larger set of 213 genes to ensure statistical robustness. Gene Ontology and Kyoto Encyclopedia of Genes and Genomes analyses revealed that these genes were significantly enriched in biological processes and pathways associated with cell cycle regulation, particularly mitotic division (Fig. 4B, C).

We next evaluated the dynamics of the podocyte cell cycle under these experimental conditions. Flow cytometric analysis revealed that HG stimulation induced a significant number of podocytes to re-enter the cell cycle, advancing into the S and G2/M phases (Fig. 4D). The overexpression of NUPR1 further amplified this effect, leading to a marked accumulation of podocytes in the G2/M phase (Fig. 4D). In contrast, the knockdown of NUPR1 via siRNA under HG conditions effectively inhibited the entry of podocytes into the G2/M phase (Fig. 4E). In line with these findings, elevated levels of phosphorylated histone H3 (p-H3), a specific marker of mitotic entry, indicated that NUPR1 promotes aberrant cell cycle re-entry in these terminally differentiated cells (Fig. 4F, G). Similarly, the knockdown of NUPR1 under HG stimulation suppressed this phenomenon (Fig. 4H, I). These in vitro findings were corroborated by in vivo studies, which confirmed increased expression of both p-H3 and Ki-67 in podocytes of NUPR1-overexpressing DKD mice through immunofluorescence staining (Fig. 4J).

### NUPR1 exacerbated podocyte injury and apoptosis by inducing mitotic catastrophe

**Fig. 5:**
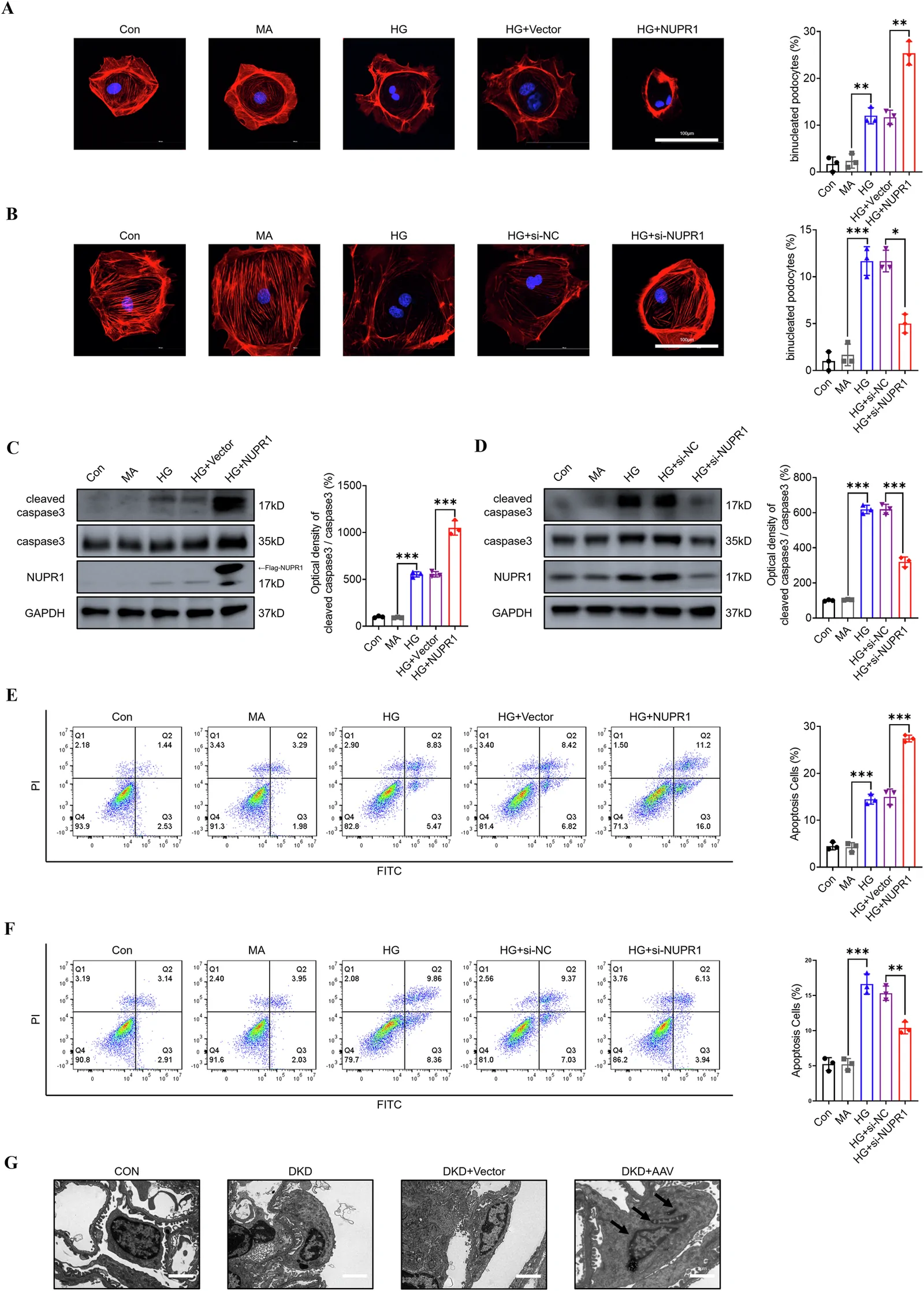
NUPR1 exacerbated podocyte injury and apoptosis by inducing mitotic catastrophe. **A** Representative images of the actin cytoskeleton structure and quantitative analysis of binucleated podocytes across the groups (*n* = 3 per group): Con, MA, HG, HG+Vector, HG + NUPR1. Scale bars: 100 μm. **B** Representative images of the actin cytoskeleton structure and quantitative analysis of binucleated podocytes across the groups (*n* = 3 per group): Con, MA, HG, HG+si-NC, HG+si-NUPR1. Scale bars: 100 μm. **C** Representative western blot images and quantitative analysis of cleaved caspase-3, caspase-3, and NUPR1 protein levels in the different podocyte groups (*n* = 3 per group): Con, MA, HG, HG+Vector, HG + NUPR1. **D** Representative western blot images and quantitative analysis of cleaved caspase-3, caspase-3, and NUPR1 protein expression in the different podocyte groups (*n* = 3 per group): Con, MA, HG, HG+si-NC, HG+si-NUPR1. **E** Flow cytometry analysis and quantitative evaluation of apoptosis in different podocyte groups (*n* = 3 per group): Con, MA, HG, HG+Vector, HG + NUPR1. **F** Flow cytometry analysis and quantitative evaluation of apoptosis in different podocyte groups (*n* = 3 per group): Con, MA, HG, HG+si-NC, HG+si-NUPR1. **G** Representative transmission electron microscopy (TEM) images showing ultrastructural changes in podocytes across different groups of mice: CON, DKD, DKD+Vector, DKD + AAV. Scale bars: 2 μm. The data are presented as the means ± SDs from three independent experiments. *p < 0.05, **p < 0.01, ***p < 0.001; NS, not significant (Student’s *t* test).

In podocytes, aberrant cell cycle re-entry can lead to MC, a form of cell death triggered by defective mitosis, morphologically characterized by binucleation or multinucleation. This process ultimately contributes to podocyte loss and renal functional decline. Under HG conditions, approximately 10% of podocytes displayed binucleation, a morphological indicator of MC reflecting unsuccessful mitosis and tetraploidization (Fig. 5A). Overexpression of NUPR1 significantly increased the proportion of binucleated podocytes, suggesting aggravation of MC (Fig. 5A). In contrast, siRNA-mediated knockdown of NUPR1 under HG stimulation reduced binucleation (Fig. 5B).

We further evaluated podocyte apoptosis under these conditions. Western blot analysis of cleaved caspase-3 demonstrated that NUPR1 overexpression markedly increased its level in HG-stimulated podocytes (Fig. 5C), whereas NUPR1 knockdown under HG conditions effectively decreased cleaved caspase-3 expression (Fig. 5D). Flow cytometry with Annexin V staining further confirmed these findings: NUPR1 overexpression promoted apoptosis (Fig. 5E), while NUPR1 knockdown reduced apoptotic rates (Fig. 5F). Together, these results indicate that suppression of NUPR1 alleviates both MC and subsequent apoptosis.

These in vitro findings were supported by in vivo evidence. Ultrastructural analysis of podocytes from DKD mice revealed binucleation, nuclear pyknosis, and detachment from the glomerular basement membrane (Fig. 5G). These pathological features were more severe in NUPR1-overexpressing DKD mice (Fig. 5G), further confirming the role of NUPR1 in promoting MC and podocyte injury.

### NUPR1 promoted mitotic catastrophe in podocytes by upregulating Cyclin B2 expression

**Fig. 6:**
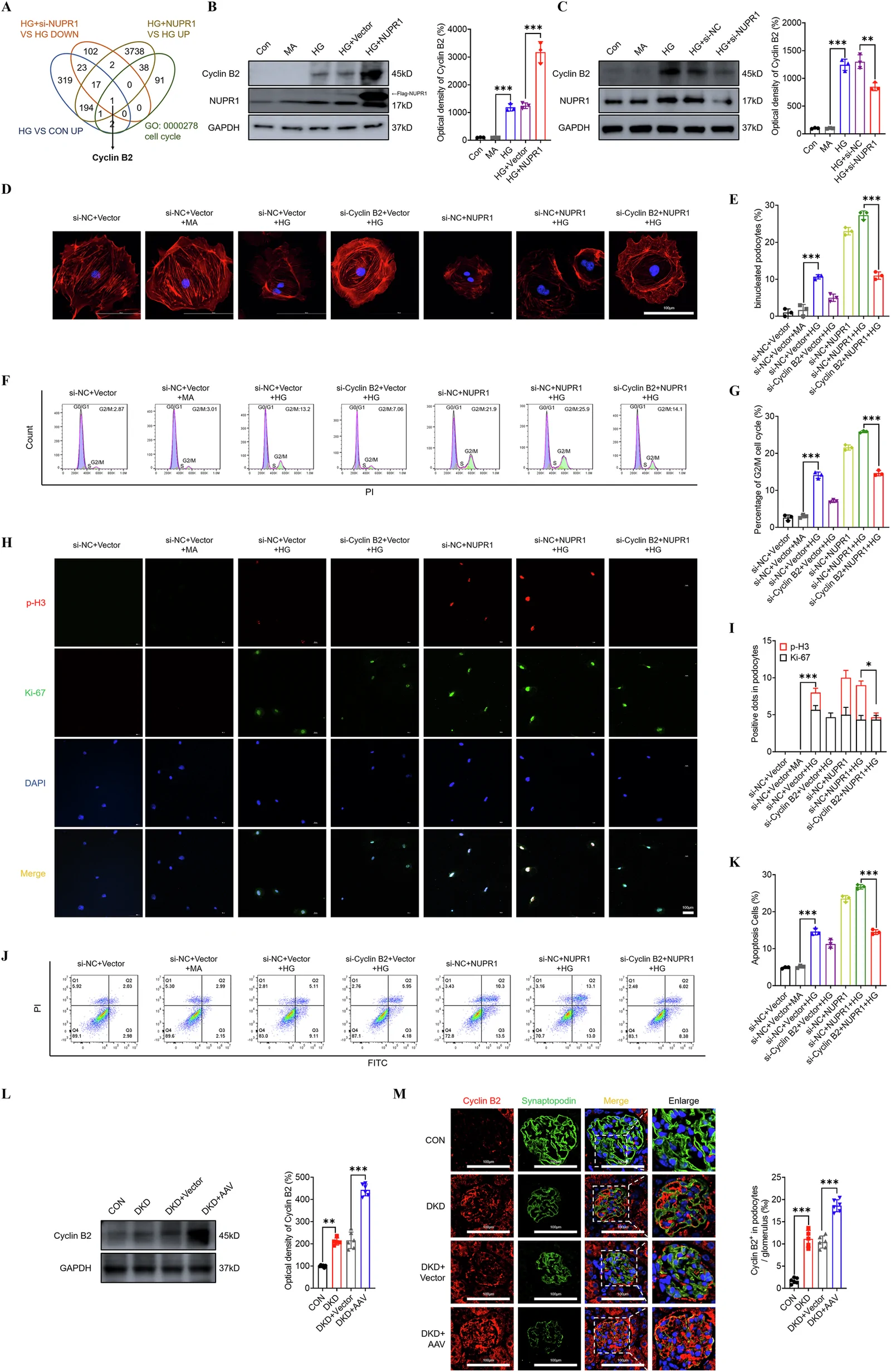
NUPR1 regulated podocyte mitotic catastrophe by inducing Cyclin B2 expression in podocytes. **A** Venn diagram showing overlapping genes. **B** Representative western blot images and quantitative analysis of Cyclin B2 protein levels in different podocyte groups (*n* = 3 per group): Con, MA, HG, HG+Vector, HG + NUPR1. **C** Representative western blot images and quantitative analysis of Cyclin B2 protein levels in different podocyte groups (*n* = 3 per group): Con, MA, HG, HG+si-NC, HG+si-NUPR1. **D** Representative images of the actin cytoskeleton structure across the groups: si-NC+Vector, si-NC+Vector+MA, si-NC+Vector+HG, si-Cyclin B2+Vector+HG, si-NC + NUPR1, si-NC + NUPR1 + HG, si-Cyclin B2 + NUPR1 + HG. Scale bars: 100 μm. **E** Quantitative analysis of binucleated podocytes across the groups (*n* = 3 per group): si-NC+Vector, si-NC+Vector+MA, si-NC+Vector+HG, si-Cyclin B2+Vector+HG, si-NC + NUPR1, si-NC + NUPR1 + HG, si-Cyclin B2 + NUPR1 + HG. **F** Flow cytometry analysis of the cell cycle distribution in different podocyte groups: si-NC+Vector, si-NC+Vector+MA, si-NC+Vector+HG, si-Cyclin B2+Vector+HG, si-NC + NUPR1, si-NC + NUPR1 + HG, si-Cyclin B2 + NUPR1 + HG. **G** Quantitative assessment of the cell cycle distribution in different podocyte groups (*n* = 3 per group): si-NC+Vector, si-NC+Vector+MA, si-NC+Vector+HG, si-Cyclin B2+Vector+HG, si-NC + NUPR1, si-NC + NUPR1 + HG, si-Cyclin B2 + NUPR1 + HG. **H** Representative immunofluorescence staining for p-H3 and Ki-67 in different podocyte groups: si-NC+Vector, si-NC+Vector+MA, si-NC+Vector+HG, si-Cyclin B2+Vector+HG, si-NC + NUPR1, si-NC + NUPR1 + HG, si-Cyclin B2 + NUPR1 + HG. Scale bars: 100 μm. **I** Quantitative analysis of representative immunofluorescence staining for p-H3 and Ki-67 in different podocyte groups (*n* = 3 per group): si-NC+Vector, si-NC+Vector+MA, si-NC+Vector+HG, si-Cyclin B2+Vector+HG, si-NC + NUPR1, si-NC + NUPR1 + HG, si-Cyclin B2 + NUPR1 + HG. **J** Flow cytometry analysis of apoptosis in different podocyte groups: si-NC+Vector, si-NC+Vector+MA, si-NC+Vector+HG, si-Cyclin B2+Vector+HG, si-NC + NUPR1, si-NC + NUPR1 + HG, si-Cyclin B2 + NUPR1 + HG. **K** Quantitative evaluation of apoptosis in different podocyte groups (*n* = 3 per group): si-NC+Vector, si-NC+Vector+MA, si-NC+Vector+HG, si-Cyclin B2+Vector+HG, si-NC + NUPR1, si-NC + NUPR1 + HG, si-Cyclin B2 + NUPR1 + HG. **L** Representative western blot images and quantitative analysis of Cyclin B2 protein levels in renal cortex tissues from different groups of mice: CON (*n* = 6), DKD (*n* = 5, one mouse died due to an accidental event during the experiment), DKD+Vector (*n* = 6), DKD + AAV (*n* = 6). **M** Representative images of Cyclin B2 and Synaptopodin immunofluorescence staining and quantitative analysis of kidney sections from different groups of mice: CON (*n* = 6), DKD (*n* = 5, one mouse died due to an accidental event during the experiment), DKD+Vector (*n* = 6), DKD + AAV (*n* = 6). Scale bars: 100 μm. The data are presented as the means ± SDs from three independent experiments. *p < 0.05, **p < 0.01, ***p < 0.001; NS, not significant (Student’s *t* test).

To identify downstream effectors through which NUPR1 mediates MC, we conducted a multi-step bioinformatics intersection analysis. Genes overlapping from four datasets were identified: (1) those upregulated under HG compared to CON conditions; (2) genes upregulated in HG-treated podocytes overexpressing NUPR1 (HG + OE-NUPR1) compared to HG alone; (3) genes downregulated following NUPR1 knockdown (HG+siNUPR1) compared to HG; and (4) genes associated with cell cycle-related pathways. This integrative approach identified Cyclin B2 as a key candidate, which is upregulated under HG and potentially regulated by NUPR1 (Fig. 6A). Independent validation via RT-qPCR (Fig. S4A) and western blotting (Fig. S4B) confirmed the HG-induced upregulation of Cyclin B2. Furthermore, we examined Cyclin B2 expression under HG conditions following either NUPR1 overexpression (Fig. 6B) or knockdown (Fig. 6C). These results suggest that Cyclin B2 may act as a downstream target of NUPR1 in the regulation of podocyte MC.

Functionally, silencing Cyclin B2 with a specific siRNA (Fig. S4C–F) alleviated the detrimental effects induced by NUPR1 overexpression in HG-treated podocytes. This was demonstrated by improved preservation of actin cytoskeleton architecture, a reduction in binucleated cells with abnormal mitotic spindles (Fig. 6D, E), a decreased proportion of cells in the G2/M phase (Fig. 6F–I), and reduced apoptosis (Fig. 6J, K). Conversely, overexpression of Cyclin B2 (Fig. S4G, H) in podocytes was sufficient to promote abnormal cell cycle progression (Fig S5A–D), exacerbate features of MC (Fig. S5E, F), and increase apoptosis under HG conditions (Fig. S5G, H). In vitro analyses, including western blot (Fig. 6L) and immunofluorescence (Fig. 6M), showed that overexpression of NUPR1 upregulated Cyclin B2 expression in podocytes. These findings suggest that Cyclin B2 may act as a potential downstream target through which NUPR1 regulates MC in podocytes.

### The NUPR1/BRD2 complex regulated Cyclin B2 transcription

**Fig. 7:**
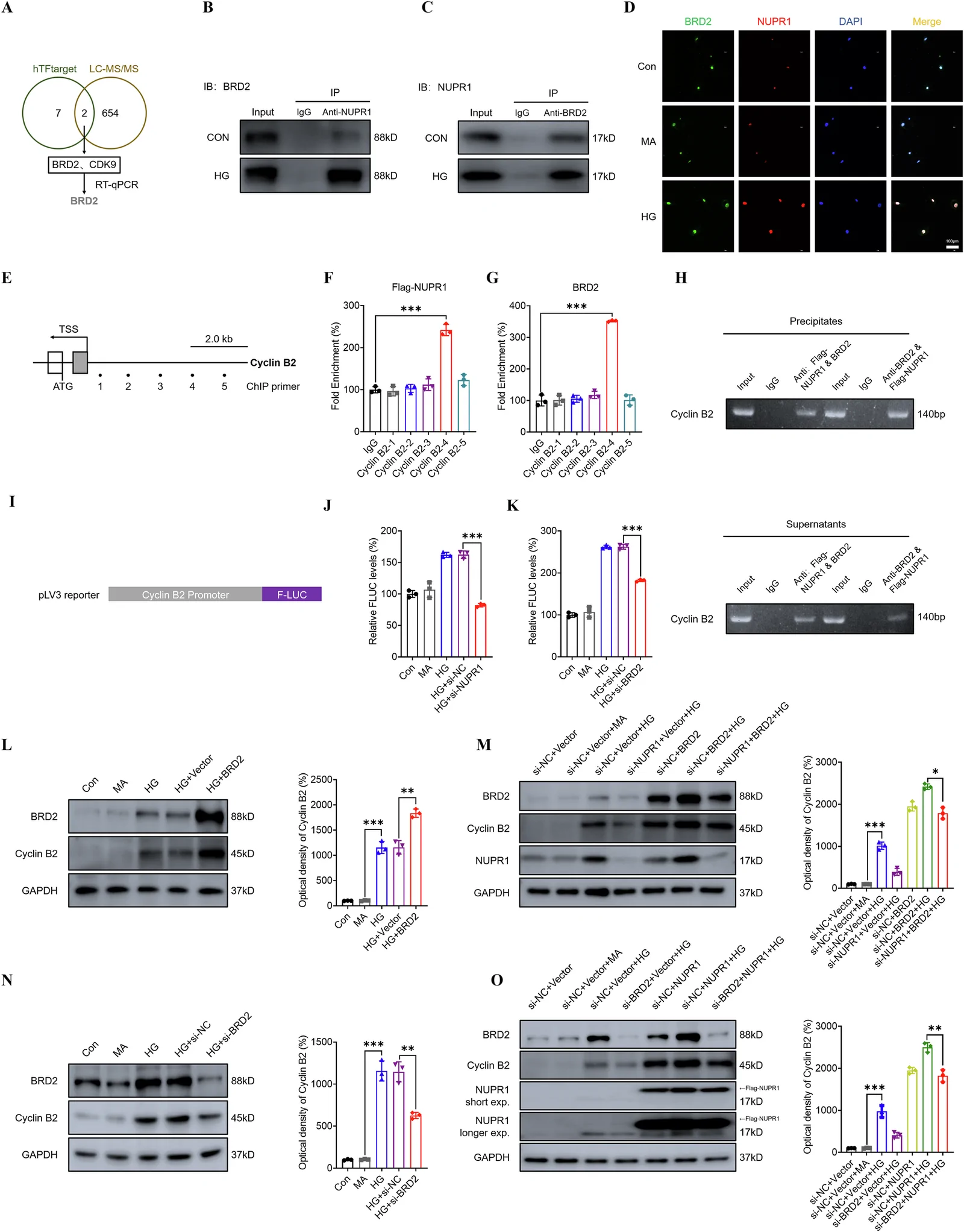
The NUPR1/BRD2 complex regulated Cyclin B2 transcription. **A** Venn diagram showing overlapping genes. **B** Co-immunoprecipitation assay of NUPR1 and BRD2 interaction, group: Input, IP (IgG), IP (Anti-NUPR1). **C** Co-immunoprecipitation assay of NUPR1 and BRD2 interaction, group: Input, IP (IgG), IP (Anti-BRD2). **D** Representative images of immunofluorescence staining for BRD2 and NUPR1 in different podocyte groups: Con, MA, HG. Scale bars: 100 μm. **E** Schematic representation of primer design for chromatin immunoprecipitation (ChIP)‒qPCR analysis. The TSS indicates the transcription start site of Cyclin B2, and the dots represent primer-binding positions. **F** ChIP‒qPCR analysis of NUPR1 binding to the Cyclin B2 promoter region (*n* = 3 per group): IgG, Cyclin B2-1, Cyclin B2-2, Cyclin B2-3, Cyclin B2-4, Cyclin B2-5. **G** ChIP‒qPCR analysis of BRD2 binding to the Cyclin B2 promoter region (*n* = 3 per group): IgG, Cyclin B2-1, Cyclin B2-2, Cyclin B2-3, Cyclin B2-4, Cyclin B2-5. **H** Sequential ChIP analysis of NUPR1 and BRD2 binding to the Cyclin B2 promoter. **I** Schematic representation of the pLV3-Cyclin B2 reporter. **J** Relative luciferase activity was used to assess Cyclin B2 transcriptional activity in different podocyte groups (*n* = 3 per group): Con, MA, HG, HG+si-NC, HG+si-NUPR1. **K** Relative luciferase activity was used to evaluate Cyclin B2 transcriptional regulation in different podocyte groups (*n* = 3 per group): Con, MA, HG, HG+si-NC, HG+si-BRD2. **L** Representative western blot images and quantitative analysis of Cyclin B2 protein levels in the different podocyte groups (*n* = 3 per group): Con, MA, HG, HG+Vector, HG + BRD2. **M** Representative western blot images and quantitative analysis of Cyclin B2 protein levels in the different podocyte groups (*n* = 3 per group): si-NC+Vector, si-NC+Vector+MA, si-NC+Vector+HG, si-NUPR1+Vector+HG, si-NC + BRD2, si-NC + BRD2 + HG, si-NUPR1 + BRD2 + HG. **N** Representative western blot images and quantitative analysis of Cyclin B2 protein levels in the different podocyte groups (*n* = 3 per group): Con, MA, HG, HG+si-NC, HG+si-BRD2. **O** Representative western blot images and quantitative analysis of Cyclin B2 protein levels in the different podocyte groups (*n* = 3 per group): si-NC+Vector, si-NC+Vector+MA, si-NC+Vector+HG, si-BRD2+Vector+HG, si-NC + NUPR1, si-NC + NUPR1 + HG, si-BRD2 + NUPR1 + HG. The data are presented as the means ± SDs from three independent experiments. *p < 0.05, **p < 0.01, ***p < 0.001; NS, not significant (Student’s *t* test).

Next, we explored the mechanism by which NUPR1 regulates Cyclin B2 expression. Since NUPR1 lacks a DNA-binding domain, it likely relies on interactions with other DNA-binding transcription factors to modulate transcription. Using the online hTFtarget database, we predicted potential transcriptional regulators of the Cyclin B2 promoter. We also compiled a list of 656 proteins identified as potential NUPR1-interacting partners through co-immunoprecipitation combined with mass spectrometry [19]. Intersection of these two datasets yielded two high-confidence candidate proteins: CDK9 and BRD2 (Fig. 7A). RT-qPCR analysis revealed that treatment with HG significantly increased the expression of BRD2 mRNA, but not that of CDK9, guiding our subsequent investigations to focus on BRD2 (Fig. 7A).

We employed various methods to validate the physical interaction between NUPR1 and BRD2. Immunofluorescence staining revealed significant nuclear co-localization of NUPR1 and BRD2 in podocytes. This interaction was further confirmed by co-immunoprecipitation assays, which demonstrated reciprocal binding between NUPR1 and BRD2. This binding was intensified under HG conditions, as evidenced by Fig. 7B, C. These findings were consistent with the immunofluorescence results (Fig. 7D).

To determine whether the NUPR1/BRD2 complex binds directly to the Cyclin B2 promoter, we performed chromatin immunoprecipitation (ChIP) assays. The results indicated significant enrichment of both NUPR1 and BRD2 proteins near the transcription start site of Cyclin B2 (Fig. 7E–G). A sequential ChIP-re-ChIP experiment, in which chromatin was first immunoprecipitated with an anti-NUPR1 antibody and then re-immunoprecipitated with an anti-BRD2 antibody, confirmed that NUPR1 and BRD2 coexist within the same protein complex bound to the Cyclin B2 promoter (Fig. 7H). The ChIP assay revealed that NUPR1 recruits BRD2 to the Cyclin B2 promoter region spanning +1300 to +1500 bp relative to the transcription start site. Furthermore, dual-luciferase reporter assays demonstrated that knockdown of either NUPR1 or BRD2 significantly reduced Cyclin B2 promoter activity (Fig. 7I–K). Finally, we confirmed that both NUPR1 and BRD2 were required for fully inducing Cyclin B2 expression at the mRNA and protein levels. (Fig. 7L–O).

### The NUPR1 inhibitor ZZW-115 exerted protective effects on podocytes both in vitro and in vivo

Currently, two pharmacological inhibitors of NUPR1 are available: trifluoperazine and ZZW-115. Trifluoperazine, an antipsychotic agent with known NUPR1-inhibitory activity, has limited clinical applicability due to its significant neuropsychiatric side effects. In contrast, ZZW-115, a recently developed compound, exhibits high affinity for NUPR1 and has not been associated with overt psychiatric side effects in preclinical models.

We first determined that ZZW-115 inhibited the viability of MPC with an IC₅₀ of 6.29 μM (Fig. S6A). Subsequently, a dose–response experiment was performed with ZZW-115 at concentrations of 0, 1, 2, 4, 6, and 8 μM. The results showed that ZZW-115 attenuated the nuclear import of NUPR1 in a concentration-dependent manner at 1 μM and 2 μM, with complete abrogation of nuclear translocation observed at 4 μM (Fig. S6B); this concentration was therefore selected for all subsequent experiments. In cultured podocytes under HG conditions, 4 μM ZZW-115 completely abrogated HG-induced nuclear translocation of NUPR1 (Fig. S7A).

After characterizing the inhibitory effect of ZZW-115, we next investigated its mechanism of action in vitro. Co-immunoprecipitation assays revealed that ZZW-115 disrupts the interaction between NUPR1 and BRD2 (Fig. S7B), and Western blot analysis confirmed the subsequent downregulation of Cyclin B2 (Fig. S7C). To assess the functional consequences, we examined cell cycle progression and apoptosis. ZZW-115 partially alleviated HG-induced G2/M phase arrest (Fig. S7D-F) and significantly reduced the proportion of binucleated podocytes, indicating protection against MC (Fig. S7G). Notably, ZZW-115 markedly reduced apoptosis, as evidenced by decreased levels of cleaved caspase-3 (Fig. S7H) and a lower proportion of Annexin V-positive cells (Fig. S7I). Collectively, these effects contributed to the restoration of podocyte differentiation markers nephrin and podocin suppressed by HG (Fig. S7J).

**Fig. 8:**
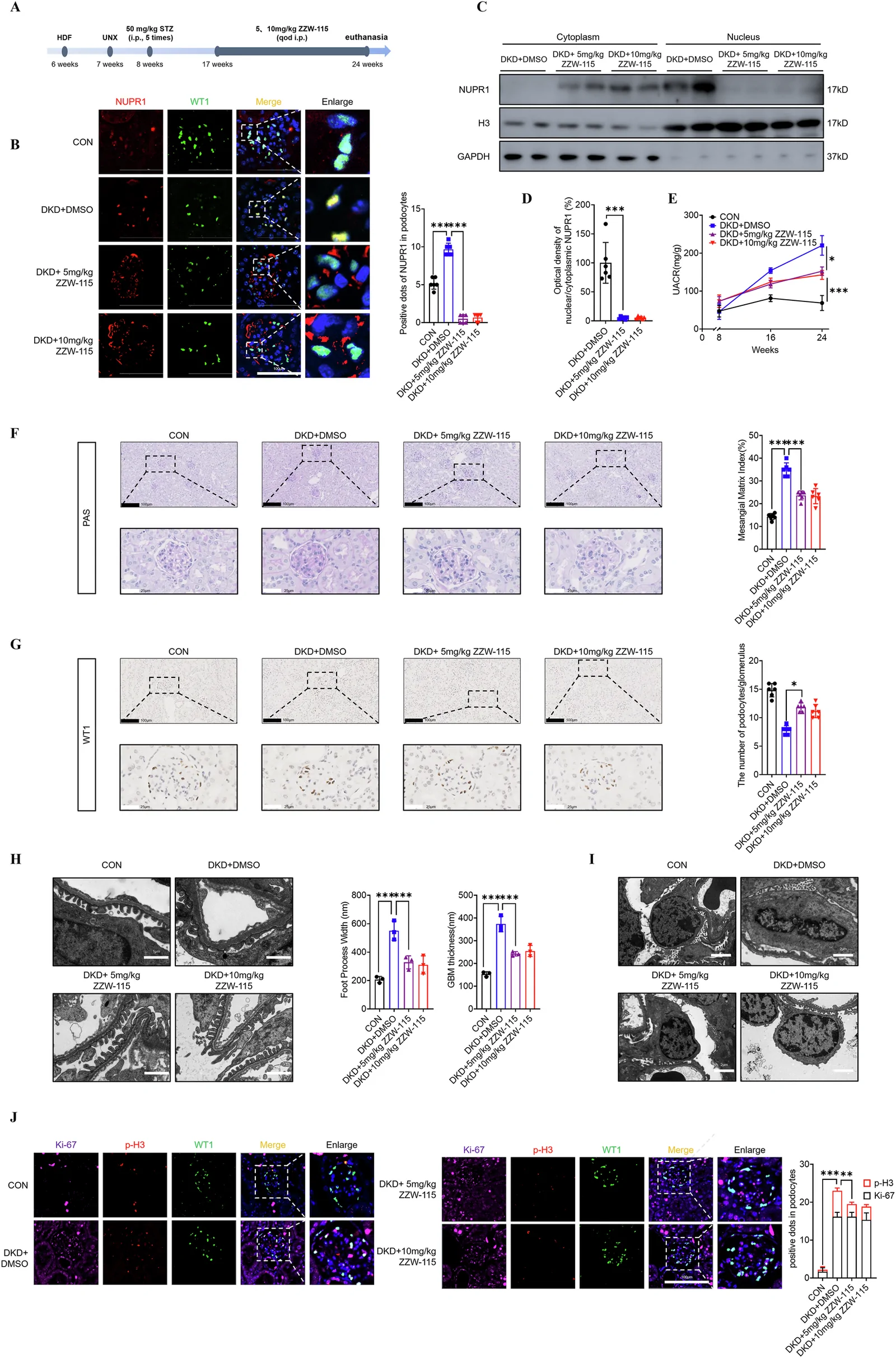
ZZW-115 alleviated podocyte injury and delayed diabetic kidney disease progression. **A** Experimental design for ZZW-115 treatment of STZ/UNX/HFD DKD mice. **B** Representative images of NUPR1 and WT1 immunofluorescence staining and quantitative analysis in kidney sections from different groups of mice (*n* = 6 per group): CON, DKD + DMSO, DKD + 5 mg/kg ZZW-115, DKD + 10 mg/kg ZZW-115. Scale bars: 100 μm. **C** Representative western blot images of NUPR1 protein levels in the nuclear and cytoplasmic fractions from different groups of mice: DKD + DMSO, DKD + 5 mg/kg ZZW-115, DKD + 10 mg/kg ZZW-115. **D** Quantitative analysis of the nuclear/cytoplasmic ratio of NUPR1 expression in the nuclear-cytoplasmic fractionation assay from different groups of mice (*n* = 6 per group): DKD + DMSO, DKD + 5 mg/kg ZZW-115, DKD + 10 mg/kg ZZW-115. **E** Urinary albumin‒creatinine ratio (UACR) in different groups of mice (*n* = 6 per group): CON, DKD + DMSO, DKD + 5 mg/kg ZZW-115, DKD + 10 mg/kg ZZW-115. **F** Representative periodic acid–schiff stained images and quantitative analysis of glomerular morphological changes across the groups (*n* = 6 per group): CON, DKD + DMSO, DKD + 5 mg/kg ZZW-115, DKD + 10 mg/kg ZZW-115. Black scale bars: 100 μm; white scale bars: 25 μm. **G** Representative immunohistochemical staining of WT1 in kidney sections from different groups of mice (*n* = 6 per group): CON, DKD + DMSO, DKD + 5 mg/kg ZZW-115, DKD + 10 mg/kg ZZW-115. Black scale bars: 100 μm; white scale bars: 25 μm. **H** Representative transmission electron microscopy (TEM) images and quantitative analysis of the mean glomerular basement membrane (GBM) thickness and mean foot process width in different groups of mice (*n* = 3 per group): CON, DKD + DMSO, DKD + 5 mg/kg ZZW-115, DKD + 10 mg/kg ZZW-115. Scale bars: 1 μm. **I** Representative TEM images showing ultrastructural changes in podocytes across different groups of mice: CON, DKD + DMSO, DKD + 5 mg/kg ZZW-115, DKD + 10 mg/kg ZZW-115. Scale bars: 2 μm. **J** Representative images of p-H3, Ki-67, and WT1 immunofluorescence staining and quantitative analysis of kidney sections from different groups of mice (*n* = 6 per group): CON, DKD + DMSO, DKD + 5 mg/kg ZZW-115, DKD + 10 mg/kg ZZW-115. Scale bars: 100 μm. The data are presented as the means ± SDs from three independent experiments. *p < 0.05, **p < 0.01, ***p < 0.001; NS, not significant (Student’s t test).

In vivo, we evaluated the therapeutic potential of ZZW-115 in DKD mice. Guided by prior dose-response studies [19, 20], mice received 5 or 10 mg/kg ZZW-115 (Fig. 8A). Both doses efficiently blocked NUPR1 nuclear translocation in renal tissue (Fig. 8B–D). To assess safety, serum biochemistry was analyzed, neither dose altered alanine aminotransferase (ALT), aspartate aminotransferase (AST), or lactate dehydrogenase (LDH) levels (Fig. S8A–C), indicating an absence of hepatotoxicity.

ZZW-115 markedly reduced albuminuria in DKD mice (Fig. 8E) without affecting blood glucose (Fig. S8D), kidney weight (Fig. S8E), or body weight (Fig. S8F). Histopathological assessment showed that ZZW-115 treatment attenuated glomerulosclerosis and reduced podocyte injury (Fig. 8F), which was further supported by restored expression of podocyte-specific proteins, including nephrin and podocin (Fig. S8G). Moreover, ZZW-115 administration significantly mitigated podocyte loss in DKD mice (Fig. 8G). Ultrastructural examination revealed that DKD mice exhibited podocyte abnormalities, such as binucleation, nuclear degeneration, and foot process effacement, all of which were markedly improved by ZZW-115 treatment (Fig. 8H, I). Immunofluorescence further showed fewer p-H3 -positive podocytes in treated mice (Fig. 8J), indicative of mitigated MC. Consistent with in vitro data, both Western blot (Fig. S8H) and immunofluorescence (Fig. S8I) confirmed that ZZW-115 lowered Cyclin B2 expression, underscoring its ability to suppress aberrant G2/M entry in vivo.

## Discussion

Podocyte injury and death are directly associated with albuminuria and strongly predictive of poor outcomes in DKD [2]. Although podocytes are typically quiescent, pathological stress can induce them to re-enter the cell cycle. However, they lack the capacity to complete mitosis and instead undergo MC, a process that contributes to podocyte depletion rather than regeneration [7, 8]. Therefore, elucidating the mechanisms underlying MC and identifying druggable checkpoints represent urgent clinical priorities.

To investigate the pathogenesis of DKD and identify potential therapeutic targets, we performed single-cell RNA sequencing on renal tissues from 22-week-old db/m and db/db mice (*n* = 4 per group). Our analysis revealed that NUPR1 is predominantly and specifically expressed in podocytes and is significantly upregulated under DKD conditions.

As a transcriptional regulator, NUPR1 has been implicated in various biological processes across multiple diseases [21]. In this study, we confirmed its podocyte-specific expression and demonstrated its significant upregulation in DKD. Importantly, NUPR1 protein levels were positively correlated with urinary protein excretion, suggesting its clinical relevance in DKD progression. These findings enhance our understanding of NUPR1’s cell-specific distribution and functional role in podocyte injury. Previous studies have reported NUPR1 expression in mesangial and renal tubular epithelial cells [16–18], however, under diabetic conditions, we did not detect expression of this gene in these cell types. The discrepancy between our findings and earlier reports may be due to disease-induced alterations in expression patterns, as well as a lack of in vivo validation in previous studies.

To elucidate the mechanism by which NUPR1 regulated podocyte injury, we performed transcriptome sequencing. Our data revealed that NUPR1 was enriched in pathways related to the cell cycle and mitosis HG treatment markedly increased the number of binucleated podocytes with abnormal mitotic spindles, indicating entry into MC, a process exacerbated by NUPR1 overexpression. Consistently, DKD mice exhibited podocyte binucleation, nuclear degeneration, and detachment, all of which were more severe in NUPR1-overexpressing mice. Collectively, these data establish that NUPR1 promotes MC in podocytes.

Previous studies have suggested that restricting podocytes to the G1 phase of the cell cycle may partially compensate for podocyte loss through hypertrophy [8]. However, progression into the G2/M phase often results in the formation of aneuploid podocytes, which are more susceptible to injury and exhibit a greater tendency toward detachment and apoptosis [9, 22]. We found that HG stimulation promoted podocyte entry into the G2/M phase. Notably, NUPR1 overexpression not only facilitated aberrant cell cycle progression but also increased apoptosis and exacerbated MC. Conversely, NUPR1 knockdown suppressed the G2/M transition and protected podocytes from MC.

Mechanistically, NUPR1 functions as a transcriptional activator of Cyclin B2, a key regulator of the G2/M transition and spindle assembly [23]. Elevated Cyclin B2 levels can induce spindle defects and chromosomal missegregation, leading to aneuploidy and multinucleation [23]. We demonstrated that NUPR1 directly binds to the Cyclin B2 promoter and activates its transcription, likely via interaction with BRD2. Knockdown of either NUPR1 or BRD2 significantly reduced Cyclin B2 transcriptional activity. Importantly, silencing Cyclin B2 alone was sufficient to abolish NUPR1-induced MC, indicating that NUPR1 primarily exerts its deleterious effects through Cyclin B2 upregulation.

From a therapeutic perspective, the small-molecule inhibitor ZZW-115 disrupts the NUPR1-BRD2 interaction by inhibiting NUPR1 nuclear translocation, thereby downregulating Cyclin B2 expression. This alleviates cell cycle dysregulation, prevents MC, and reduces podocyte apoptosis and loss. In vivo, ZZW-115 demonstrated dose-dependent safety and significant efficacy in attenuating proteinuria in DKD mice, without affecting blood glucose, kidney weight, or body weight. These findings indicate that ZZW-115 exerts its therapeutic effects through direct podocyte protection, rather than systemic metabolic modulation. Notably, ZZW-115 suppressed podocyte MC and reduced podocyte loss, leading to alleviation of DKD progression. Collectively, these results highlight the strong translational potential of ZZW-115 as a targeted therapy for DKD.

This study has several limitations. First, while we identified a novel role for NUPR1 in podocyte MC, the upstream molecular cues driving its upregulation under DKD conditions remain unclear. Although this study primarily focuses on the downstream effects of NUPR1, the upstream mechanisms underlying its transcriptional upregulation in diabetic kidney disease (DKD) remain to be fully elucidated. The promoter region of NUPR1 contains multiple potential transcription factor binding sites, including those for p53 and NF-Κb [24]. Under the pathological conditions of DKD, hyperglycemia, hemodynamic abnormalities, and oxidative stress can activate these transcription factors, which may synergistically promote the transcriptional upregulation of NUPR1. Furthermore, epigenetic regulatory mechanisms, such as aberrant histone acetylation modifications observed in DKD, may also play a crucial role in this process [25–28]. Systematic elucidation of the molecular basis for NUPR1 upregulation will contribute to a comprehensive understanding of its initiating and amplifying effects in DKD-associated podocyte injury. Subsequent studies can screen the upstream regulatory factors of NUPR1 through transcriptome/protein interaction assays. Second, the absence of podocyte-specific genetic models limits our ability to exclude compensatory effects from other renal cell types. Nevertheless, our data show that NUPR1 is selectively enriched in podocytes, and the podocyte-preferential action of ZZW-115 supports its therapeutic specificity. Finally, while the NUPR1/BRD2/ Cyclin B2 axis is a key pathway, additional regulators of MC may exist. Future studies should systematically map the broader NUPR1-dependent transcriptional network to fully elucidate its mechanistic impact.

In summary, this study demonstrates that NUPR1 promotes aberrant cell cycle progression, MC, and renal injury in DKD, primarily through the NUPR1/BRD2/Cyclin B2 signaling axis. Pharmacological inhibition of NUPR1 via ZZW-115 offers a promising therapeutic strategy for DKD treatment.

## Materials and methods

Detailed descriptions of the materials and methods are provided in the Supplementary Materials.

### Human renal tissue specimens

After written informed consent was obtained from all participants, renal biopsy samples were collected from 40 patients diagnosed with DKD. Normal renal tissues were obtained from 10 patients who underwent nephrectomy for renal cell carcinoma and served as controls. The clinical characteristics of all 50 participants are summarized in the Supplementary Materials. This study was conducted in accordance with the Declaration of Helsinki and approved by the Research Ethics Committee of Guangdong Provincial People’s Hospital (Approval No: KY2024-1074-02).

### Animal experiments

Six-week-old male C57BL/6 mice were purchased from Guangdong Medical Laboratory Animal Center. All animal experiments were performed in accordance with the Guide for the Care and Use of Laboratory Animals and were approved by the Research Ethics Committee of Guangdong Provincial People’s Hospital (Approval No: KY2024-1074-02). All experiments were conducted with 6 mice per group.

DKD mice were established as previously described [29] by combining streptozotocin (STZ; Sigma, S0130), unilateral nephrectomy (UNX), and a high-fat diet (HFD; 60% kcal from fat; Research Diets, D12492). The mice were euthanized at 24 weeks of age for tissue collection and subsequent analyses.

For hydrodynamic gene delivery [30], mice were intravenously injected with an adeno-associated viral vector carrying NUPR1 (pHBAAV-NUPR1) or a control vector (pHBAAV-null) at 12 and 18 weeks of age, respectively.

For pharmacological inhibition, DKD mice received intraperitoneal injections of the NUPR1 inhibitor 5 or 10 mg/kg ZZW-115 (Selleck Chemicals, E1037) [19, 20] or an equivalent volume of dimethyl sulfoxide (DMSO; vehicle control) every other day for 8 weeks, starting at 17 weeks of age.

### Statistical analysis

The data are expressed as the means ± standard errors of the means (SEMs). All the statistical analyses were conducted via GraphPad Prism software (version 8.0; USA). Detailed descriptions of the statistical methods are provided in the Supplementary Material.

## Supplementary information

Supplementary Materials

Supplementary Tables

Uncropped WB images

## Data Availability

The datasets generated and analyzed during the current study are available from the corresponding author upon reasonable request.
