# Supplementary material for: NUPR1 inhibition prevents mitotic catastrophe: a novel therapeutic strategy for podocyte injury in diabetic kidney disease
Source: Cell Death Dis. 2026 Aug 12;17(1):950. doi: 10.1038/s41419-026-09152-8 (PMC13745598; doi:10.1038/s41419-026-09152-8)

Figure 2A

β-tubulin

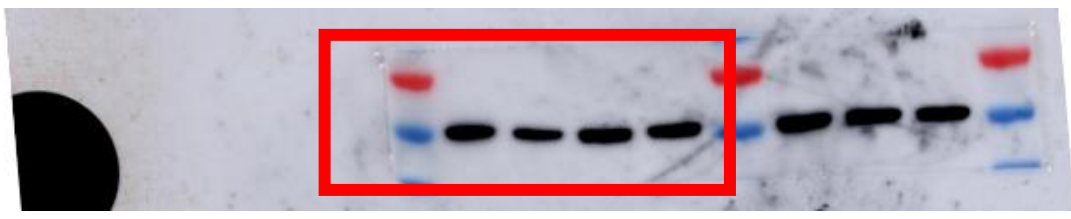

NUPR1

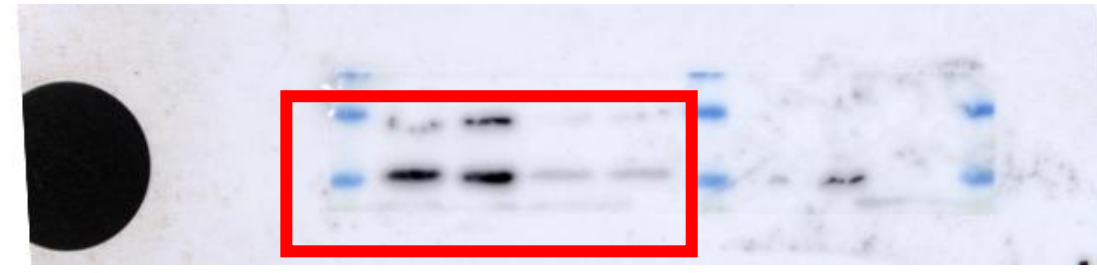

Figure 2B

GAPDH

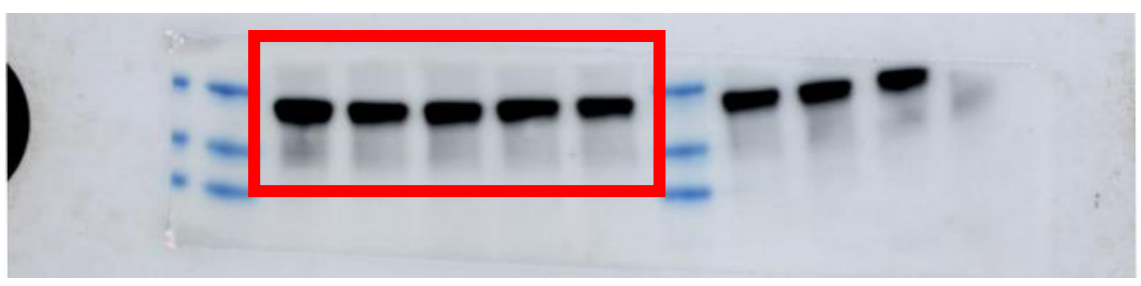

NUPR1

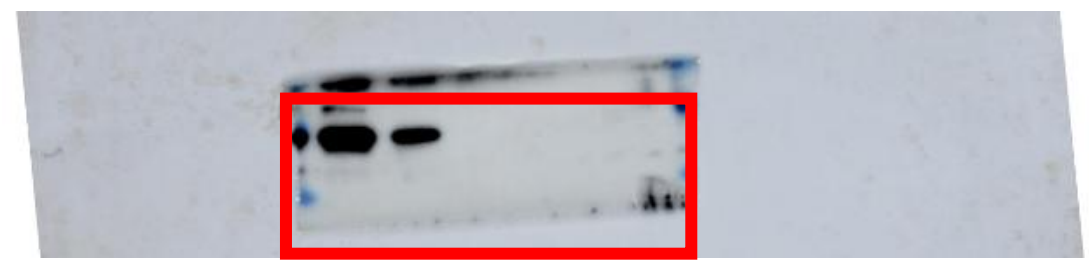

Figure 2C

GAPDH

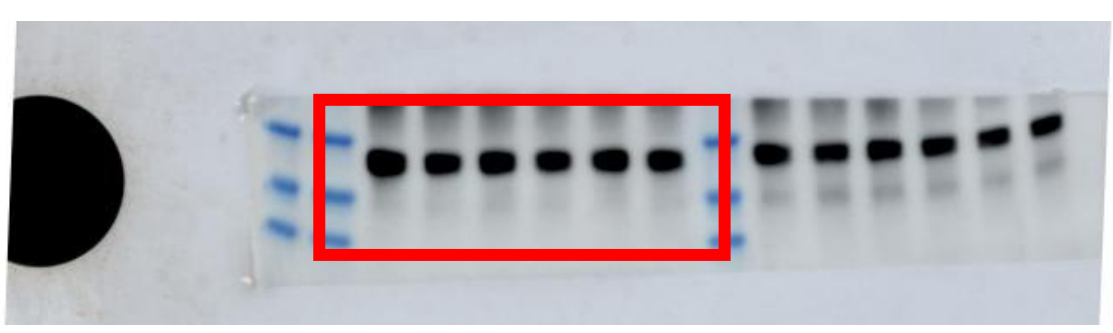

Nephrin

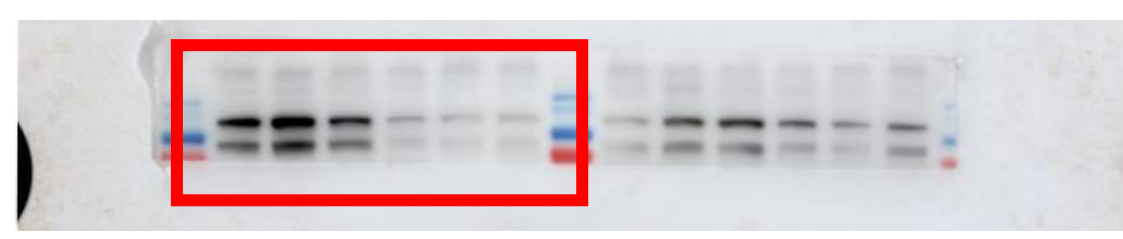

NUPR1

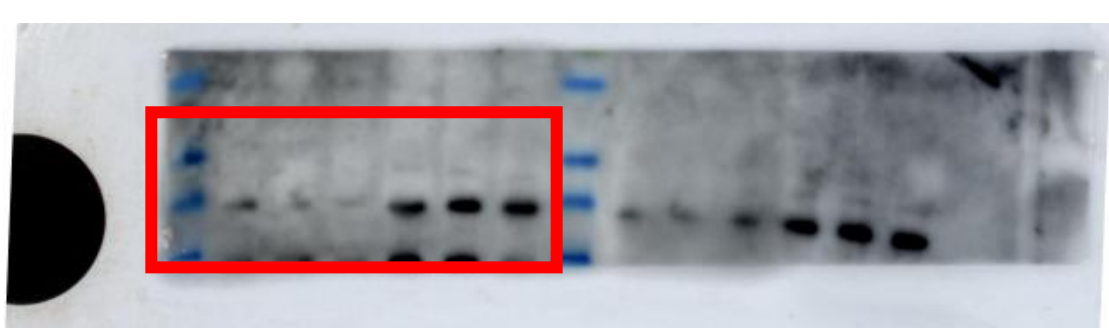

Figure 2E

GAPDH

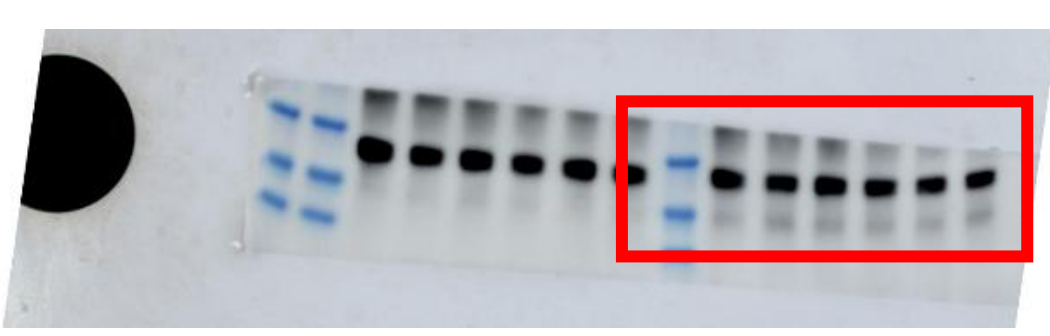

Nephrin

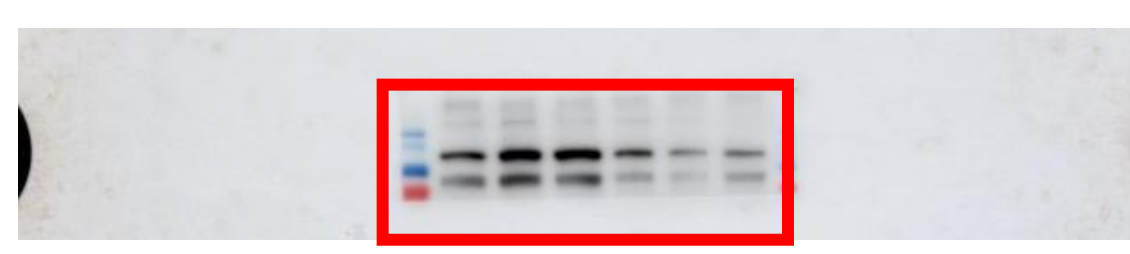

NUPR1

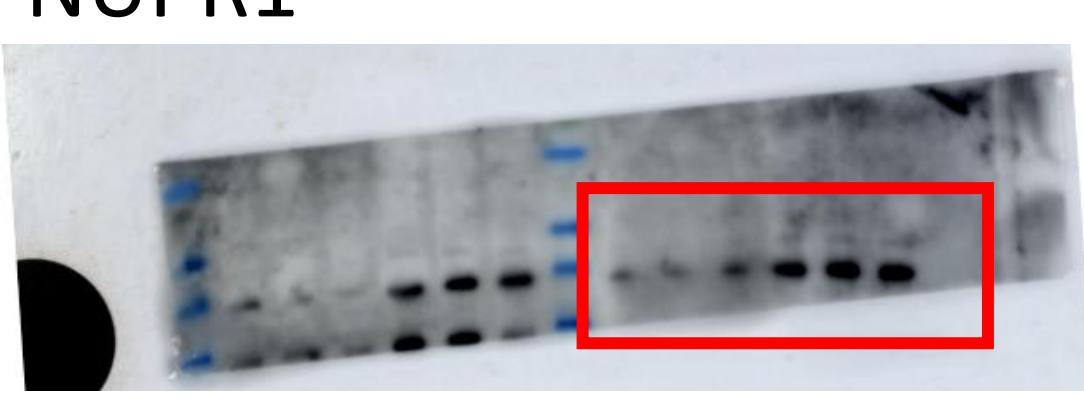

Figure 2G

GAPDH

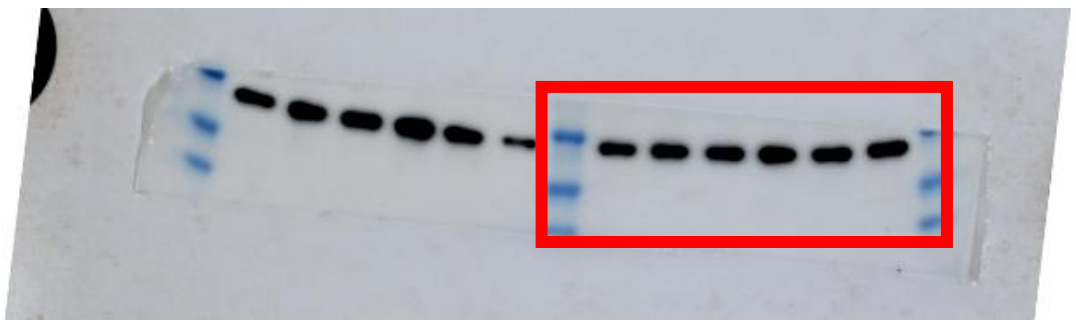

NUPR1

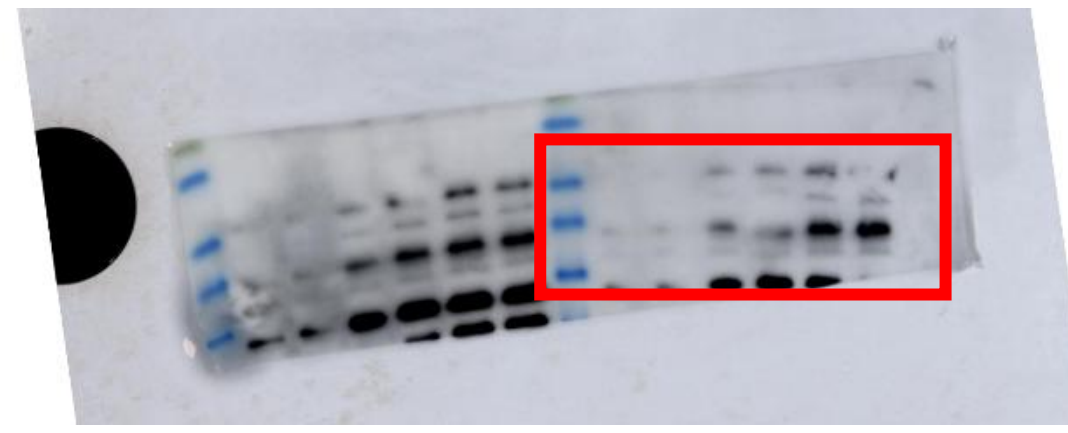

Podocin

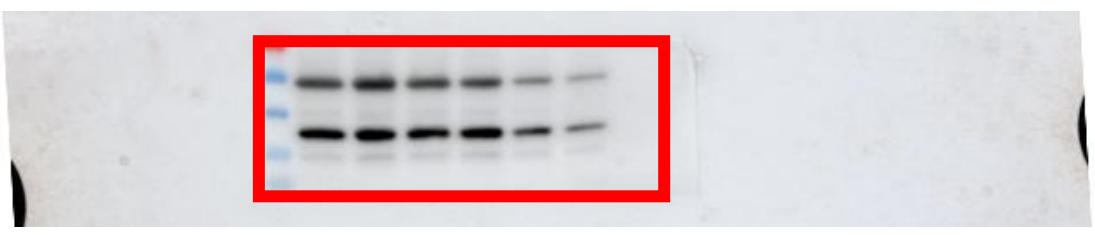

WT1

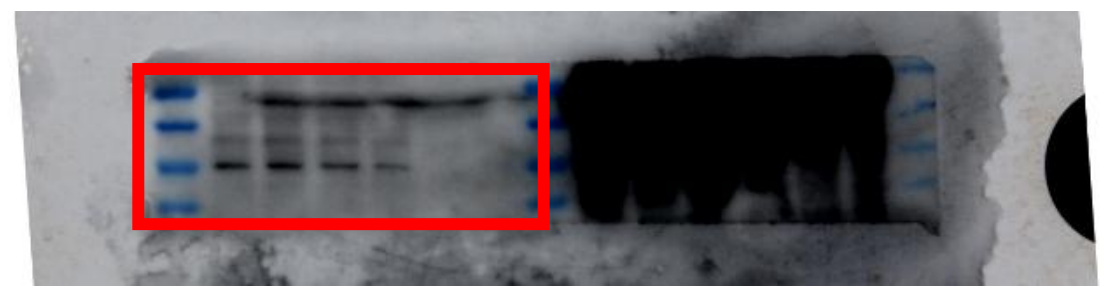

Figure 2H

GAPDH

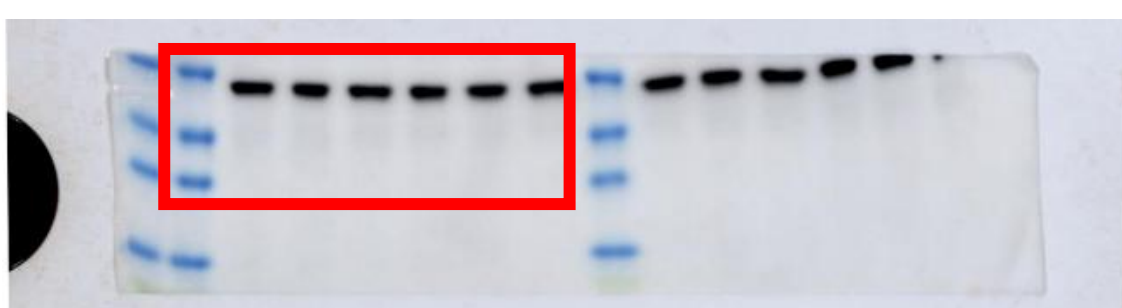

Nephrin

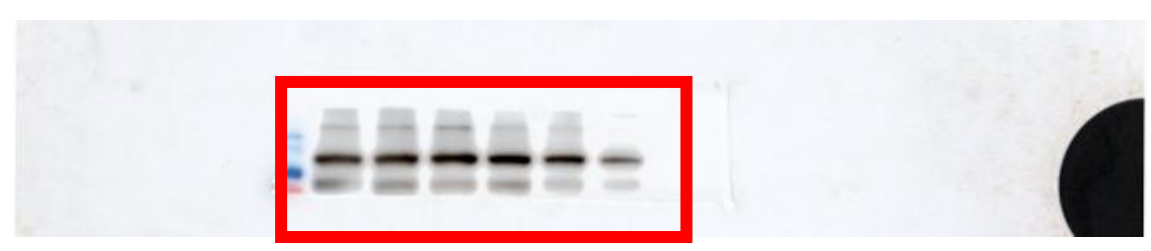

NUPR1

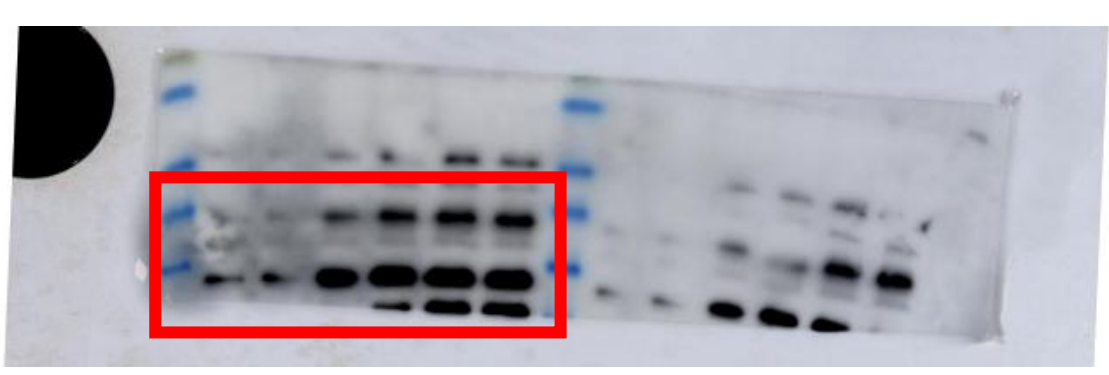

Podocin

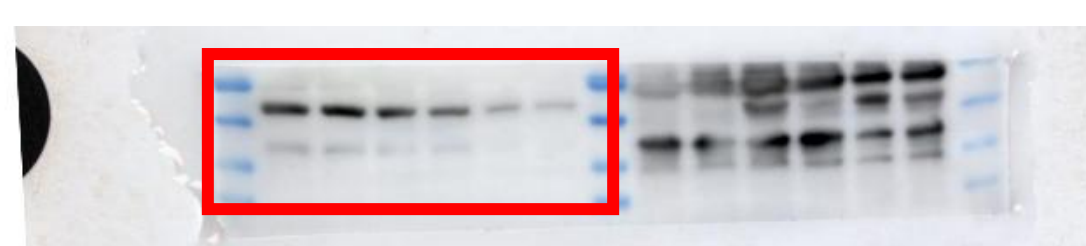

Figure 3D

Nephrin

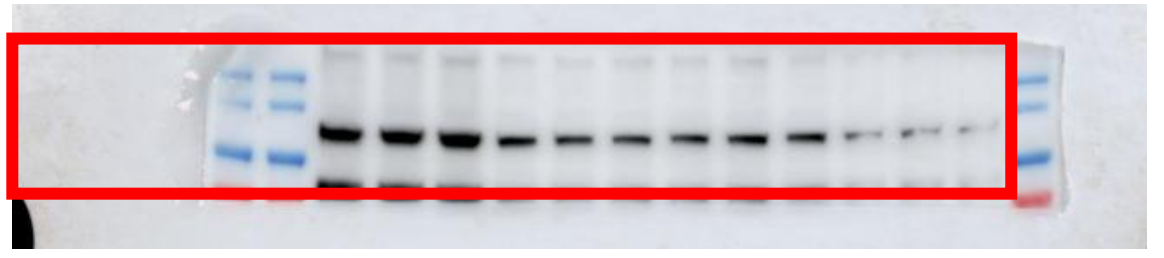

Podocin

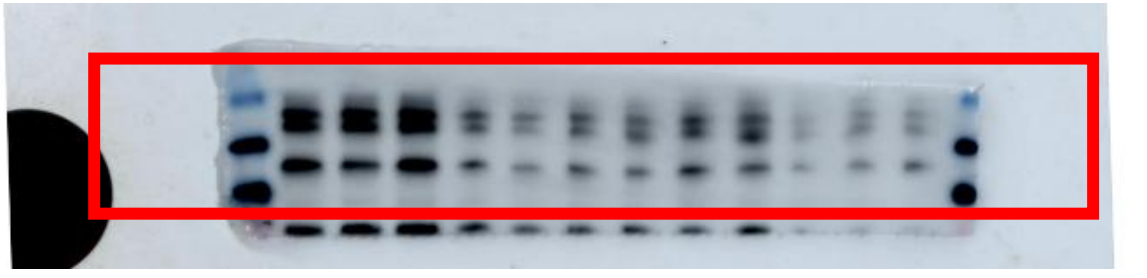

NUPR1

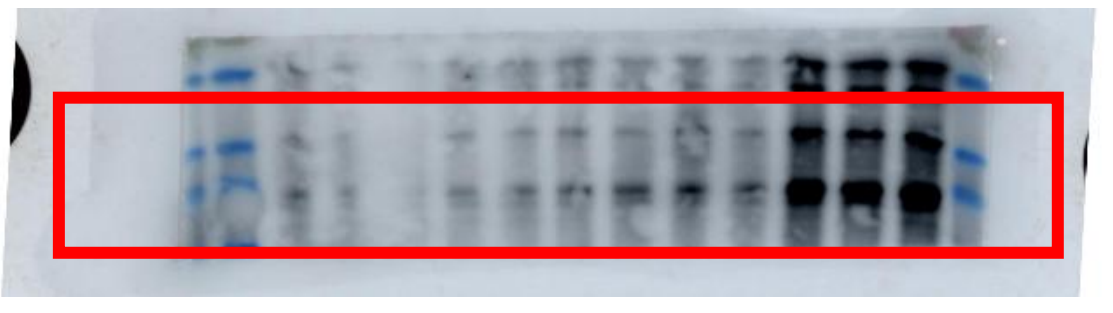

GAPDH

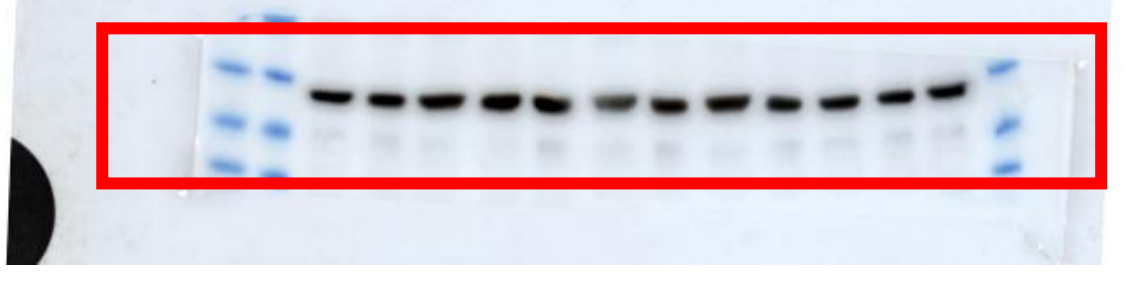

Figure S2B

Synaptopodin

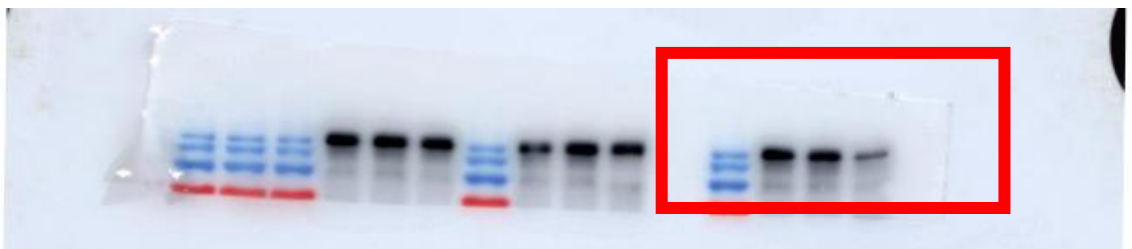

Podocin

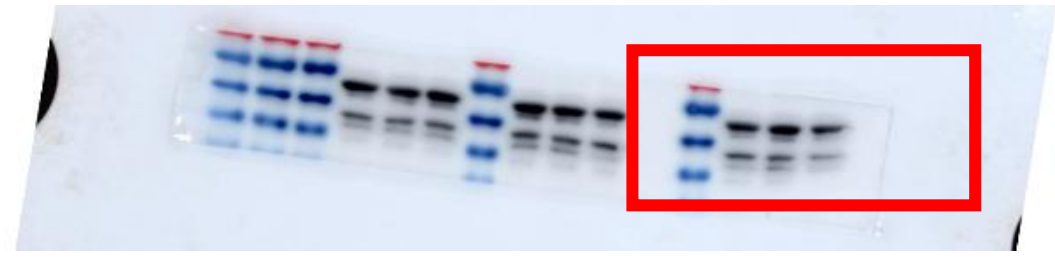

NUPR1

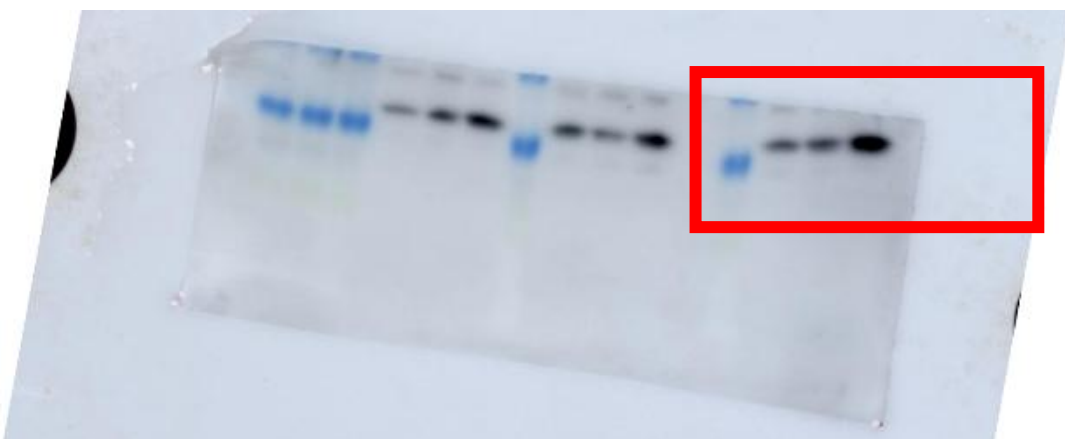

GAPDH

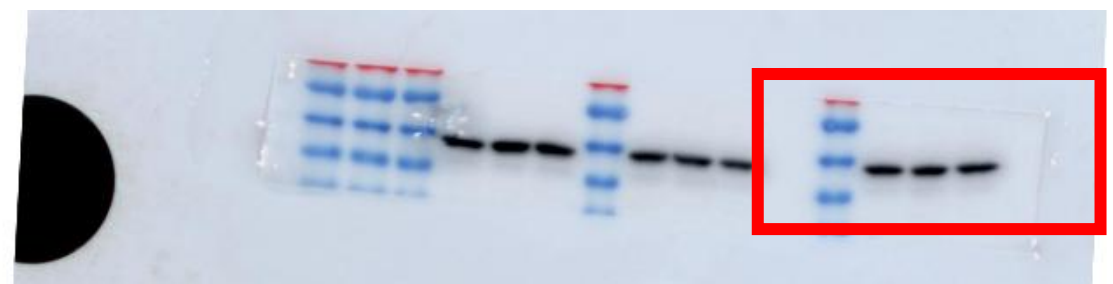

Figure S2D

GAPDH

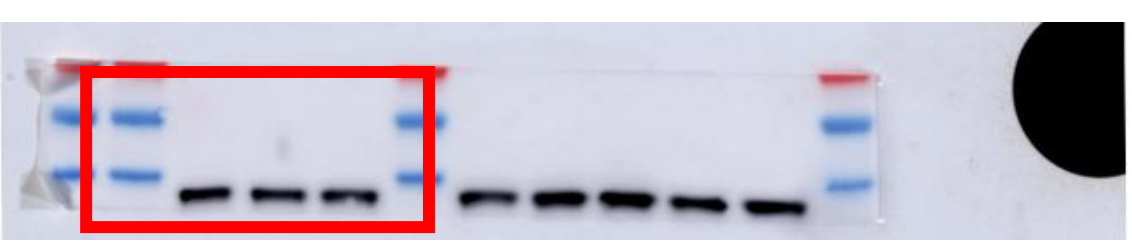

NUPR1

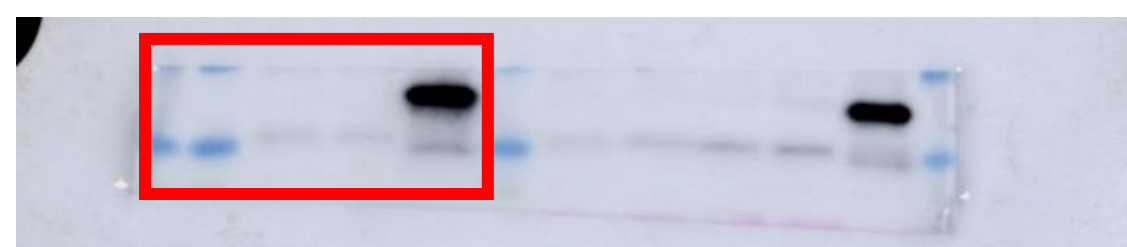

Figure S2F

GAPDH

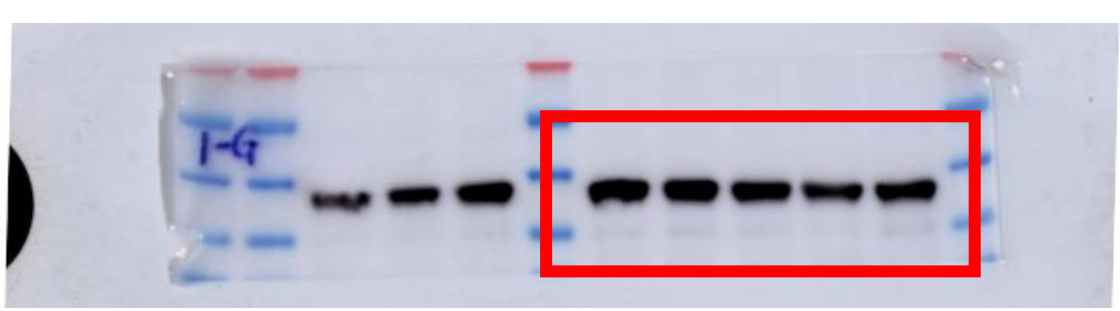

NUPR1

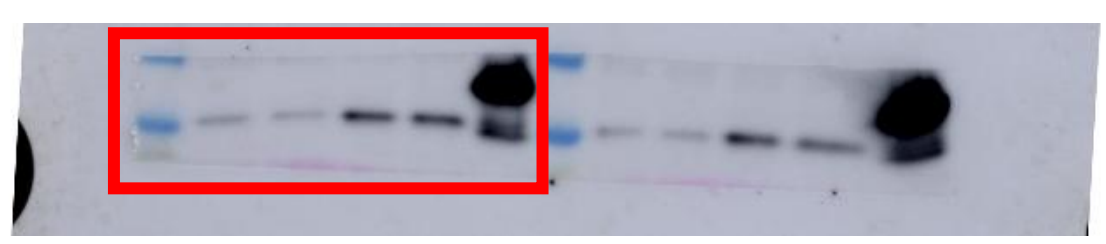

Podocin

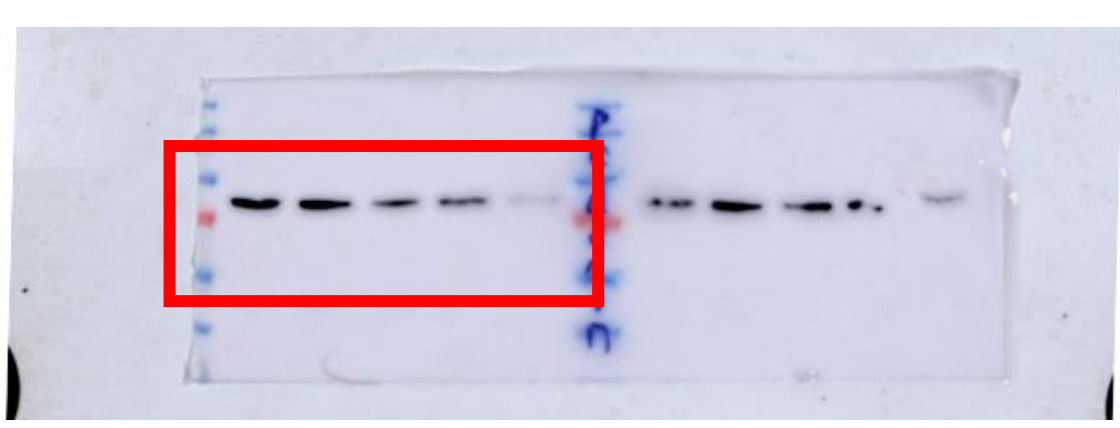

Synaptopodin

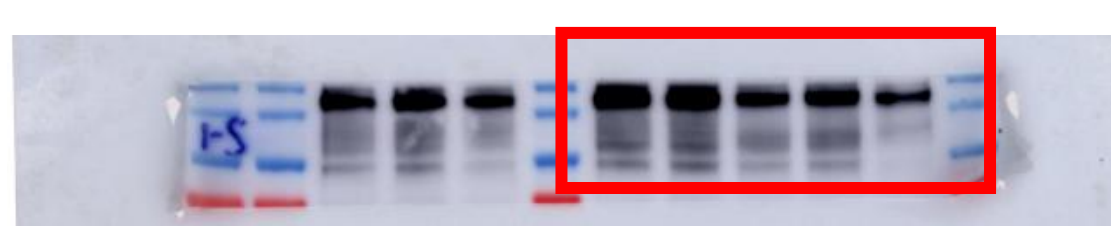

Figure S2H

GAPDH

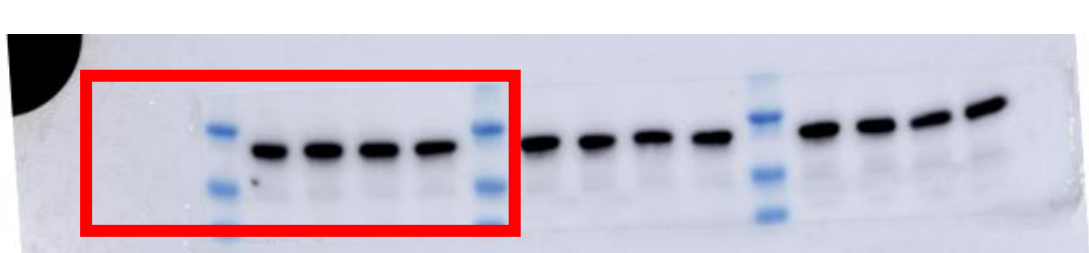

NUPR1

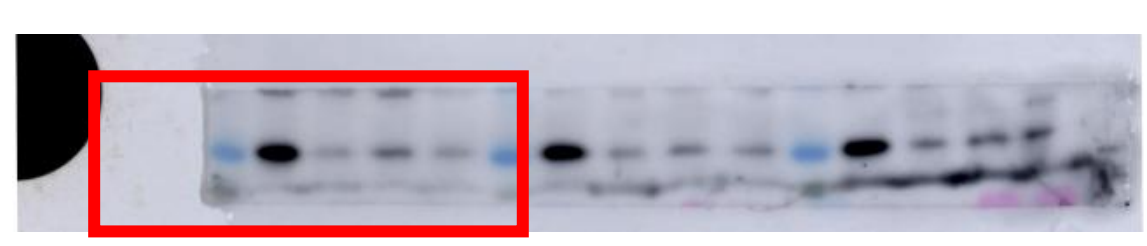

Figure S2I

GAPDH

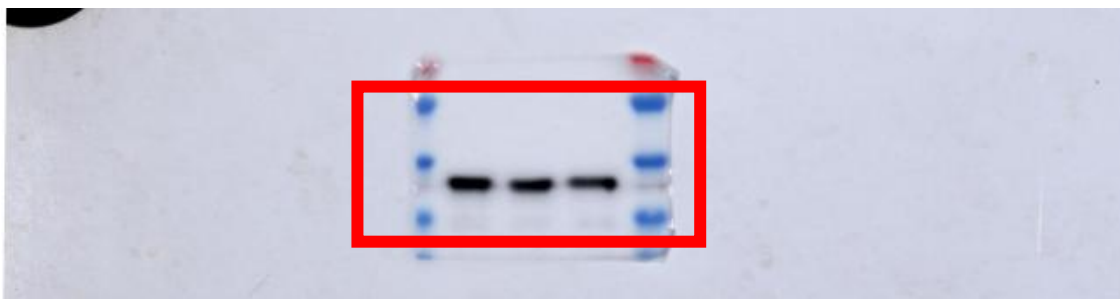

NUPR1

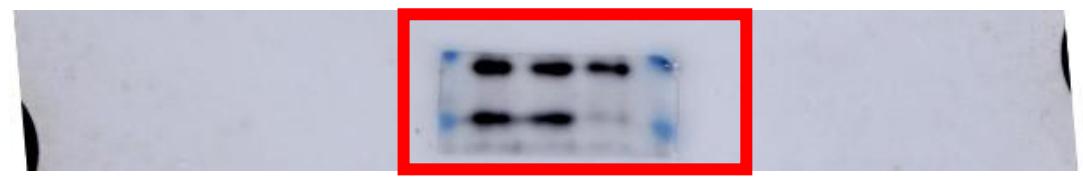

Figure S2K

GAPDH

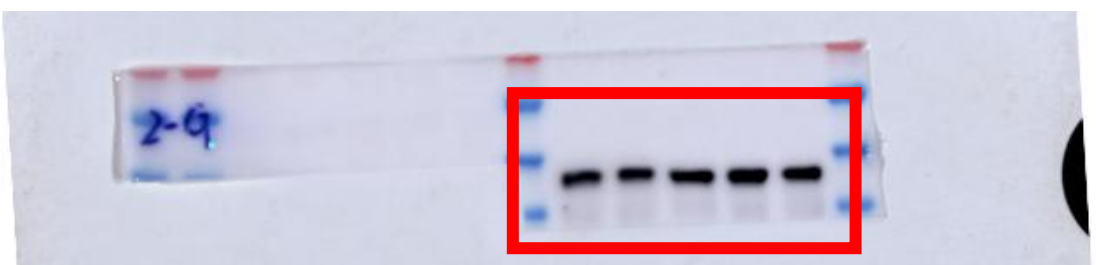

NUPR1

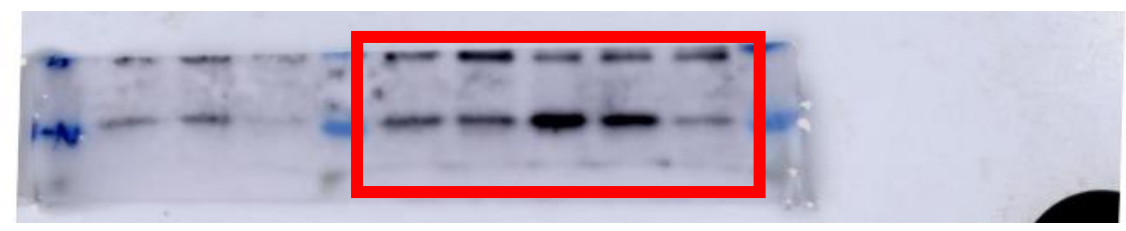

Podocin

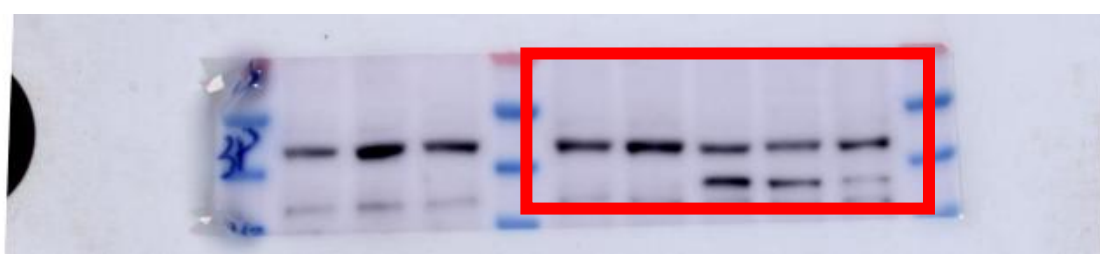

Synaptopodin

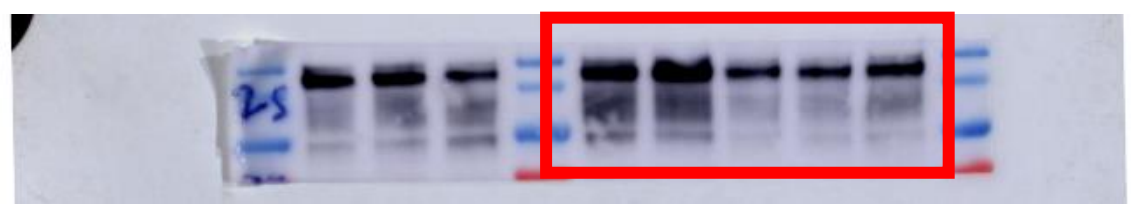

Figure 5C

GAPDH

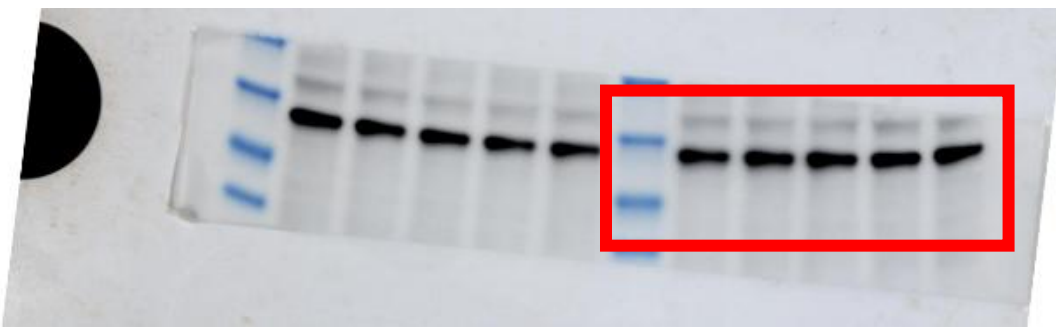

NUPR1

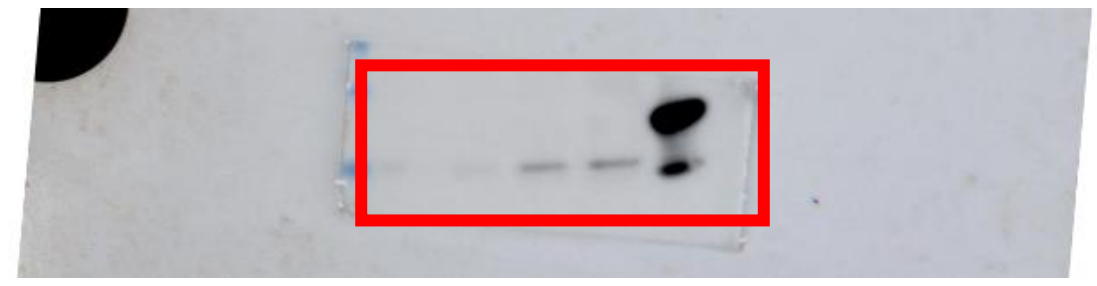

Cleaved-Caspase3

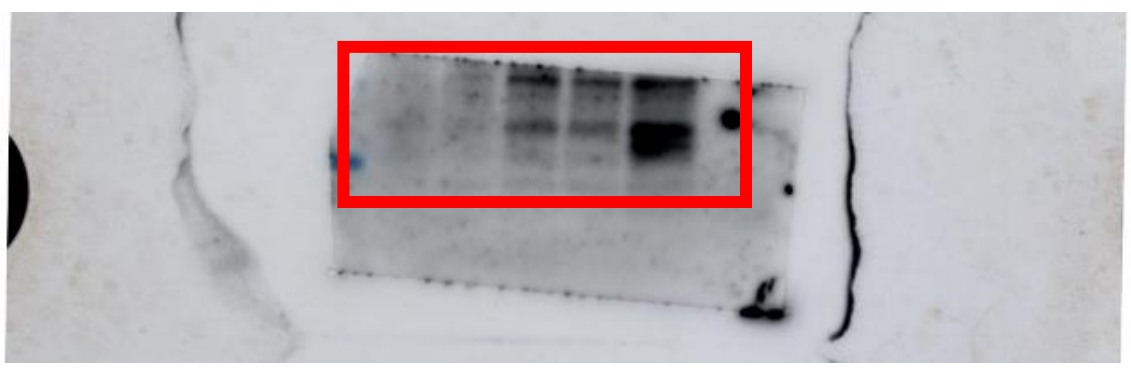

Caspase3

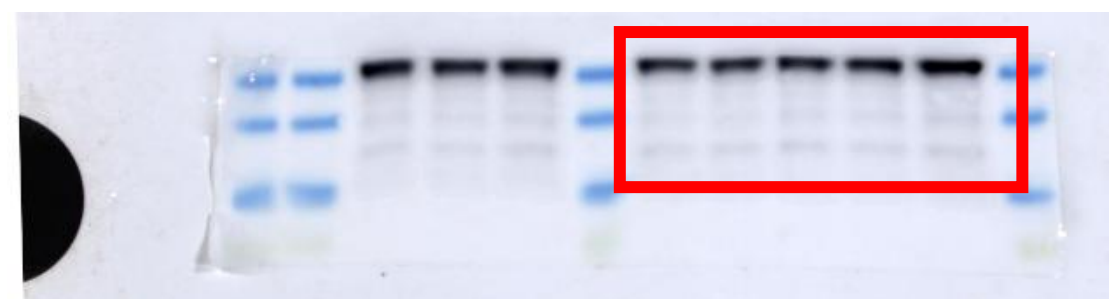

Figure 5D

GAPDH

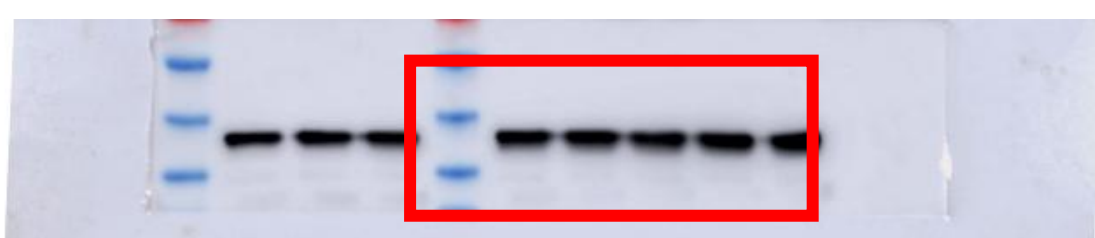

NUPR1

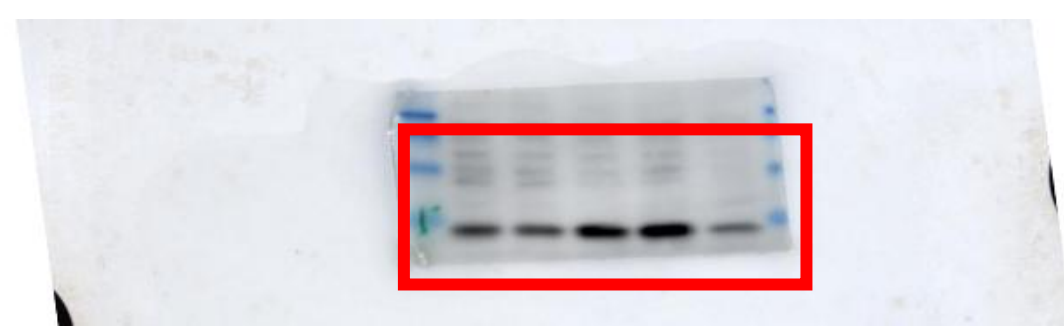

Cleaved-Caspase3

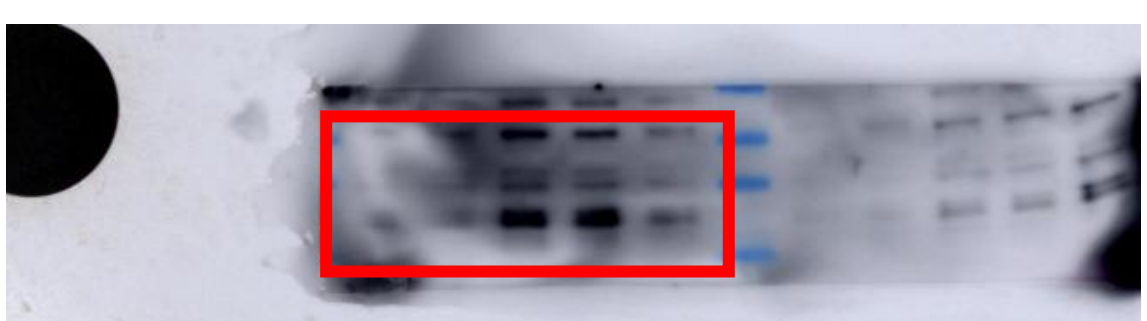

Caspase3

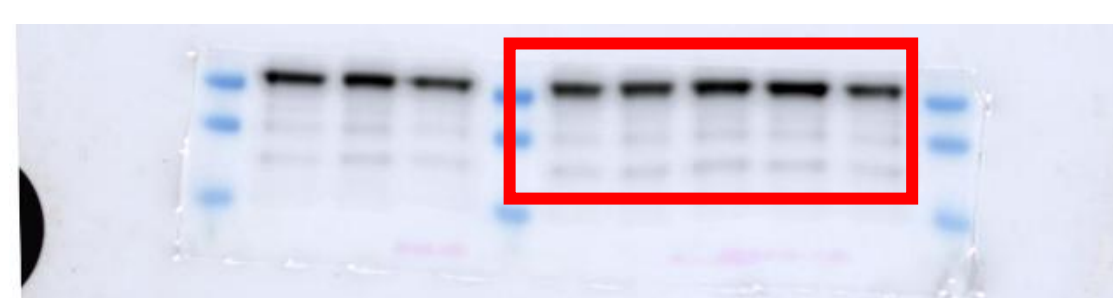

Figure 6B

CyclinB2

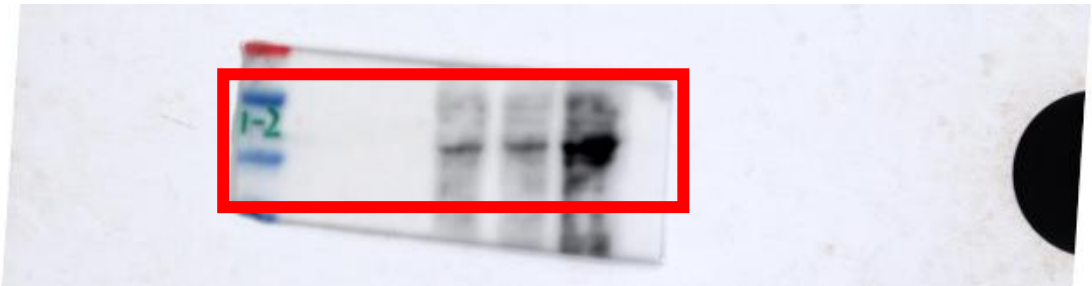

NUPR1

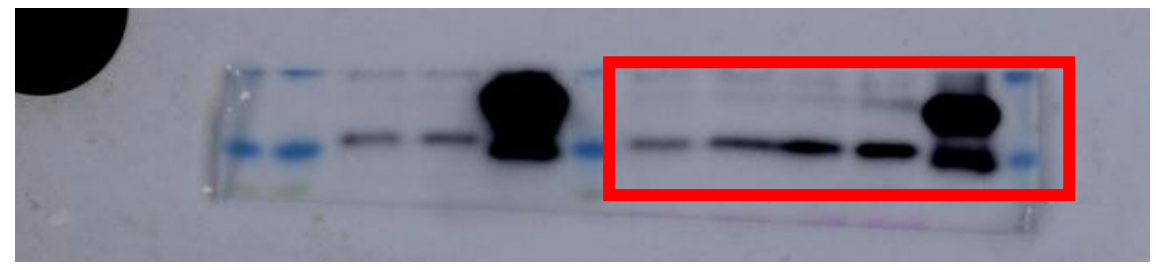

GAPDH

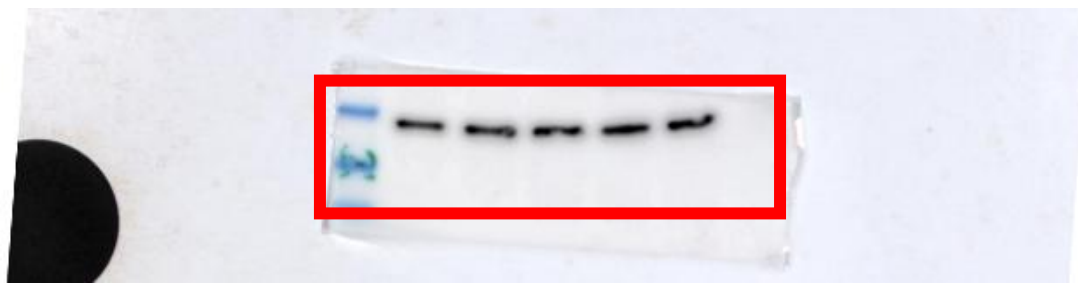

Figure 6C

NUPR1

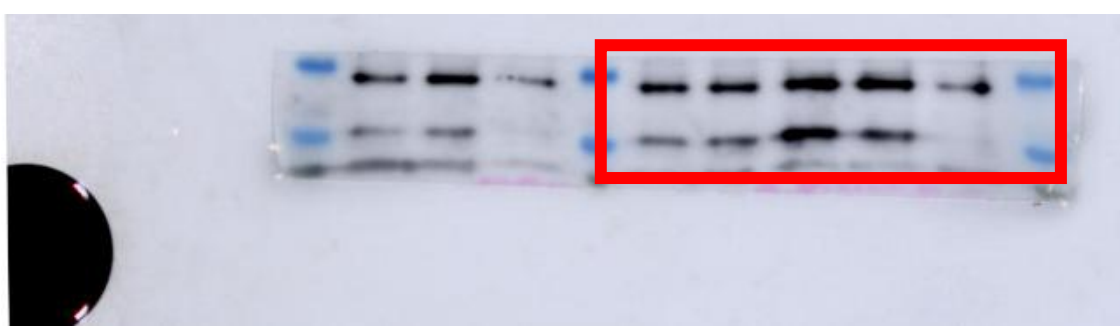

GAPDH

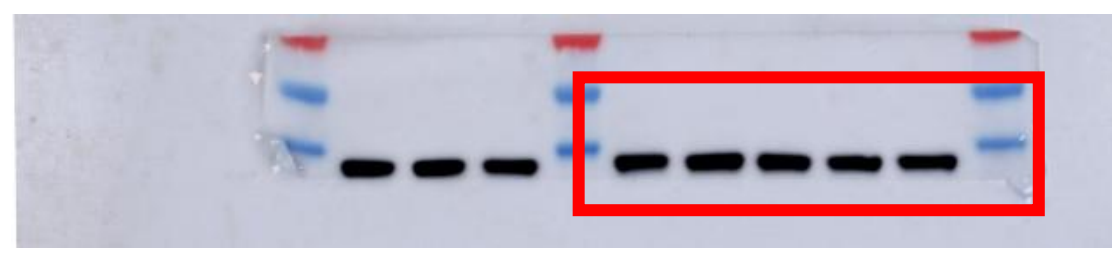

CyclinB2

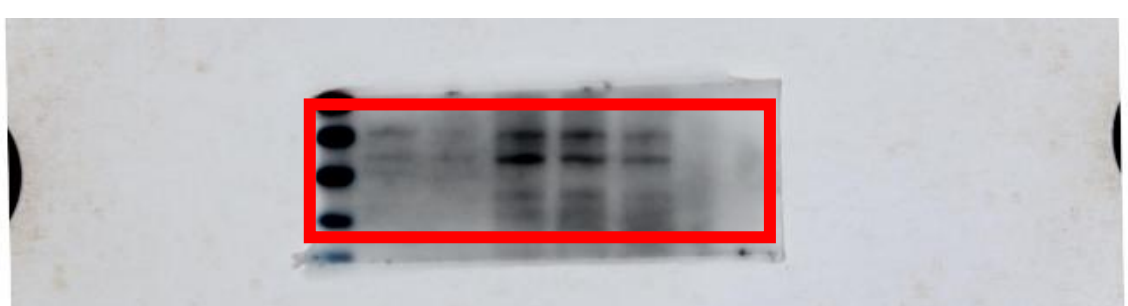

Figure 6L

CyclinB2

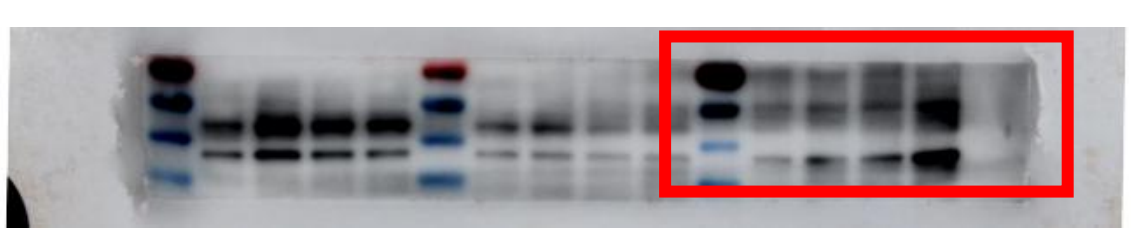

GAPDH

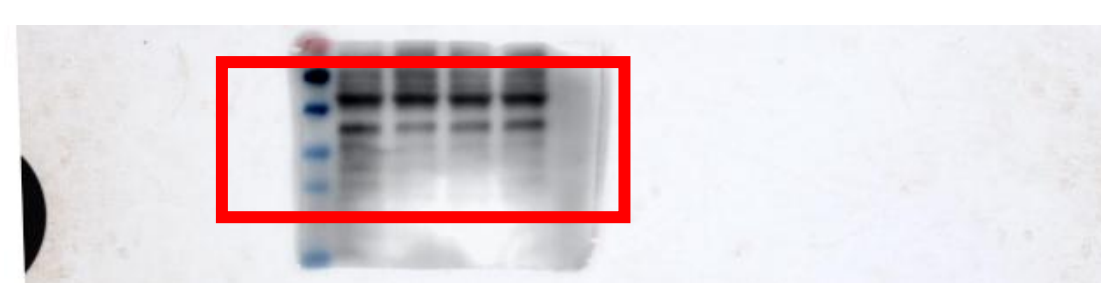

Figure S4B

CyclinB2

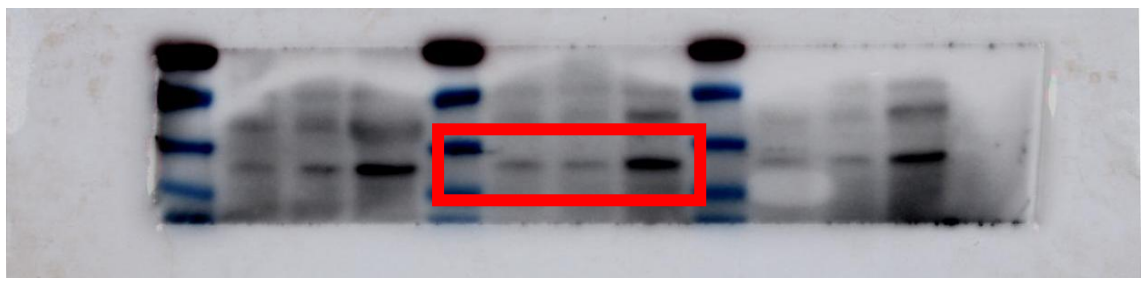

GAPDH

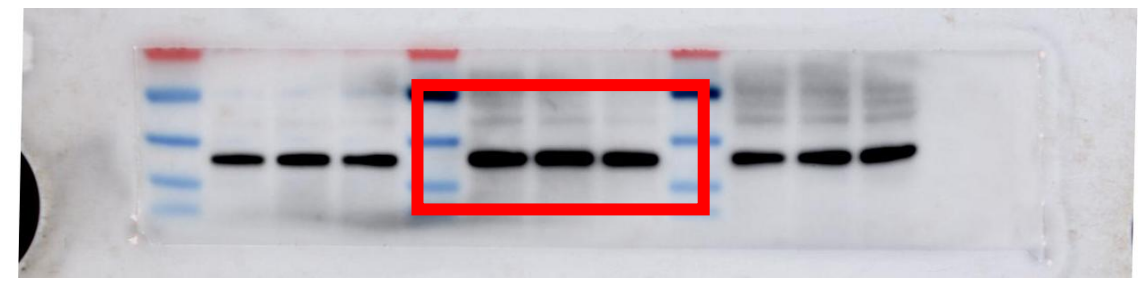

Figure S4D

CyclinB2

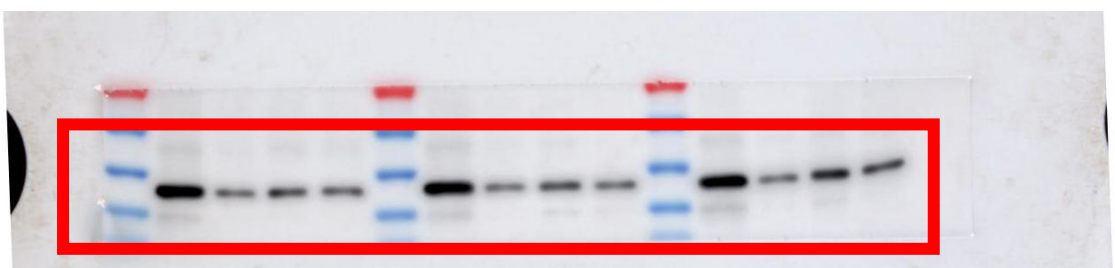

GAPDH

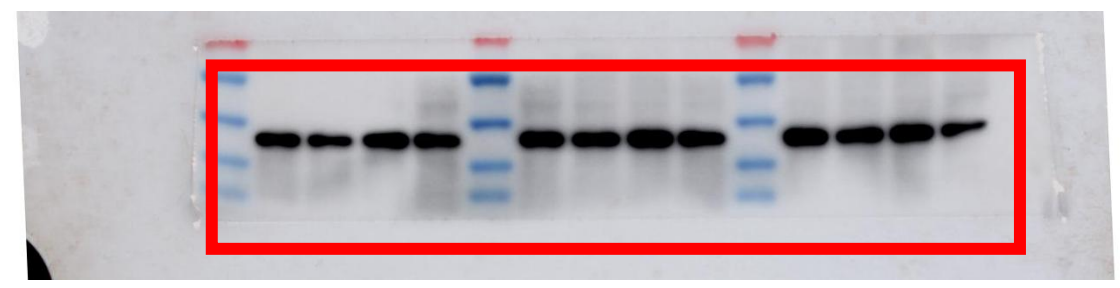

Figure S4F

CyclinB2

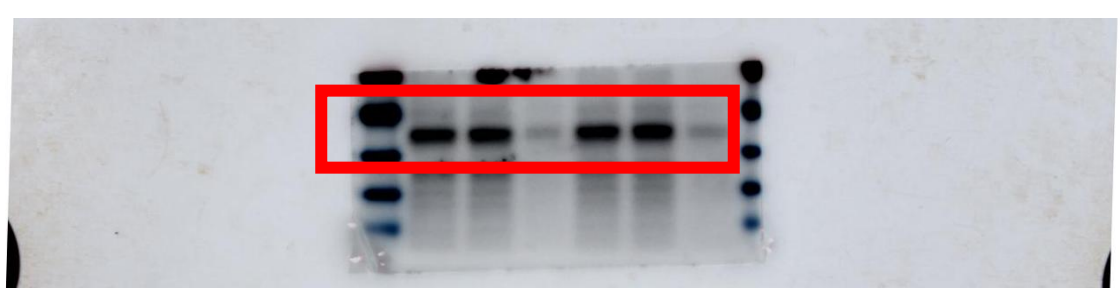

GAPDH

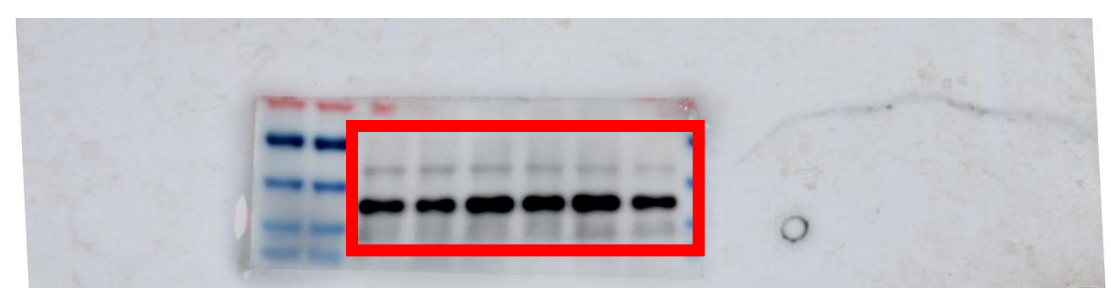

Figure S4H

CyclinB2

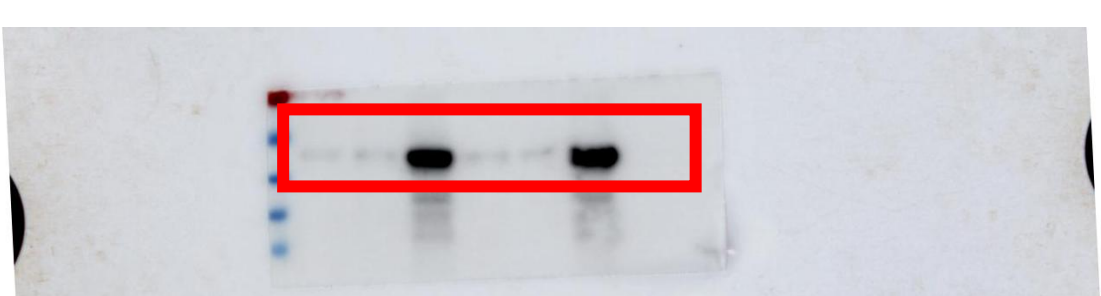

GAPDH

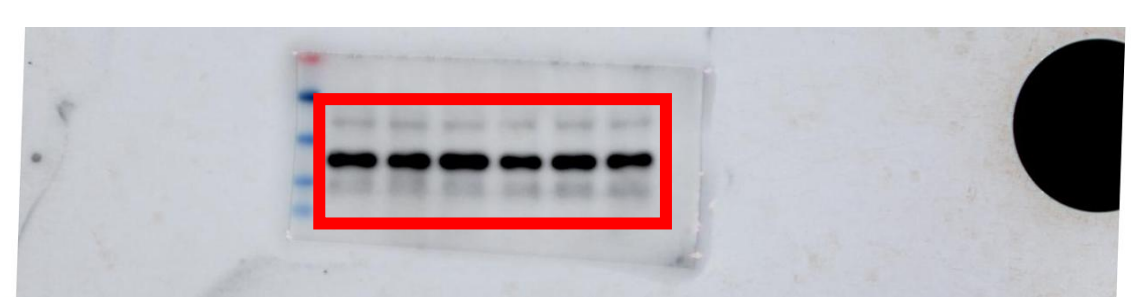

Figure 7B

BRD2

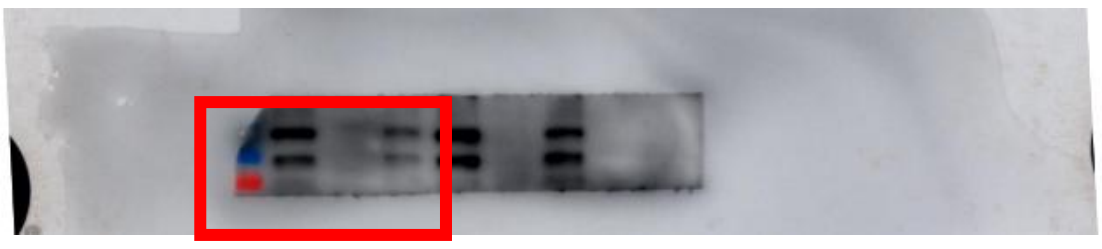

BRD2

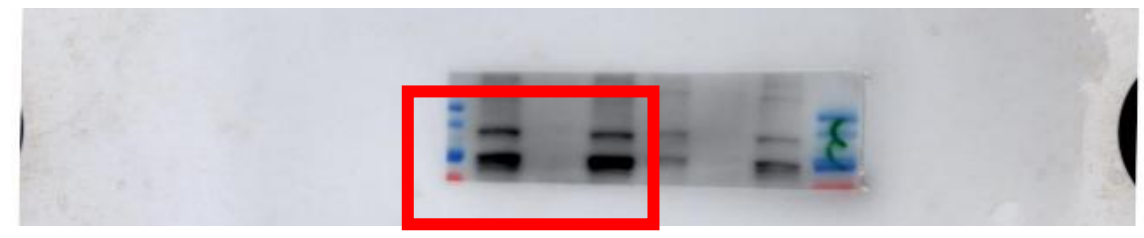

Figure 7C

NUPR1

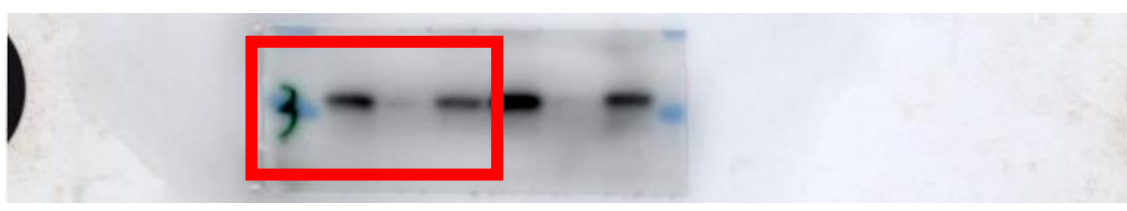

NUPR1

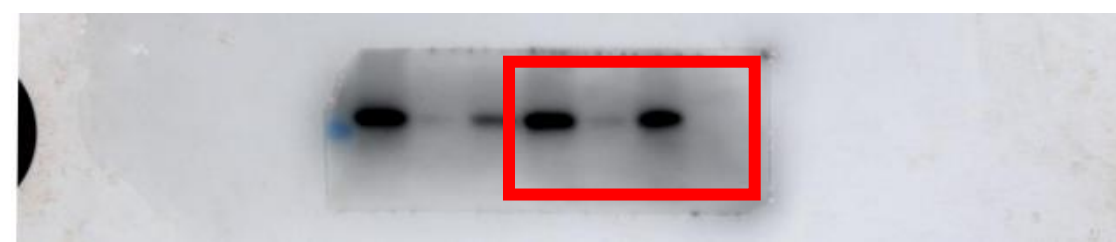

Figure 7L

BRD2

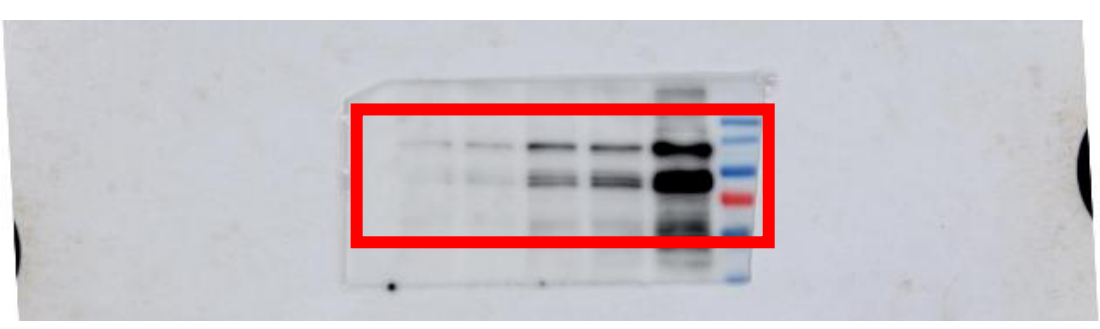

Cyclin B2

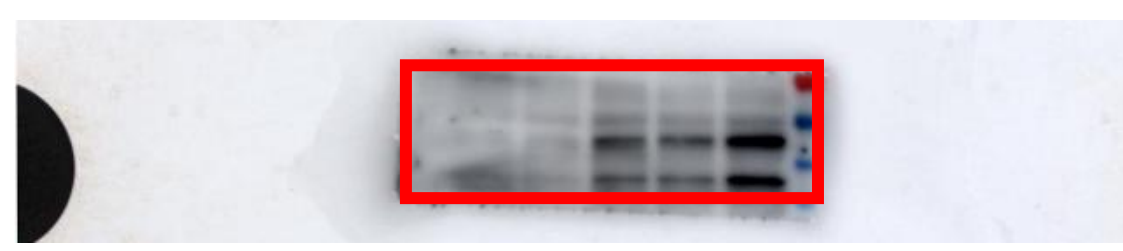

GAPDH

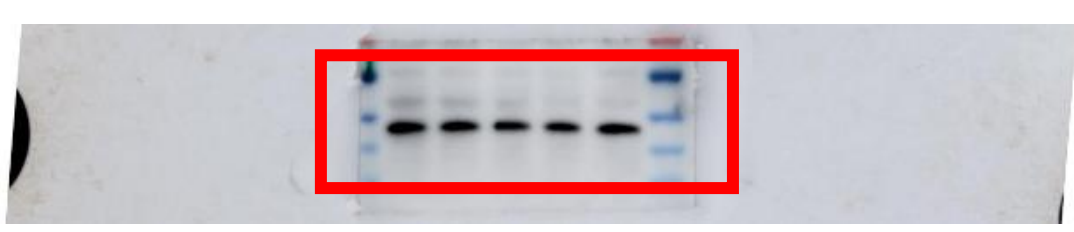

Figure 7M

BRD2

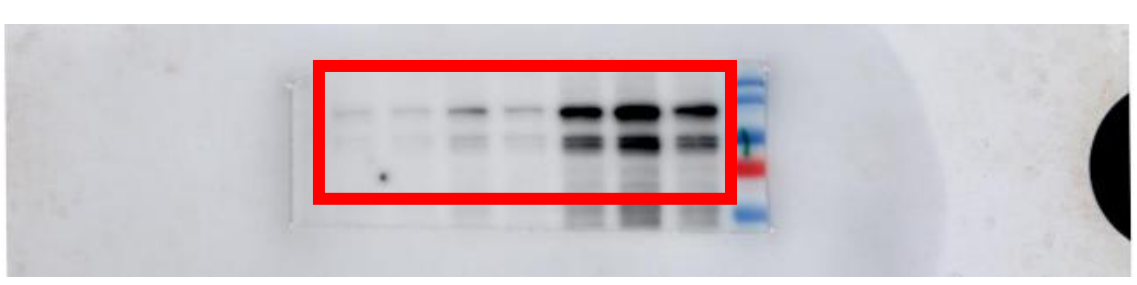

Cyclin B2

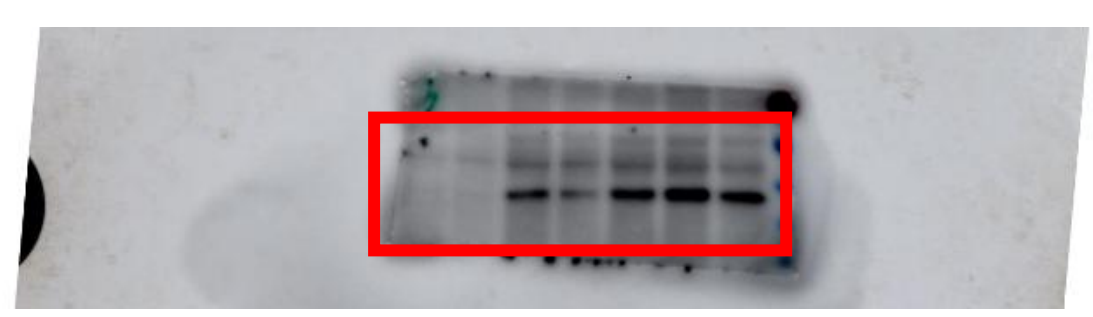

GAPDH

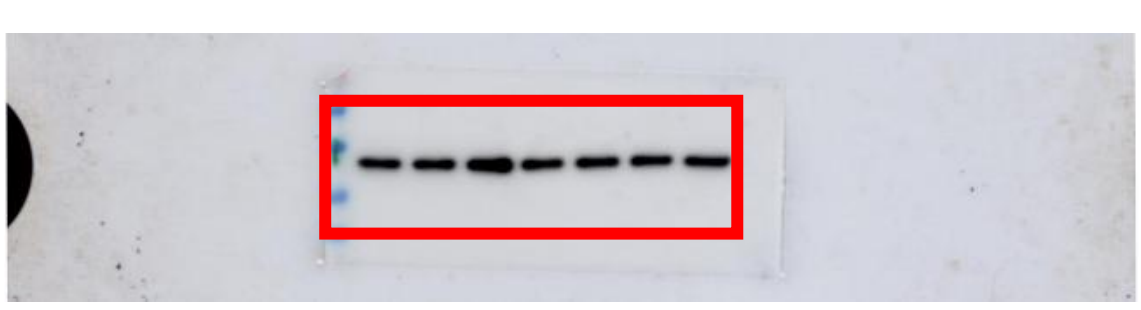

NUPR1

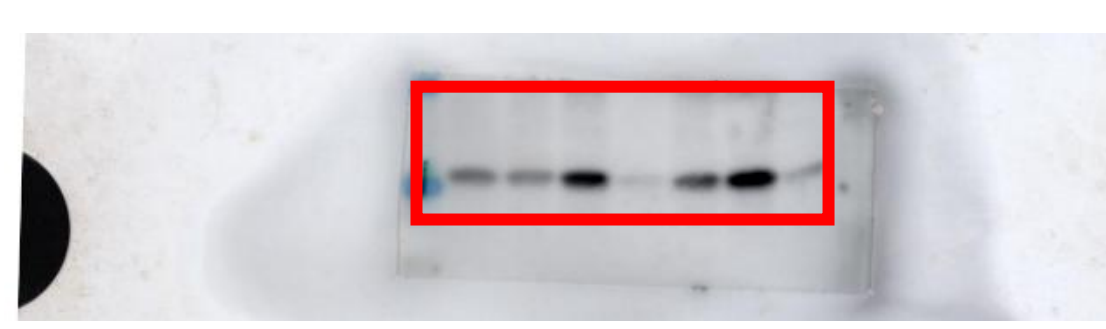

Figure 7N

BRD2

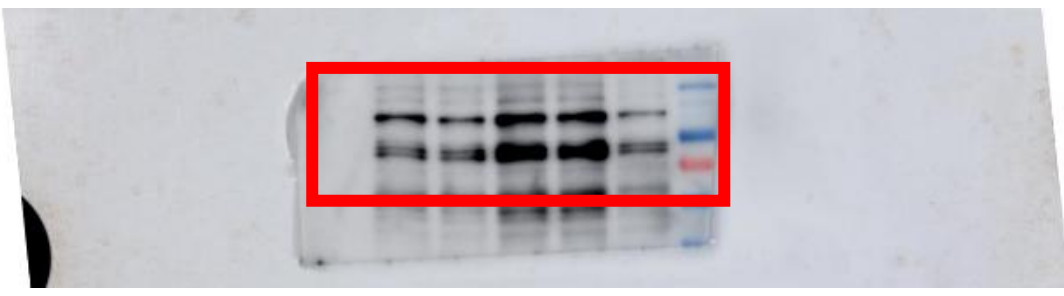

Cyclin B2

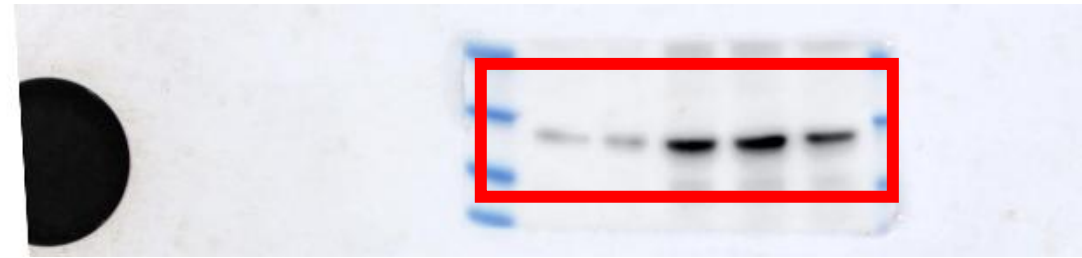

GAPDH

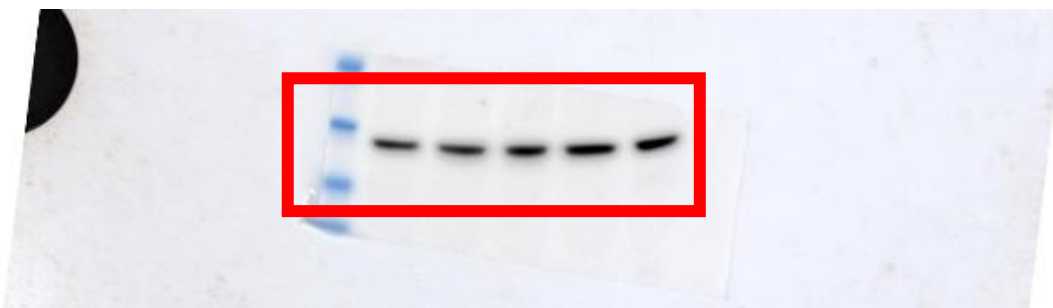

Figure 7O

BRD2

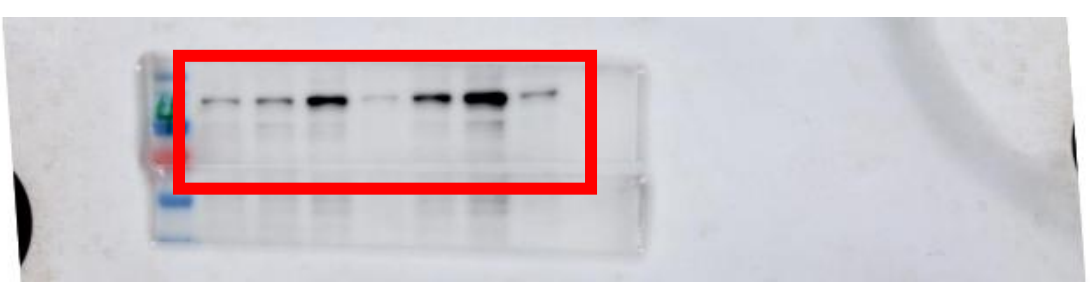

Cyclin B2

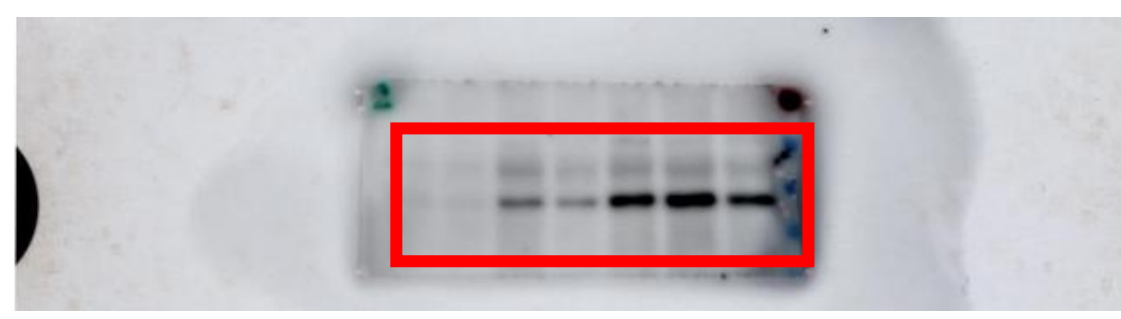

GAPDH

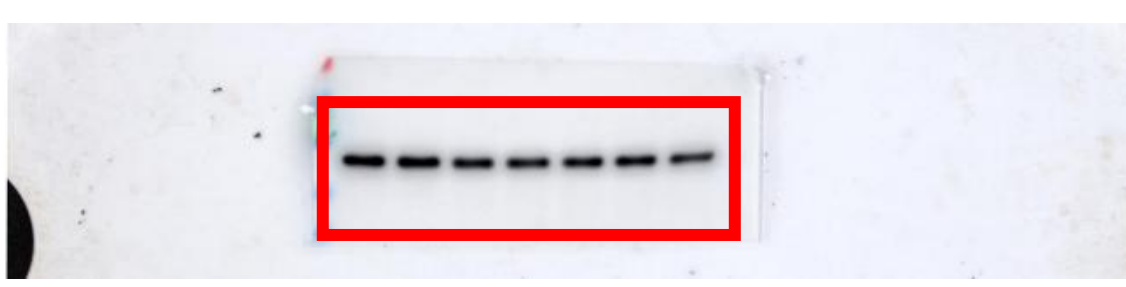

NUPR1

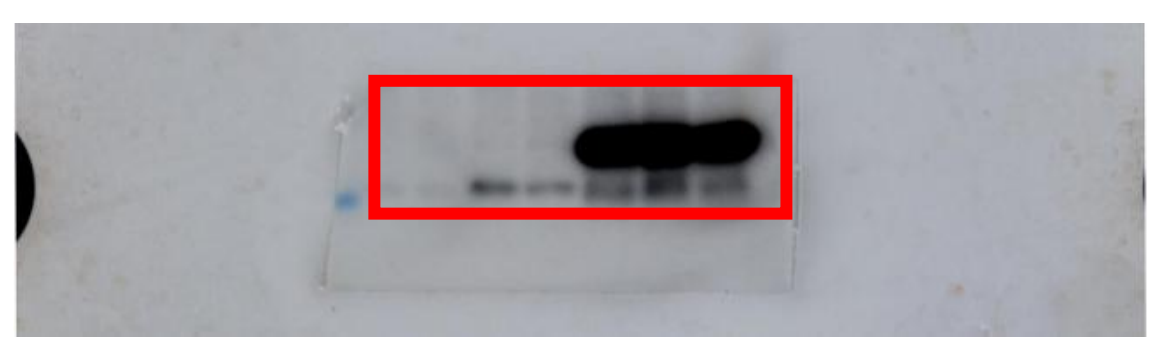

NUPR1

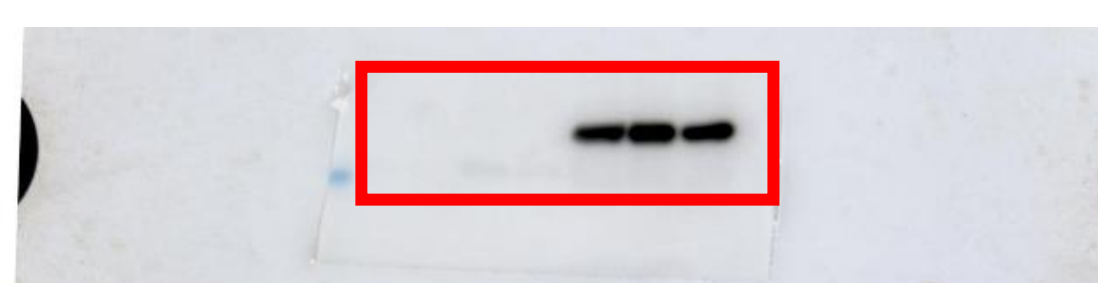

Figure S6B

GAPDH

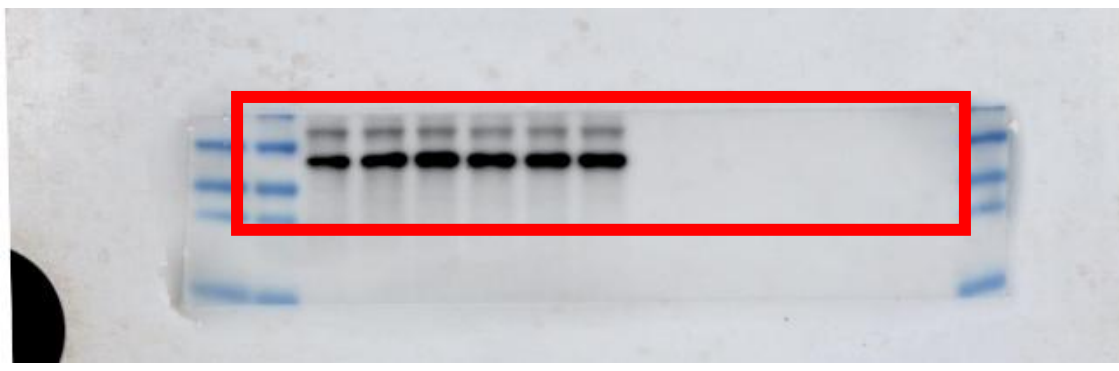

H3

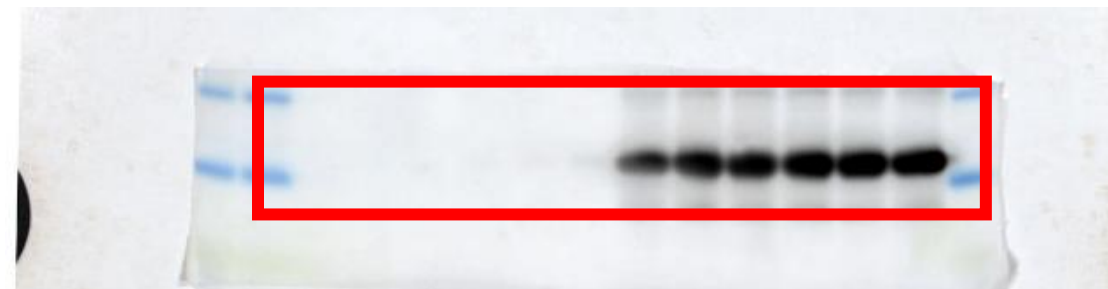

NUPR1

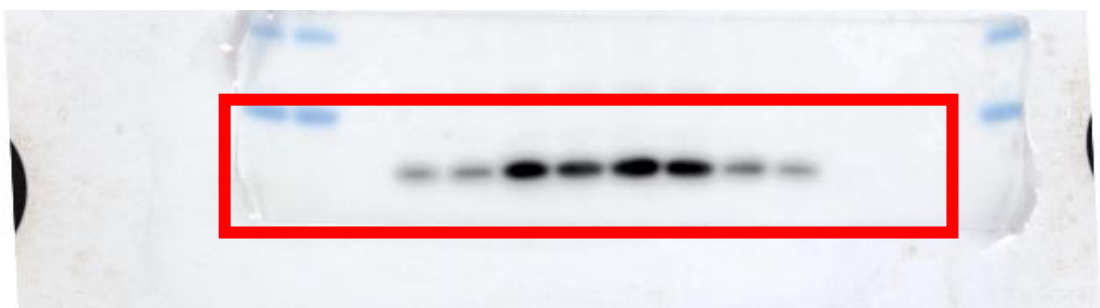

Figure S7A

GAPDH

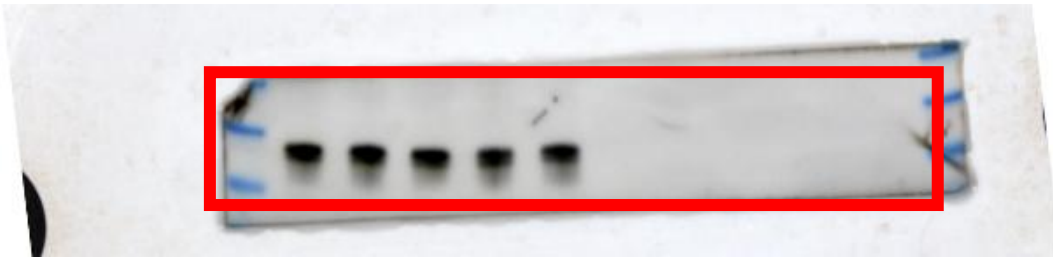

H3

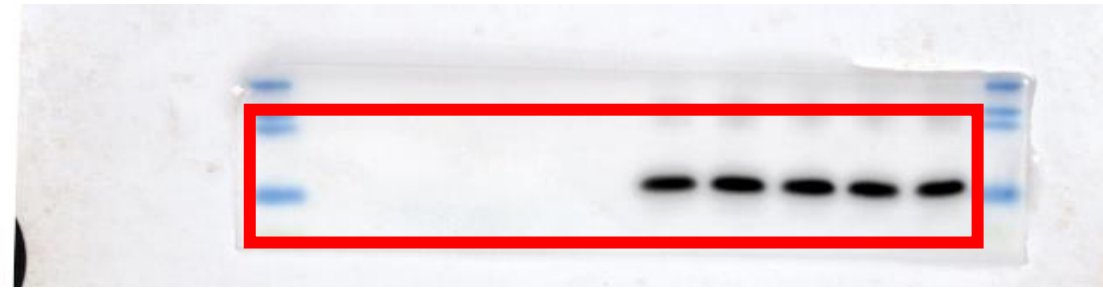

NUPR1

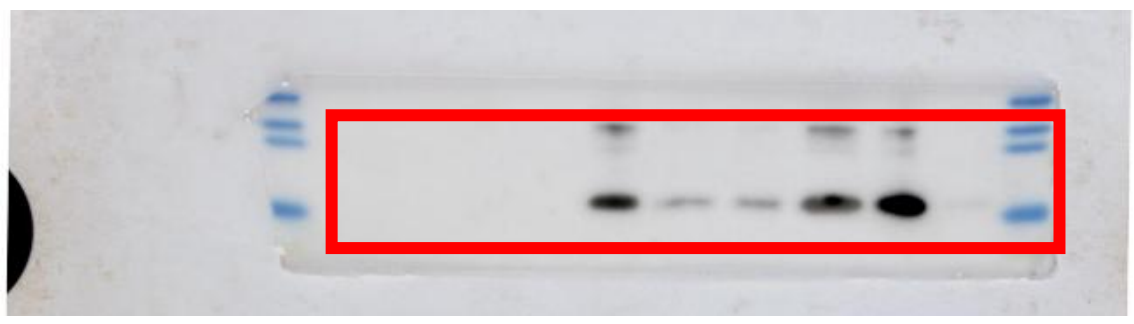

Figure S7B

NUPR1

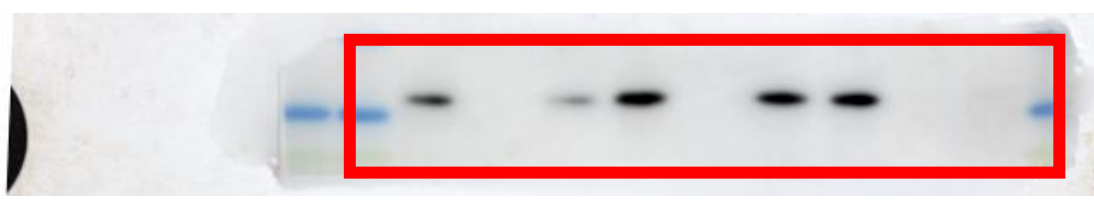

Figure S7C

Cyclin B2

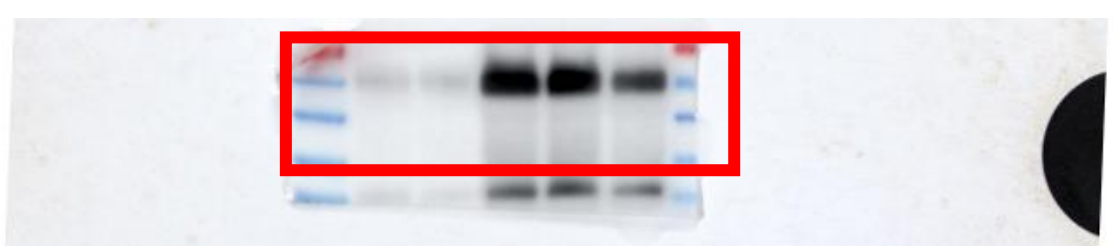

GAPDH

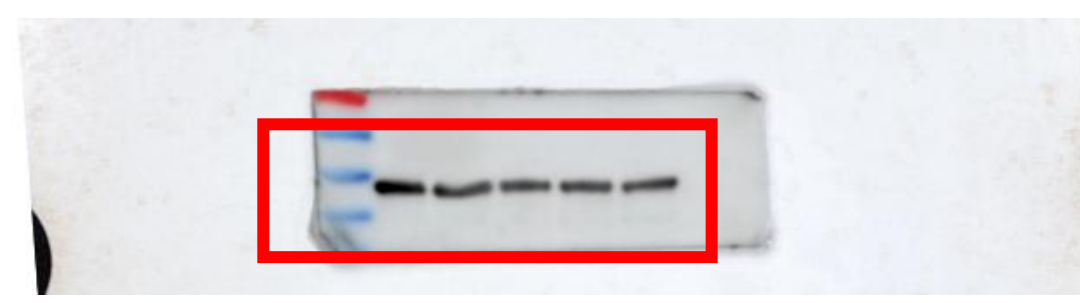

NUPR1

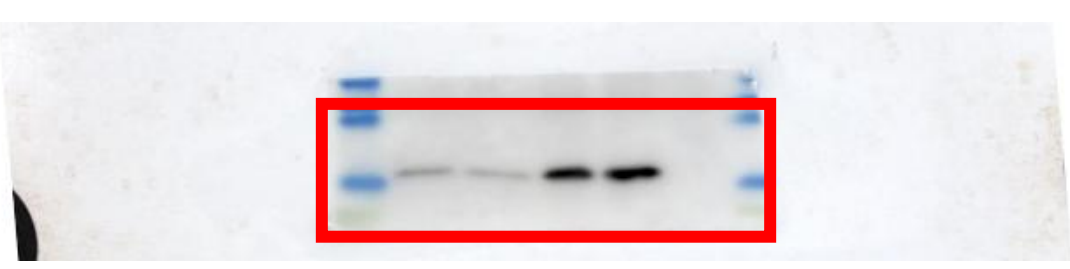

H3

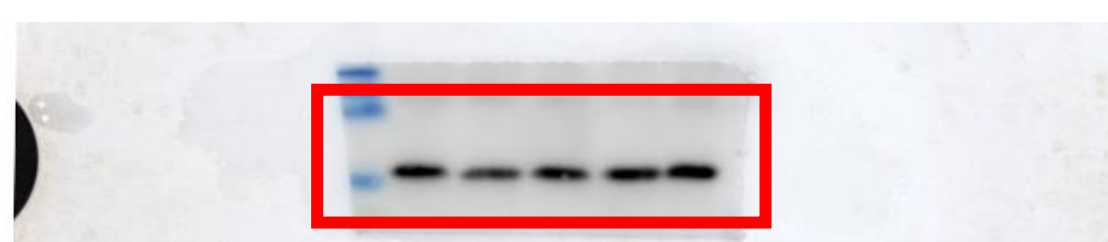

Figure S7H

NUPR1

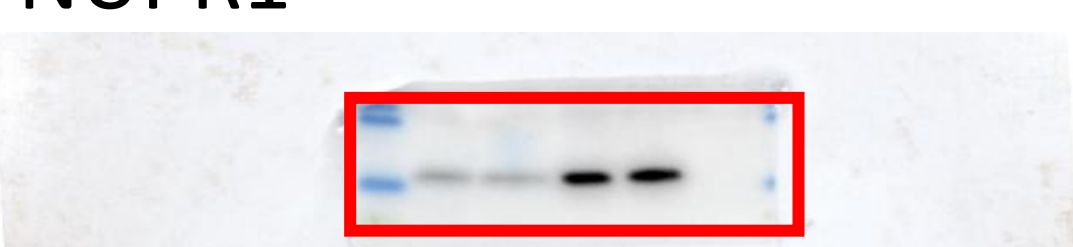

H3

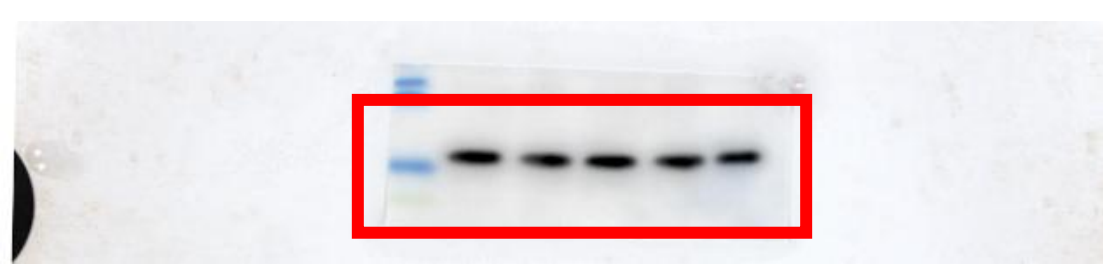

Cleaved-Caspase3

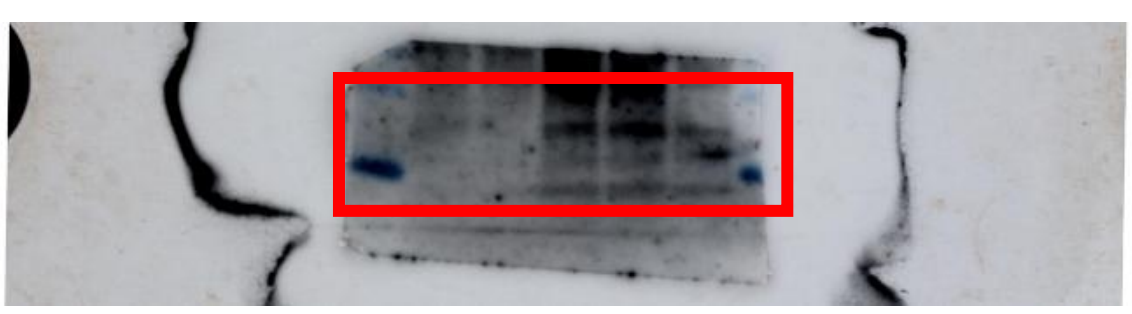

Caspase3

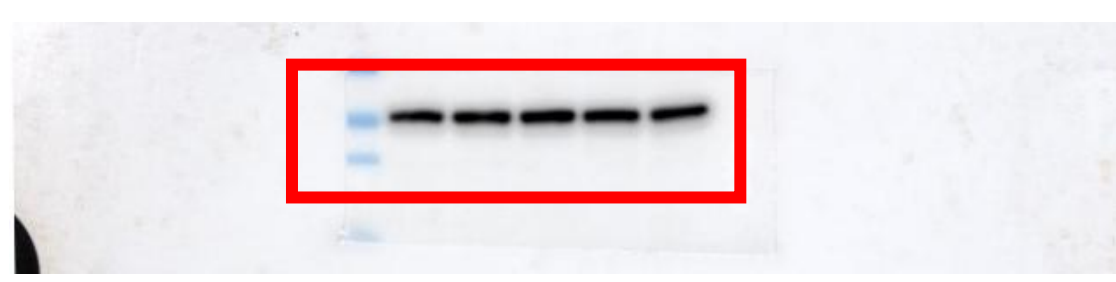

GAPDH

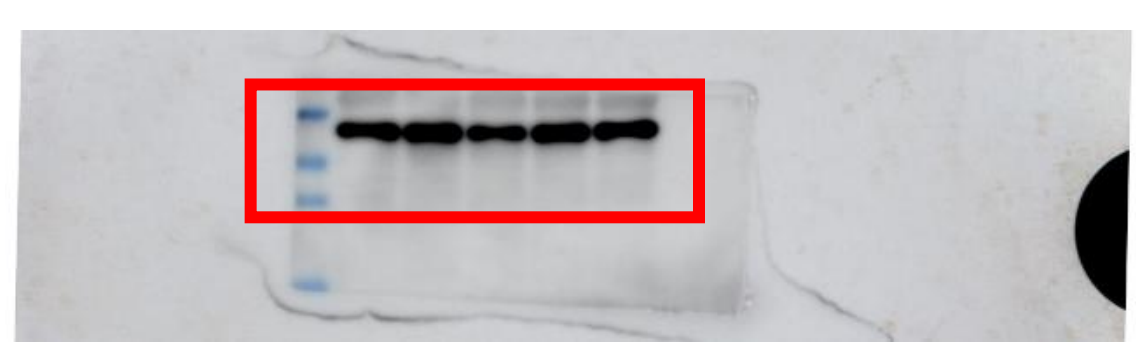

Figure S7J

GAPDH

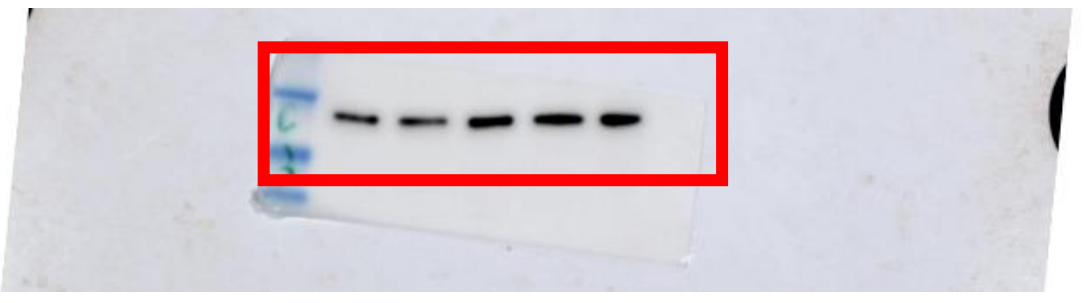

Nephrin

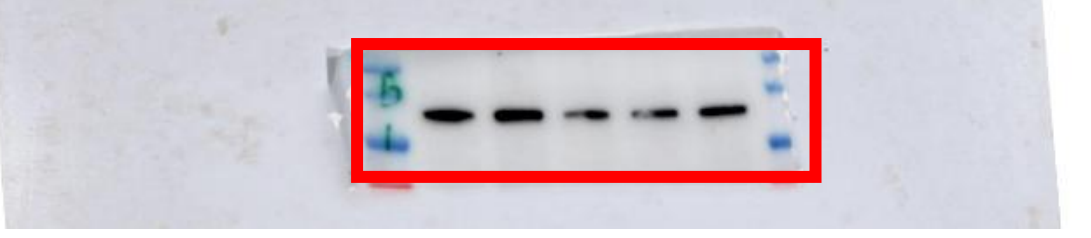

Podocin

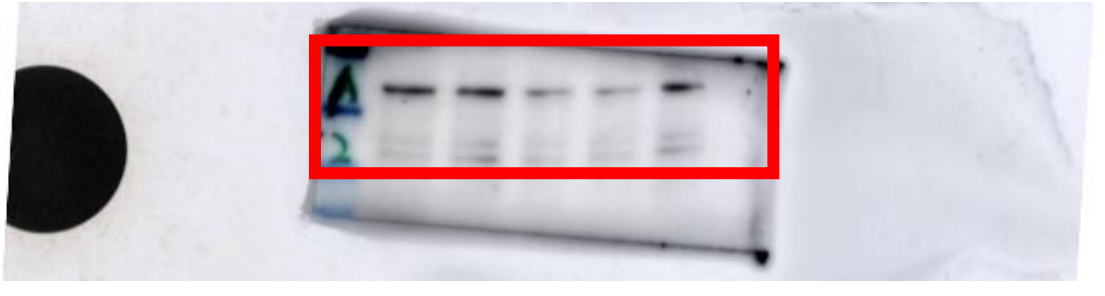

H3

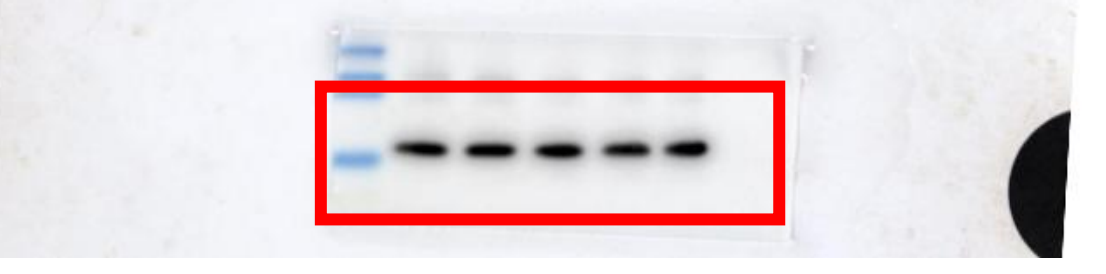

NUPR1

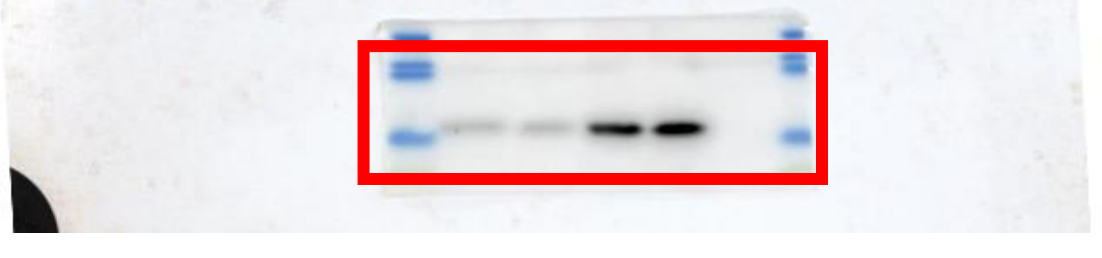

Figure 8C

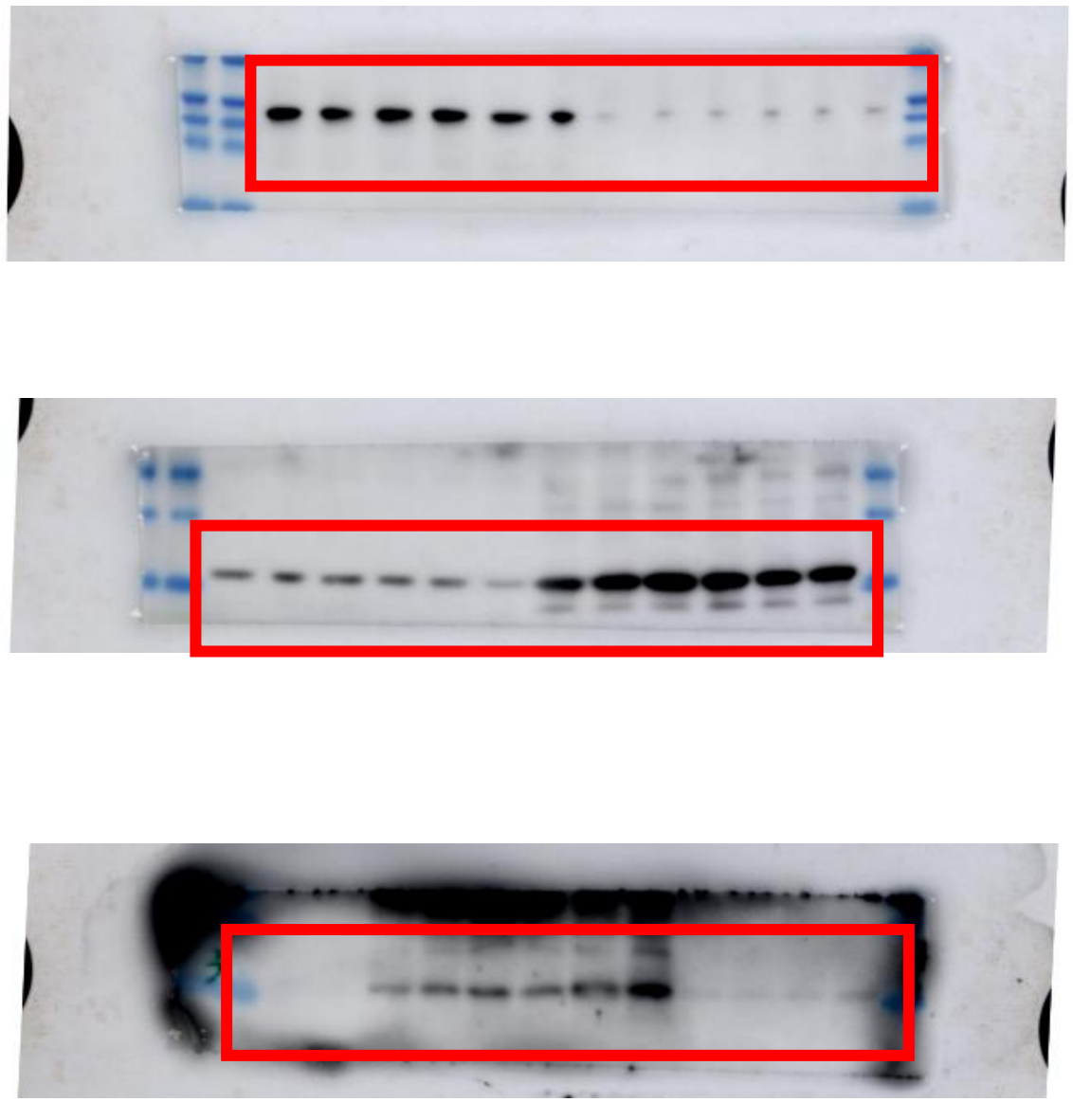

GAPDH

H3

NUPR1

Figure S8G

GAPDH

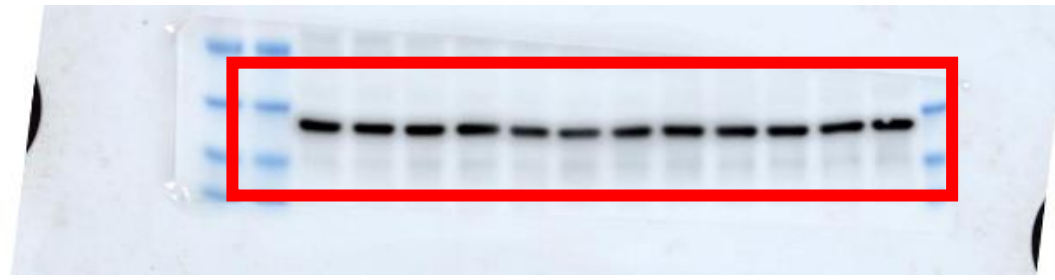

Nephrin

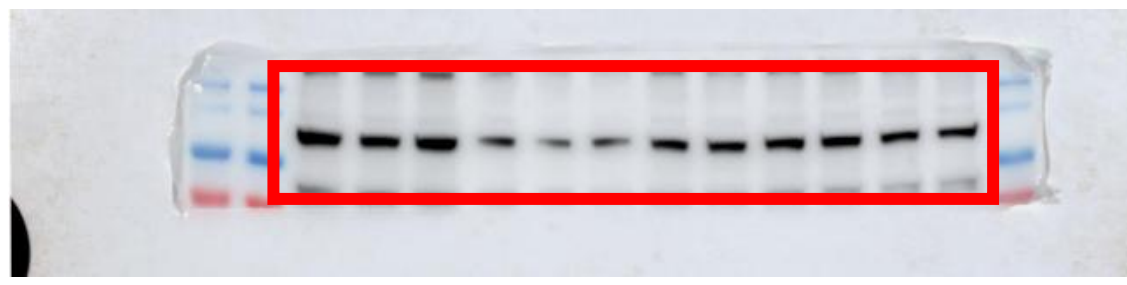

NUPR1

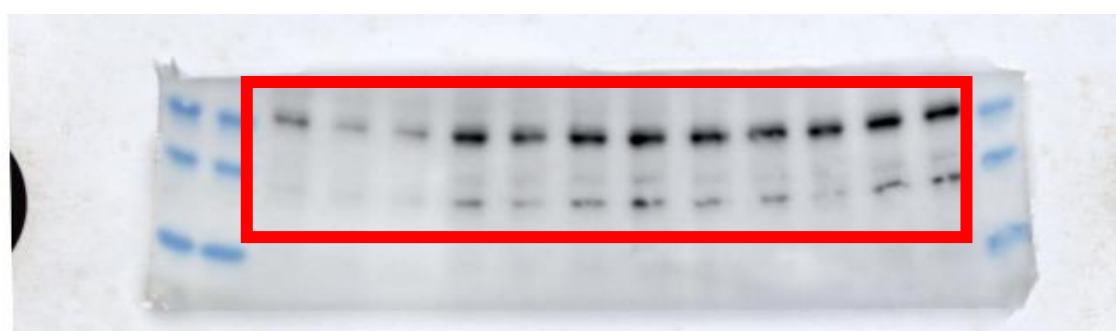

Podocin

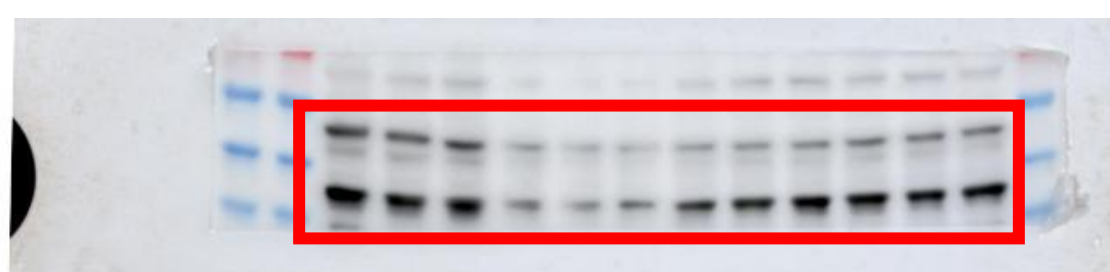

Figure S8H

Cyclin B2

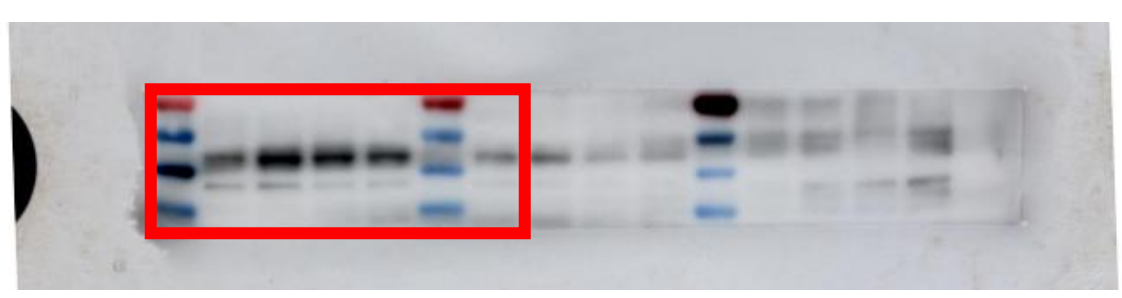

GAPDH

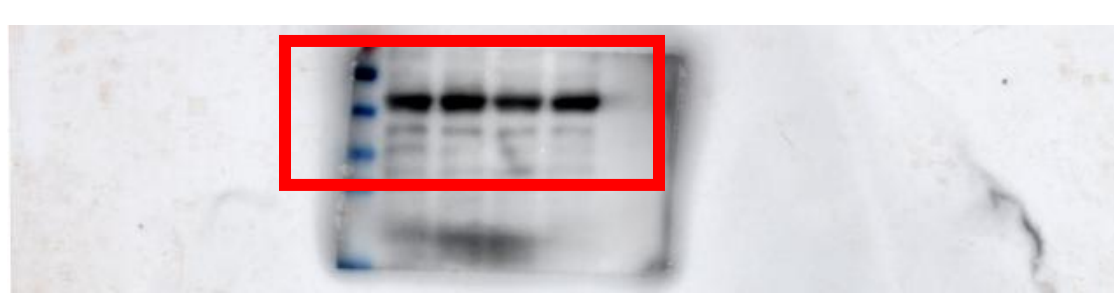

Supplement: Supplementary file 1 [file 41419_2026_9152_MOESM3_ESM.pdf]
