# Supplementary material for: NUPR1 inhibition prevents mitotic catastrophe: a novel therapeutic strategy for podocyte injury in diabetic kidney disease
Source: Cell Death Dis. 2026 Aug 12;17(1):950. doi: 10.1038/s41419-026-09152-8 (PMC13745598; doi:10.1038/s41419-026-09152-8)
Supplement: Supplementary file 2 [file 41419_2026_9152_MOESM1_ESM.docx]

# **Supplementary complete materials and methods**

***Renal Tissue Samples***

After written informed consent was obtained, 40 renal biopsy samples that were diagnosed with diabetic kidney disease (DKD) were collected from the Department of Nephrology at Guangdong Provincial People’s Hospital. Additionally, 10 normal control samples were obtained from the adjacent non-tumorous renal tissues of patients with no history of diabetes or renal impairment who underwent nephrectomy for renal tumors. The control samples were obtained from the Urology Department of the same institution. The clinical characteristics of the 50 participants are summarized in Supplementary Table 2. This study was conducted in accordance with the Declaration of Helsinki and approved by the Research Ethics Committee of Guangdong Provincial People’s Hospital (Approval No. KY2024-1074-02).

***Experimental Animals***

All the animal experiments were conducted in compliance with the Guide for the Care and Use of Laboratory Animals and were approved by the Research Ethics Committee of Guangdong Provincial People’s Hospital (Approval No. KY2024-1074-02).

Male 6-week-old C57BL/6 mice were obtained from the Guangdong Medical Laboratory Animal Center. Because of the reported increased resistance of female mice to type 2 diabetes, only male mice were included in this study. The mice were randomly assigned to different groups, and all experiments were conducted with 6 mice per group. The investigators were blinded to the group assignments during both the surgical procedures and outcome assessments.

All animals were housed under standard conditions (12-hour light/dark cycle, 24 °C), with 3–5 mice per cage. The autoclaved cages with corncob bedding were changed three times per week, and autoclaved water and food were provided ad libitum. Mouse breeding and surgical procedures were performed under barrier-maintained conditions, with continuous veterinary monitoring.

***Establishment of the Diabetic Kidney Disease Mouse Model***

The DKD model was established in unilateral nephrectomy (UNX) mice via a combination of low-dose streptozotocin (STZ; Sigma, S0130) and a high-fat diet (HFD; 60% kcal from fat; Research Diets, D12492) as previously described^1^. This approach promoted the development of glomerulopathy and proteinuria.

After one week of acclimatization, 7-week-old mice underwent UNX, and control mice underwent sham surgery. At 8 weeks of age, the mice were fasted for 4 h and then intraperitoneally injected with low-dose STZ (50 mg/kg daily for 5 consecutive days) dissolved in 50 mmol/L sodium citrate buffer (pH 4.5). The control mice received vehicle injections.

UNX mice were maintained on a HFD until 24 weeks of age, whereas control mice were fed a standard diet (Research Diets, D12450J). All the animals had free access to food and water throughout the study period.

Blood glucose levels were monitored, and urine samples were collected at 8, 16, and 24 weeks of age for biochemical analysis. At the endpoint (24 weeks of age), the mice were euthanized. Blood and kidney samples were collected for histopathological evaluation. Renal cortical tissues were dissected for protein and RNA extraction to support further experiments.

***Generation of NUPR1-Overexpressing Mice***

The adeno-associated viral vector (AAV9) for mouse NUPR1 overexpression (pHBAAV-NUPR1) and the corresponding negative control (pHBAAV-null) were obtained from Hanheng (Shanghai, China). Using a hydrodynamic gene delivery approach^2^, 150 μL of filtered and purified pHBAAV-NUPR1 or pHBAAV-null (5 × 10^9^ international units/μL) was injected via the tail vein. Preliminary studies confirmed significantly elevated protein expression in the renal parenchyma one month after injection, with no detectable toxicity observed in AAV-treated mice.

For the DKD model mice, AAV injections were performed via a procedure consistent with the method described above. Viral vectors (pHBAAV-NUPR1 or pHBAAV-null) were administered at 12 and 18 weeks of age.

***ZZW-115 Intervention Treatment***

ZZW-115 (Selleck Chemicals, E1037) was administered via intraperitoneal injection at doses of 5 or 10 mg/kg on the basis of previous pharmacological studies demonstrating its efficacy in vivo^3, 4^. Preliminary experiments indicated that ZZW-115 effectively inhibited the nuclear translocation and functional activity of NUPR1, with no signs of toxicity observed in the treated mice.

The treatment protocol for the DKD mice followed a procedure consistent with the aforementioned method. Beginning at 17 weeks of age, the DKD mice received intraperitoneal injections of ZZW-115 (5 or 10 mg/kg) every other day for a total duration of 8 weeks. The control mice were administered an equivalent volume of dimethyl sulfoxide (DMSO) (MP Biomedicals, 196055) on the same schedule.

At the start of ZZW‑115 treatment (17 weeks of age), the body weights (mean ± SD, n=6 per group) were as follows: CON group: 28.55 ± 1.25 g; DKD + DMSO group: 24.78 ± 1.67 g; DKD + ZZW‑115 (5 mg/kg) group: 25.03 ± 1.58 g; DKD + ZZW‑115 (10 mg/kg) group: 24.52 ± 2.11 g.

***Cell Culture and Treatment***

***Cell Sources and Culture Conditions***

All the cell lines were cultured under standard conditions at 37°C with 5% CO₂ unless otherwise specified. The sources and specific culture protocols are detailed below: (1) Conditionally immortalized mouse podocyte cells (MPCs) and human podocyte cells (HPCs) were kindly provided by Dr. Jochen Reiser (Rush University Medical Center, Chicago, IL, USA). The MPCs were maintained in RPMI-1640 (CORNING, 10-040-CVRC) supplemented with 10% FBS (CORNING, 35-081-CV) and 100 U/mL recombinant mouse interferon-gamma (IFN-γ; PROSPEC, cyt-358) at 33°C. Differentiation was induced by culturing the cells in RPMI-1640 supplemented with 5% FBS at 37°C for 10 days. HPCs were cultured in RPMI-1640 containing 10% FBS and insulin-transferrin-selenium (ITS-A; Gibco, 51300--044) at 33°C and differentiated in RPMI-1640 with 10% FBS at 37°C for 12 days. (2) Human renal tubular epithelial cells (HK-2) and mouse renal tubular epithelial cells (mRTECs) were provided by Prof. Lili Zhou (Southern Medical University, China). Both cell lines were cultured in DMEM-H (Gibco, C12430500BT) supplemented with 10% fetal bovine serum (FBS). (3) Human mesangial cells (HRMCs), mouse mesangial cells (SV40 MES), and human embryonic kidney cells (HEK 293T) were obtained from Prof. Ming Li (Guangdong Provincial People's Hospital, China). HRMCs were grown in EMEM (Gibco, C11885500) supplemented with 10% FBS, SV40 MES cells in specialized medium consisting of 71.25% DMEM-H, 23.75% F-12 (Gibco, 11765062), 5% FBS, and 14 mM HEPES (Selleck, S6831), and HEK293T cells in DMEM-H supplemented with 10% FBS. (4) Human renal glomerular endothelial cells (HRGECs) were gifted by Prof. Lixia Xu (Guangdong Provincial People's Hospital, China) and cultured in specialized medium for immortalized human glomerular endothelial cells (Procell, CM-H061) supplemented with 10% FBS. (5) Human pancreatic cancer cells (PANC-1) were provided by Prof. Rufu Chen (Guangdong Provincial People's Hospital, China) and maintained in DMEM-H supplemented with 10% FBS. (6) Mouse primary type II alveolar epithelial cells (pmATIIs) were gifted by Prof. Yiyu Deng (Guangdong Provincial People's Hospital, China) and cultured in DMEM-H containing 10% FBS.

***Treatments***

The cells were subjected to the following treatments: (1) High-glucose HG (30 mM; Sigma, G7021) for 72 h; normal glucose (CON, 5.3 mM) served as the control. (2) Osmotic control: mannitol (MA, 24.7 mM; Sigma, M9647) for 72 h. (3) NUPR1 inhibition: ZZW-115 (4 μM) for 72 h; an equivalent volume of DMSO was used as the vehicle control. (4) Lentiviral transduction: HEK 293T cells were co-transfected with the transfer constructs and packaging plasmids psPAX2 and pMD2.G. Fresh supernatant was used for infection, as previously described^5^. (5) siRNA transfection: Cells were transfected with 50 nM siRNA for 8 h before the medium was changed. The sequences are listed in Supplementary Table 3.

***Reagents and Antibodies***

The following chemical reagents were used in this study: STZ (Sigma, S0130), D-mannitol (Sigma, M9647), D-(+)-glucose (Sigma, G7021), ZZW-115 (Selleck, E1037), IFN-γ (PROSPEC, cyt-358), rat tail collagen I (CORNING, 354236), Triton X-100 (MP Biomedicals, 219485483), DMSO (MP Biomedicals, 196055), Fluoroshield™ with DAPI (Sigma, F6057), ITS-A (Gibco, 51300-044), HEPES (Selleck, S6831), Lotus Tetragonolobus Lectin (LTL; Vector Laboratories, FL-1321), and Acti-stain™ 555 Phalloidin (Cytoskeleton, PHDH1). Restriction enzymes were obtained from New England BioLabs Inc. (Beverly, MA, USA).

The primary and secondary antibodies used were as follows: anti-NUPR1 (Abcam, ab234696; Proteintech, 15056-1-AP), anti-β-tubulin (Immunoway, YM3030), anti-GAPDH (Bioworld, AP0063), anti-WT1 (Abcam, ab89901; Santa Cruz Biotechnology, sc-7385), anti-Vimentin (Proteintech, 10366-1-AP), anti-CD31 (Abcam, ab182981), anti-Podocin (Sigma, P0372), anti-Nephrin (Invitrogen, PA5-20330), anti-Synaptopodin (Santa Cruz Biotechnology, sc-515842), anti-phospho-Histone H3 (p-H3; Abcam, ab177218), anti-Ki-67 (Abcam, ab279653), anti-Cyclin B2 (Abcam, ab185622; Proteintech, 67726-1-Ig), anti-cleaved Caspase-3 (CST, 9664S), anti-Caspase-3 (CST, 9662S), anti-BRD2 (Abcam, ab245436; CST, D89B4), anti-Histone H3 (Abcam, ab1791), horseradish peroxidase (HRP)-conjugated goat anti-rabbit IgG (Jackson ImmunoResearch, 111-035-003), HRP-conjugated goat anti-mouse IgG (Jackson ImmunoResearch, 115-035-003), Alexa Fluor® 555-conjugated anti-rabbit IgG (CST, 4413S), Alexa Fluor® 488-conjugated anti-rabbit IgG (CST, 4412S), Alexa Fluor® 647-conjugated anti-rabbit IgG (CST, 4414S), Alexa Fluor® 555-conjugated anti-mouse IgG (CST, 4409S), Alexa Fluor® 488-conjugated anti-mouse IgG (CST, 4408S), Alexa Fluor® 647-conjugated anti-mouse IgG (CST, 4410S), normal mouse IgG (CST, 7076S), and normal rabbit IgG (CST, 7074S).

***RNA Extraction and Reverse Transcription Quantitative PCR***

Total RNA was extracted from the cells or tissues via TRIzol™ reagent (Invitrogen, 15596026). Reverse transcription quantitative polymerase chain reaction (RT‒qPCR) was performed to determine the mRNA expression levels, as described previously^6^. GAPDH was used as an endogenous reference gene for normalization. The sequences of all the primers used are listed in Supplementary Table 4.

***Western Blot Analyses***

Western blot analysis was carried out following established procedures^6^. Briefly, the tissues or cells were lysed in RIPA buffer (Beyotime, P0013B) and incubated on ice for 15 min. The homogenates were subsequently centrifuged at 12,000 × g for 15 min at 4°C. The resulting supernatant was collected, and the protein concentration was determined via a BCA assay.

Proteins were separated by 10% or 12% SDS‒PAGE and subsequently transferred to PVDF membranes. The membranes were probed with specific primary antibodies against the target proteins. GAPDH and β-tubulin were used as loading controls for normalization. Protein band intensity was quantified via ImageJ software.

***Cell Counting Kit-8 (CCK-8) Assay***

Podocyte viability and proliferation were assessed using a Cell Counting Kit-8 (Fdbio science, FD3788). Briefly, cells were seeded at a density of 5×10³ cells per well, after treatment, incubated with the CCK-8 reagent for 1-4 hours. Absorbance was measured at 450 nm.

***Flow Cytometry for Cell Cycle Analysis***

The cell cycle distribution was analyzed via a commercial cell cycle detection kit (Absin, abs50005). Briefly, mouse podocytes were fixed with 75% ethanol and stored overnight at 4°C. The following day, the cells were washed twice with PBS and incubated with RNase A and propidium iodide (PI) at 37 °C for 30 min in the dark. The samples were analyzed immediately via a flow cytometer (CytoFLEX, Beckman Coulter), and the data were processed via FlowJo software.

***Flow Cytometry for Apoptosis Analysis***

Apoptosis was assessed via a commercial Annexin V-FITC/PI apoptosis detection kit (KeyGEN, KGA108). Briefly, the cells were collected and adjusted to a density of 1 × 10⁶ cells/mL in binding buffer. The cell suspension was incubated with fluorescein isothiocyanate (FITC)-conjugated Annexin V and propidium iodide (PI) at room temperature for 15 min in the dark. The samples were analyzed immediately via a flow cytometer (CytoFLEX, Beckman Coulter), and the resulting data were processed via FlowJo software.

***Nuclear-Cytoplasmic Fractionation***

Nuclear and cytoplasmic protein extraction were performed via a commercial kit (Beyotime, P0028). Briefly, 1 × 10⁶ cells were collected, washed with ice-cold PBS, and lysed sequentially with cytoplasmic extraction buffers A and B on ice. The cytoplasmic fraction was then isolated via centrifugation. The remaining pellet was lysed via nuclear extraction buffer and centrifuged to obtain the nuclear fraction. All procedures were performed on ice in the presence of protease inhibitors. The efficiency of subcellular fractionation was verified by western blot analysis prior to subsequent experiments.

***Co-immunoprecipitation (Co-IP)***

Co-IP was performed according to established protocols^6^. Briefly, the cell lysates were incubated with specific primary antibodies or normal rabbit/mouse IgG (negative control) overnight at 4°C. The mixtures were incubated with Dynabeads™ Protein G at 4°C for 4 h. After thorough washing, the bound immunocomplexes were eluted and analyzed via western blotting.

***Luciferase Reporter Assay***

To evaluate the transcriptional regulation of Cyclin B2 by NUPR1 and BRD2, a 2000-bp fragment spanning the promoter region (–2000 to +1) was cloned and inserted into the pLV3-Fluc luciferase reporter vector. The lentivirus used for the reporter assays was produced by co-transfecting HEK 293T cells with the transfer construct and packaging plasmids psPAX2 and pMD2.G. The target cells were infected with lentiviral particles for 48 h. Luciferase activity was measured via a Dual-Luciferase® Reporter Assay Kit (RG027; Beyotime). The firefly luciferase signal was normalized to that of Renilla luciferase to account for variations in transfection efficiency.

***Chromatin Immunoprecipitation (ChIP) and Sequential ChIP (ChIP-re-ChIP)***

ChIP assays were conducted via a commercial SimpleChIP® Enzymatic Chromatin IP Kit (CST, 9003) according to the manufacturer’s instructions and previously established methods^7^. Briefly, the cross-linked chromatin was digested with micrococcal nuclease and sheared via sonication. The resulting chromatin fragments were immunoprecipitated with specific primary antibodies or normal rabbit/mouse IgG (negative control) at 4°C for 4 h. The immunoprecipitated DNA was purified and analyzed via RT‒qPCR.

For the ChIP-re-ChIP experiments, which were performed as described previously^8^, the complexes from the first immunoprecipitation were eluted under mild conditions. The eluate was then subjected to a second round of immunoprecipitation using a different antibody and the corresponding beads without reverse cross-linking. Finally, the DNA was purified via reverse cross-linking. All the sequences of primers used in the ChIP assays are listed in Supplementary Table 5.

***Biochemical Analysis of Serum*** ***and Urine Samples***

Body weight, kidney weight, and blood glucose levels were monitored in all the mice. The urinary albumin (ALB) concentration was determined via a commercial mouse ALB enzyme-linked immunosorbent assay (ELISA) kit (Beyotime, P0028) following the manufacturer's instructions. Serum and urinary creatinine levels were measured via a commercial creatinine assay kit (BioAssay Systems, DICT-500) according to standard protocols. Serum LDH concentration was measured using a commercially available Lactate Dehydrogenase Assay Kit (njjcbio, A020-2-2) in accordance with the manufacturer's protocol. Serum ALT and AST levels were determined using corresponding commercial kits for Alanine Aminotransferase (njjcbio, C009-2-1) and Aspartate Aminotransferase (njjcbio, C010-2-1), respectively.

***Renal Histological Analysis***

Renal histology was evaluated via periodic acid–Schiff (PAS) staining, as previously described^6^. Briefly, mouse kidney tissues were fixed overnight in 4% paraformaldehyde, embedded in paraffin, and sectioned at a thickness of 3 μm. PAS staining was performed via a commercial kit (Solarbio, G1281) according to the manufacturer’s protocol. A semi-quantitative assessment of the stained sections was conducted in a blinded manner. Positively stained areas were quantified via ImageJ software.

***Immunohistochemistry (IHC)***

Paraffin-embedded mouse kidney sections were prepared via standard histological procedures. Immunohistochemical staining was performed in strict accordance with the manufacturer’s protocol (ZSGB-BIO; ZLI-9019). The tissue sections were incubated with specific primary antibodies at 4°C overnight, followed by incubation with appropriate secondary antibodies at room temperature for 1 h. After development with 3,3′-diaminobenzidine (DAB) and counterstaining with hematoxylin, the positively stained areas were quantitatively analyzed via Image-Pro Plus software (Media Cybernetics, USA).

***Immunofluorescence (IF) Staining***

Paraffin-embedded mouse kidney sections were prepared according to standard histological protocols. The sections were incubated with specific primary antibodies at 4°C overnight, followed by incubation with Alexa Fluor® 488-, 555-, or 647-conjugated secondary antibodies. Nuclei were counterstained with DAPI-containing mounting medium. Images were captured via a laser scanning confocal microscope (Nikon, Tokyo, Japan), and fluorescence-positive areas were quantified via ImageJ software.

***Transmission Electron Microscopy (TEM)***

TEM was performed as previously described^9^. Briefly, the tissue samples were fixed in 4% glutaraldehyde at 4°C and subsequently post-fixed in 1% osmium tetroxide at 4°C. After dehydration via a graded ethanol series, the samples were embedded in epoxy resin and ultrathinly sectioned at a thickness of 70 nm. The sections were stained with 5% uranyl acetate and examined under a transmission electron microscope (H-7000, Hitachi, Tokyo, Japan). The acquired images were analyzed via ImageJ software.

***RNA Sequencing***

RNA Sequencing Analysis was performed as previously described^6^. Total RNA was extracted from cultured podocytes via TRIzol reagent. Sequencing libraries were constructed and subjected to high-throughput transcriptome analysis following established protocols. Differentially expressed genes (DEGs) were identified using the thresholds of |log₂(fold change)| > 1 and an adjusted p value (q value) < 0.05. Functional enrichment analysis of DEGs was performed via the Kyoto Encyclopedia of Genes and Genomes (KEGG) and Gene Ontology (GO) databases.

All RNA-seq data generated in this study have been deposited in the China National Center for Bioinformation/Beijing Institute of Genomics, Chinese Academy of Sciences (GSA: CRA031396). These data are publicly accessible at <https://ngdc.cncb.ac.cn/gsa-human>.

***Single-cell RNA (scRNA) Sequencing***

Kidneys were harvested from 22 weeks db/m and db/db mice under deep anesthesia. The renal capsules were removed, and the renal tissues were minced and digested in a dissociation enzyme solution at 37°C for 15-20 minutes to generate a single-cell suspension. The resulting cell suspension was filtered through a 40-μm cell strainer and centrifuged to collect the cells. Red blood cells were lysed, and the viability and concentration of the cells were assessed using Trypan Blue exclusion. According to the manufacturer's instructions for the 10x Genomics Chromium platform, a single-cell suspension with over 90% viability was loaded to construct barcoded scRNA-seq libraries. The libraries were then sequenced on an Illumina NovaSeq 6000 with 150 bp paired end reads.

All scRNA-seq data generated in this study have been deposited in the China National Center for Bioinformation/Beijing Institute of Genomics, Chinese Academy of Sciences (GSA: CRA031158). These data are publicly accessible at <https://ngdc.cncb.ac.cn/gsa-human>.

***Statistical Analysis***

The data are presented as the means ± standard deviations (SDs) from a minimum of three independent experiments. Differences between two groups were assessed via a two-tailed unpaired Student’s t test. Comparisons among multiple groups were performed via one-way or two-way analysis of variance (ANOVA), followed by Bonferroni post hoc correction. All tests were two sided. Analyses were performed via SPSS 16.0. A p value of less than 0.05 was considered statistically significant (*p < 0.05, **p < 0.01, ***p < 0.001; NS, not significant).

# **Supplementary Figures**

**Supplementary Figure 1.** **NUPR1 was upregulated in podocytes of diabetic kidney disease mice and patients.**

(A) Representative immunofluorescence images of NUPR1 and LTL in kidney sections from control mice and STZ/UNX/HFD DKD model mice. Scale bars: 10 μm. (B) Representative immunofluorescence images of NUPR1 and vimentin in kidney sections from control mice and STZ/UNX/HFD DKD model mice. Scale bars: 10 μm. (C) Representative immunofluorescence staining of NUPR1 and CD31 in kidney sections from control mice and STZ/UNX/HFD DKD mice. Scale bars: 10 μm. (D) Representative images of immunofluorescence staining for NUPR1 and LTL in db/m and db/db diabetic mice. Scale bars: 10 μm. (E) Representative images of immunofluorescence staining for NUPR1 and vimentin in db/m and db/db diabetic mice. Scale bars: 10 μm. (F) Representative images of immunofluorescence staining for NUPR1 and CD31 in db/m and db/db diabetic mice. Scale bars: 10 μm. The data are presented as the means ± SDs from three independent experiments. *p < 0.05, **p < 0.01, ***p < 0.001; NS, not significant (Student’s t test).

**Supplementary Figure 2. NUPR1 aggravated HG-induced podocyte injury.**

(A) Relative mRNA levels of Synaptopodin, Podocin, Nephrin, WT1, and NUPR1 in podocytes treated with high glucose (HG) (30 mM) and MA (30 mM) for 72 h (n=3 per group). (B) Representative western blot images and quantitative analysis of NUPR1, Synaptopodin, and Podocin protein levels in podocytes treated with HG (30 mM) and MA (30 mM) for 72 h (n=3 per group). (C) Relative mRNA levels of NUPR1 in podocytes transduced with lentivirus-NUPR1 (n=3 per group). (D) Representative western blot images and quantitative analysis of NUPR1 protein levels in podocytes transduced with lentivirus-NUPR1 (n=3 per group). (E) Relative mRNA levels of Synaptopodin, Podocin, Nephrin, WT1, and NUPR1 in different podocyte groups (n=3 per group): Con, MA, HG, HG+Vector, HG+NUPR1. (F) Representative western blot images and quantitative analysis of NUPR1, Synaptopodin, and Podocin protein levels in the different podocyte groups (n=3 per group): Con, MA, HG, HG+Vector, HG+NUPR1. (G) Relative mRNA levels of NUPR1 in podocytes transfected with NUPR1-targeting siRNAs (siRNA1-3) (n=3 per group). (H) Representative western blot images and quantitative analysis of NUPR1 protein levels in podocytes transfected with NUPR1-targeting siRNAs (siRNA1-3) (n=3 per group). (I) Representative western blot images and quantitative analysis of NUPR1 protein levels in podocytes transfected with NUPR1-targeting siRNA1 (n=3 per group). (J) Relative mRNA levels of Synaptopodin, Podocin, Nephrin, WT1, and NUPR1 in different podocyte groups (n=3 per group): Con, MA, HG, HG+si-NC, HG+si-NUPR1. (K) Representative western blot images and quantitative analysis of NUPR1, Synaptopodin, and Podocin protein levels in the different podocyte groups (n=3 per group): Con, MA, HG, HG+si-NC, HG+si-NUPR1. The data are presented as the means ± SDs from three independent experiments. *p < 0.05, **p < 0.01, ***p < 0.001; NS, not significant (Student's t test).

**Supplementary** **Figure 3. NUPR1 regulated the cell cycle in podocyte injury of diabetic kidney disease.**

(A) Venn diagram showing overlapping genes. The data are presented as the means ± SDs from three independent experiments. *p < 0.05, **p < 0.01, ***p < 0.001; NS, not significant (Student’s t test).

**Supplementary Figure 4. Expression of Cyclin B2 under high-glucose conditions and construction of in vitro models with knockdown or overexpression of Cyclin B2.**

(A) Relative mRNA levels of Cyclin B2 in podocytes treated with high-glucose HG (30 mM) and MA (30 mM) for 72 h (n=3 per group). (B) Representative western blotting images and quantitative analysis of Cyclin B2 protein levels in podocytes treated with HG (30 mM) and MA (30 mM) for 72 h (n=3 per group). (C) Relative mRNA levels of Cyclin B2 in podocytes transfected with Cyclin B2-targeting siRNAs (siRNA1-3) (n=3 per group). (D) Representative western blot images and quantitative analysis of Cyclin B2 protein levels in podocytes transfected with Cyclin B2-targeting siRNAs (siRNA1-3) (n=3 per group). (E) Relative mRNA levels of Cyclin B2 in podocytes transfected with Cyclin B2-targeting siRNA1 (n=3 per group). (F) Representative western blot images and quantitative analysis of Cyclin B2 protein levels in podocytes transfected with Cyclin B2-targeting siRNA1 (n=3 per group). (G) Relative mRNA levels of Cyclin B2 in podocytes transduced with lentivirus-Cyclin B2 (n=3 per group). (H) Representative western blot images and quantitative analysis of Cyclin B2 protein levels in podocytes transduced with lentivirus expressing Cyclin B2 (n=3 per group). The data are presented as the means ± SDs from three independent experiments. *p < 0.05, **p < 0.01, ***p < 0.001; NS, not significant (Student’s t test).

**Supplementary Figure 5. NUPR1 regulated podocyte mitotic catastrophe by inducing Cyclin B2 expression in podocytes.**

(A) Flow cytometry analysis of the cell cycle distribution in different podocyte groups: si-NC+Vector, si-NC+Vector+MA, si-NC+Vector+HG, si-NUPR1+Vector+HG, si-NC+Cyclin B2, si-NC+Cyclin B2+HG, si-NUPR1+Cyclin B2+HG. (B) Quantitative assessment of the cell cycle distribution in different podocyte groups (n=3 per group): si-NC+Vector, si-NC+Vector+MA, si-NC+Vector+HG, si-NUPR1+Vector+HG, si-NC+Cyclin B2, si-NC+Cyclin B2+HG, si-NUPR1+Cyclin B2+HG. (C) Representative immunofluorescence staining for p-H3 and Ki-67 in different podocyte groups: si-NC+Vector, si-NC+Vector+MA, si-NC+Vector+HG, si-NUPR1+Vector+HG, si-NC+Cyclin B2, si-NC+Cyclin B2+HG, si-NUPR1+Cyclin B2+HG. Scale bars: 100 μm. (D) Quantitative analysis of representative immunofluorescence staining for p-H3 and Ki-67 in different podocyte groups (n=3 per group): si-NC+Vector, si-NC+Vector+MA, si-NC+Vector+HG, si-NUPR1+Vector+HG, si-NC+Cyclin B2, si-NC+Cyclin B2+HG, si-NUPR1+Cyclin B2+HG. (E) Representative images of the actin cytoskeleton structure across the groups: si-NC+Vector, si-NC+Vector+MA, si-NC+Vector+HG, si-NUPR1+Vector+HG, si-NC+Cyclin B2, si-NC+Cyclin B2+HG, si-NUPR1+Cyclin B2+HG. Scale bars: 100 μm. (F) Quantitative analysis of binucleated podocytes across the groups (n=3 per group): si-NC+Vector, si-NC+Vector+MA, si-NC+Vector+HG, si-NUPR1+Vector+HG, si-NC+Cyclin B2, si-NC+Cyclin B2+HG, si-NUPR1+Cyclin B2+HG. (G) Flow cytometry analysis of apoptosis in different podocyte groups: si-NC+Vector, si-NC+Vector+MA, si-NC+Vector+HG, si-NUPR1+Vector+HG, si-NC+Cyclin B2, si-NC+Cyclin B2+HG, si-NUPR1+Cyclin B2+HG. (H) Quantitative evaluation of apoptosis in different podocyte groups (n=3 per group): si-NC+Vector, si-NC+Vector+MA, si-NC+Vector+HG, si-NUPR1+Vector+HG, si-NC+Cyclin B2, si-NC+Cyclin B2+HG, si-NUPR1+Cyclin B2+HG. The data are presented as the means ± SDs from three independent experiments. *p < 0.05, **p < 0.01, ***p < 0.001; NS, not significant (Student’s t test).

**Supplementary** **Figure 6. ZZW-115 attenuated podocyte injury and apoptosis by inhibiting mitotic catastrophe.**

(A) MPC cells were incubated with ZZW-115 at various concentrations for 72 h. Cell viability was examined by the CCK-8 assay in different podocyte groups (n=3 per group): 0μM, 1μM, 2μM, 4μM, 6μM, 8μM, 10μM, 12μM, 14μM, 16μM, 20μM. (B) Representative western blot images and quantitative analysis of the nuclear/cytoplasmic ratio of NUPR1 expression in the nuclear-cytoplasmic fractionation assay from different podocyte groups (n=3 per group): 0μM, 1μM, 2μM, 4μM, 6μM, 8μM. The data are presented as the means ± SDs from three independent experiments. *p < 0.05, **p < 0.01, ***p < 0.001; NS, not significant (Student's t test).

**Supplementary Figure 7. ZZW-115 attenuated podocyte injury and apoptosis by inhibiting mitotic catastrophe.**

(A) Representative western blot images and quantitative analysis of the nuclear/cytoplasmic ratio of NUPR1 expression in the nuclear-cytoplasmic fractionation assay from different podocyte groups (n=3 per group): Con, MA, HG, HG+DMSO, HG+4μM ZZW-115. (B) Co-immunoprecipitation (Co-IP) assay of NUPR1 and BRD2 interaction, group: Input, IP (IgG), IP (Anti-BRD2). (C) Representative western blot images and quantitative analysis of Cyclin B2 protein levels in the different podocyte groups (n=3 per group): Con, MA, HG, HG+DMSO, HG+4μM ZZW-115. (D) Flow cytometry analysis and quantitative assessment of the cell cycle distribution in different podocyte groups (n=3 per group): Con, MA, HG, HG+DMSO, HG+4μM ZZW-115. (E) Representative immunofluorescence staining for p-H3 and Ki-67 in different podocyte groups: Con, MA, HG, HG+DMSO, HG+4μM ZZW-115. Scale bars: 100 μm. (F) Quantitative analysis of representative immunofluorescence staining for p-H3 and Ki-67 in different podocyte groups (n=3 per group): Con, MA, HG, HG+DMSO, HG+4μM ZZW-115. (G) Representative images of the actin cytoskeleton structure and quantitative analysis of binucleated podocytes across the groups (n=3 per group): Con, MA, HG, HG+DMSO, HG+4μM ZZW-115. Scale bars: 100 μm. (H) Representative western blot images and quantitative analysis of cleaved caspase-3, and caspase-3 protein levels in the different podocyte groups (n=3 per group): Con, MA, HG, HG+DMSO, HG+4μM ZZW-115. (I) Flow cytometry analysis and quantitative evaluation of apoptosis in different podocyte groups (n=3 per group): Con, MA, HG, HG+DMSO, HG+4μM ZZW-115. (J) Representative western blot images and quantitative analysis of NUPR1, Nephrin, and Podocin protein levels in the different podocyte groups (n=3 per group): Con, MA, HG, HG+DMSO, HG+4μM ZZW-115. The data are presented as the means ± SDs from three independent experiments. *p < 0.05, **p < 0.01, ***p < 0.001; NS, not significant (Student's t test).

**Supplementary Figure 8. ZZW-115 alleviated podocyte injury and delayed diabetic kidney disease progression.**

(A) ALT levels in different groups of mice (n=6 per group): CON, DKD+DMSO, DKD+5mg/kg ZZW-115, DKD+10mg/kg ZZW-115. (B) AST levels in different groups of mice (n=6 per group): CON, DKD+DMSO, DKD+5mg/kg ZZW-115, DKD+10mg/kg ZZW-115. (C) LDH levels in different groups of mice (n=6 per group): CON, DKD+DMSO, DKD+5mg/kg ZZW-115, DKD+10mg/kg ZZW-115. (D) Blood glucose levels in different groups of mice (n=6 per group): CON, DKD+DMSO, DKD+5mg/kg ZZW-115, DKD+10mg/kg ZZW-115. (E) Kidney weights in different groups of mice (n=6 per group): CON, DKD+DMSO, DKD+5mg/kg ZZW-115, DKD+10mg/kg ZZW-115. (F) Body weights of the different groups of mice (n=6 per group): CON, DKD+DMSO, DKD+5mg/kg ZZW-115, DKD+10mg/kg ZZW-115. (G) Representative western blot images and quantitative analysis of NUPR1, Nephrin, and Podocin protein levels in renal cortex tissues from different groups of mice (n=6 per group): CON, DKD+DMSO, DKD+5mg/kg ZZW-115, DKD+10mg/kg ZZW-115. (H) Representative western blot images and quantitative analysis of Cyclin B2 protein levels in renal cortex tissues from different groups of mice (n=6 per group): CON, DKD+DMSO, DKD+5mg/kg ZZW-115, DKD+10mg/kg ZZW-115. (I) Representative images of Cyclin B2 and Synaptopodin immunofluorescence staining and quantitative analysis of kidney sections from different groups of mice (n=6 per group): CON, DKD+DMSO, DKD+5mg/kg ZZW-115, DKD+10mg/kg ZZW-115. Scale bars: 100 μm. The data are presented as the means ± SDs from three independent experiments. *p < 0.05, **p < 0.01, ***p < 0.001; NS, not significant (Student's t test).

# **References**

1.	Zhang L, Zhang Q, Liu S, Chen Y, Li R, Lin T*, et al.* DNA methyltransferase 1 may be a therapy target for attenuating diabetic nephropathy and podocyte injury. *Kidney international* 2017, **92**(1)**:** 140-153.

2.	Luo C, Zhou S, Zhou Z, Liu Y, Yang L, Liu J*, et al.* Wnt9a Promotes Renal Fibrosis by Accelerating Cellular Senescence in Tubular Epithelial Cells. *Journal of the American Society of Nephrology : JASN* 2018, **29**(4)**:** 1238-1256.

3.	Santofimia-Castaño P, Xia Y, Lan W, Zhou Z, Huang C, Peng L*, et al.* Ligand-based design identifies a potent NUPR1 inhibitor exerting anticancer activity via necroptosis. *The Journal of clinical investigation* 2019, **129**(6)**:** 2500-2513.

4.	Lan W, Santofimia-Castaño P, Swayden M, Xia Y, Zhou Z, Audebert S*, et al.* ZZW-115-dependent inhibition of NUPR1 nuclear translocation sensitizes cancer cells to genotoxic agents. *JCI insight* 2020, **5**(18).

5.	Wang L, Sun J, Yin Y, Sun Y, Ma J, Zhou R*, et al.* Transcriptional coregualtor NUPR1 maintains tamoxifen resistance in breast cancer cells. *Cell death & disease* 2021, **12**(2)**:** 149.

6.	Cai Y, Zhou J, Xu A, Huang J, Zhang H, Xie G*, et al.* N6-methyladenosine triggers renal fibrosis via enhancing translation and stability of ZEB2 mRNA. *The Journal of biological chemistry* 2024, **300**(9)**:** 107598.

7.	Wang JN, Yang Q, Yang C, Cai YT, Xing T, Gao L*, et al.* Smad3 promotes AKI sensitivity in diabetic mice via interaction with p53 and induction of NOX4-dependent ROS production. *Redox biology* 2020, **32:** 101479.

8.	Zhang H, Sun L, Liang J, Yu W, Zhang Y, Wang Y*, et al.* The catalytic subunit of the proteasome is engaged in the entire process of estrogen receptor-regulated transcription. *The EMBO journal* 2006, **25**(18)**:** 4223-4233.

9.	Li L, Li J, Li R, Zhao X, Chen Y, Cai Y*, et al.* Podocyte RIPK3 Deletion Improves Diabetic Kidney Disease by Attenuating NF-κB p65 Driven Inflammation. *Advanced science (Weinheim, Baden-Wurttemberg, Germany)* 2025, **12**(33)**:** e03325.
