# Supplementary material for: NUPR1 inhibition prevents mitotic catastrophe: a novel therapeutic strategy for podocyte injury in diabetic kidney disease
Source: Cell Death Dis. 2026 Aug 12;17(1):950. doi: 10.1038/s41419-026-09152-8 (PMC13745598; doi:10.1038/s41419-026-09152-8)
Supplement: Supplementary file 3 [file 41419_2026_9152_MOESM2_ESM.docx]

**Supplementary Table 1. Spearman correlation coefficients between NUPR1 expression and baseline clinical characteristics**

| Variable | Sample (n) | Spearman's r | *p* Value |
| --- | --- | --- | --- |
| Age | 40 | 0.0958 | 0.5565 |
| Height | 40 | -0.0042 | 0.9793 |
| Weight | 40 | 0.1161 | 0.4755 |
| Disease duration | 40 | 0.0276 | 0.8658 |
| Glycated hemoglobin | 40 | 0.0665 | 0.6835 |

**Supplementary Table 2. Clinical characteristics**

| Number | Gender | Age | Body weight (kg) | UACR (mg/gCr) | Scr (umol/L) | eGFR (ml/min*1.73m^2^) | Positive dots of  NUPR1 in podocytes |
| --- | --- | --- | --- | --- | --- | --- | --- |
| **Control Group(N=10)** | | | | | | | |
| 1 | M | 54 | 72 | NA | 110.47 | 64.9 | 3 |
| 2 | F | 48 | 56.5 | NA | 76.11 | 79.8 | 2 |
| 3 | M | 65 | 64 | NA | 95.5 | 71.6 | 4 |
| 4 | F | 64 | 52 | NA | 79.23 | 67.9 | 1 |
| 5 | M | 48 | 66 | NA | 91.79 | 84.7 | 4 |
| 6 | M | 74 | 59 | NA | 104.85 | 60.1 | 3 |
| 7 | M | 79 | 55 | NA | 91 | 68.8 | 4 |
| 8 | F | 65 | 58.5 | NA | 74.19 | 73 | 3 |
| 9 | M | 67 | 73 | NA | 79.98 | 87.5 | 2 |
| 10 | M | 38 | 58 | NA | 100.13 | 81.8 | 4 |
| **DKD Group(N=40)** | | | | | | | |
| 1 | M | 71 | 67 | 4264.96 | 258 | 20.6 | 13 |
| 2 | F | 63 | 58 | 347.53 | 88.28 | 60 | 5 |
| 3 | M | 39 | 53.7 | 3386.69 | 138.29 | 54.9 | 11 |
| 4 | F | 57 | 63 | 3715 | 109.12 | 48.4 | 13 |
| 5 | F | 42 | 50.38 | 4826.81 | 146.29 | 37.8 | 10 |
| 6 | M | 52 | 70 | 2136.94 | 167.88 | 39.7 | 8 |
| 7 | M | 58 | 66.4 | 4985.56 | 258.96 | 22.5 | 15 |
| 8 | M | 66 | 57 | 1422.15 | 168.81 | 35.7 | 9 |
| 9 | M | 43 | 73 | 2007.68 | 436.38 | 13.3 | 6 |
| 10 | M | 37 | 61 | 3489.52 | 255.79 | 26.5 | 10 |
| 11 | F | 67 | 60.7 | 1547.24 | 73.3 | 73.1 | 7 |
| 12 | F | 63 | 52 | 57.14 | 130.08 | 37.6 | 15 |
| 13 | F | 48 | 57 | 4380.68 | 83.84 | 71 | 7 |
| 14 | M | 54 | 62.3 | 1614.93 | 234.4 | 26.1 | 10 |
| 15 | M | 41 | 70 | 1253.82 | 237.07 | 28.2 | 11 |
| 16 | M | 67 | 61.5 | 367.85 | 692.87 | 6.4 | 9 |
| 17 | M | 65 | 57 | 97.83 | 228.35 | 25 | 10 |
| 18 | M | 50 | 65 | 4176.71 | 637.38 | 8 | 15 |
| 19 | M | 54 | 66.5 | 83.97 | 150.46 | 44.7 | 10 |
| 20 | F | 47 | 63.14 | 2164.04 | 133.89 | 40.6 | 10 |
| 21 | M | 46 | 65 | 5478.06 | 172.61 | 40 | 10 |
| 22 | M | 54 | 49.6 | 339.22 | 66.09 | 104.1 | 7 |
| 23 | M | 60 | 70 | 4105.4 | 134.86 | 48.9 | 12 |
| 24 | M | 53 | 62 | 3126.85 | 94.5 | 78.9 | 9 |
| 25 | F | 35 | 78.5 | 1899.72 | 180.29 | 30.8 | 8 |
| 26 | M | 48 | 60 | 1707.16 | 321.15 | 18.6 | 7 |
| 27 | M | 45 | 72 | 7353.32 | 273.54 | 23.1 | 13 |
| 28 | M | 30 | 70 | 2459.19 | 82.2 | 109.8 | 10 |
| 29 | M | 71 | 55.9 | 2088.63 | 128.28 | 48.1 | 11 |
| 30 | M | 45 | 66.5 | 2028.02 | 161.81 | 44.5 | 9 |
| 31 | F | 43 | 39.6 | 939.74 | 294.38 | 16.1 | 8 |
| 32 | M | 47 | 65.2 | 1692.44 | 283.94 | 21.8 | 8 |
| 33 | M | 27 | 92.4 | 5721.7 | 109 | 79.7 | 11 |
| 34 | F | 39 | 53.9 | 735.56 | 181.84 | 29.6 | 8 |
| 35 | F | 58 | 61.5 | 5968.08 | 106.82 | 49.4 | 12 |
| 36 | M | 67 | 62.33 | 3017.97 | 758.62 | 5.8 | 10 |
| 37 | M | 47 | 60 | 2583.76 | 731.68 | 6.9 | 7 |
| 38 | M | 44 | 56 | 3445.39 | 226.2 | 29.3 | 12 |
| 39 | F | 30 | 63.5 | 6897.15 | 389.53 | 12.6 | 14 |
| 40 | M | 36 | 74.17 | 225.51 | 138.21 | 56.2 | 5 |
| Scr, serum creatinine; UACR, urinary albumin creatinine ratio; eGFR, estimated glomerular filtration rate. | | | | | | | |

**Supplementary Table 3. siRNA sequences**

| Name | Forward（5’–3’） | Reverse（5’–3’） |
| --- | --- | --- |
| si-Nupr1-1 | AGAGAAGCUGCUGCCAAUA(dT)(dT) | UAUUGGCAGCAGCUUCUCU(dT)(dT) |
| si-Nupr1-2 | CCAGAACUCUGAAAGGAAA(dT)(dT) | UUUCCUUUCAGAGUUCUGG(dT)(dT) |
| si-Nupr1-3 | GAAUCCUGGAUGAAUAUGA(dT)(dT) | UCAUAUUCAUCCAGGAUUC(dT)(dT) |
| si-Ccnb2-1 | AGAACGUUGUGAAAGUCAA(dT)(dT) | UUGACUUUCACAACGUUCU(dT)(dT) |
| si-Ccnb2-2 | GCAAGAUCGAGGACAUAGA(dT)(dT) | UCUAUGUCCUCGAUCUUGC(dT)(dT) |
| si-Ccnb2-3 | AAGUGACAAAUGUCAACAA(dT)(dT) | UUGUUGACAUUUGUCACUU(dT)(dT) |
| si-Brd2-1 | CACCGAUGAUAUUGUCCUA(dT)(dT) | UAGGACAAUAUCAUCGGUG(dT)(dT) |
| si-Brd2-2 | CUAUGUUUACCAACUGUUA(dT)(dT) | UAACAGUUGGUAAACAUAG(dT)(dT) |
| si-Brd2-3 | GGCUUCUGUACCAGCUUUA(dT)(dT) | UAAAGCUGGUACAGAAGCC(dT)(dT) |

**Supplementary Table 4. Primers for RT-qPCR**

| Gene | Forward（5’–3’） | Reverse（5’–3’） |
| --- | --- | --- |
| Gapdh | GCAAATTCAACGGCACAGTCAAG | TCGCTCCTGGAAGATGGTGATG |
| Nephrin | GTTCAGACCACACCAACATCCAG | GCTCACGCTCACAACCTTCAG |
| Podocin | CTGCCACCGCCACCGTAG | CCTCTGGTCGCTCGCTCTC |
| Synaptopodin | GTGAGGAAGAGGAAGTGCCATTG | GGGAGGGTTTGGTGAGGACTG |
| Wt1 | CCTGTCGCTACGGACCCTTC | CGGATGGTAGGCTGGCTCTC |
| Ccnb2 | GCTTTCTCTGATGCTCTGCTCTG | TGCCTGAGGTACTGGTAGATGTC |
| Nupr1 | ACCCTTCCCAGCAACCTCTAA | TCTTGGTCCGACCTTTCCGA |
| Cdk9 | AGCGGATTGACAGTGACGATGC | GGTTGGTGGTGGCGGGATTTC |
| Brd2 | TTCGCTATGCCAAGATGCCAGATG | CTTCTTCCTCCTCCTCCTCTTCCTC |

**Supplementary Table 5. Primers for ChIP**

| Gene | Forward（5’–3’） | Reverse（5’–3’） |
| --- | --- | --- |
| Cyclin B2-1 | ATGGTAGACTCCCTTGGCCT | CTCCAGCCCGACCTTGAATT |
| Cyclin B2-2 | TGAGTTGCCAAATTGGTGCTG | TGTATAGCCCTGGCAGTCCT |
| Cyclin B2-3 | CAAGAGTCAGCAGCCACAGA | GCAGTACTACCCCTCGACCT |
| Cyclin B2-4 | ACCTAGTCTTTTCCCCAGGGT | CAAATGACGGTGTGATGTGCA |
| Cyclin B2-5 | CCAGGTAGACGTTTCCGGAA | GGGAGCCATCACCTAAGGAC |
